# Association of probiotic supplementation and cardiovascular risk profiles of patients with coronary artery disease—a cross-sectional analysis of the NHANES database between 1999–2019

**DOI:** 10.3389/fnut.2025.1495633

**Published:** 2025-02-28

**Authors:** Murali Palathinkara, Michael Aljadah, Abigail Thorgerson, Aprill Z. Dawson, Michael E. Widlansky

**Affiliations:** ^1^Division of Cardiovascular Medicine, Department of Medicine, Medical College of Wisconsin, Milwaukee, WI, United States; ^2^Center for Advancing Population Science, Medical College of Wisconsin, Milwaukee, WI, United States; ^3^Division of General Internal Medicine, Department of Medicine, Medical College of Wisconsin, Milwaukee, WI, United States

**Keywords:** probiotic, coronary artery disease, NHANES, risk profiles, ASCVD

## Abstract

**Background:**

Atherosclerotic cardiovascular disease (ASCVD) is a chronic inflammatory disease that leads to adverse events such as myocardial infarctions and stroke. Gut microbiome modulation is a promising target to reduce chronic inflammation and improve outcomes for patients with coronary artery disease (CAD). Risk profile patterns of CAD patients who target gut health with probiotics could provide insight into how gut modulation improves CAD clinical biomarkers. This study aims to evaluate the association between probiotic use and clinical markers of known atherosclerotic risk factors, in patients with CAD.

**Methods:**

We conducted a cross-sectional large-database study using National Health and Nutrition Examination Survey (NHANES) data from years 1999–2020. The cohort included adults with at least a diagnosis of coronary heart disease, angina, and heart attack or two of the following: diabetes, high blood pressure, and high cholesterol. Analyses of clinical biomarkers compared probiotic to non-probiotic groups, between probiotic type groups, and between probiotic supplement strains.

**Results:**

Our cohort included 14,992 survey responders. After weighting, this sample represented 46,217,980 US adults. There were 4,062,022 adults exposed to probiotics, 763,288 to probiotic supplements and 3,179,008 to probiotic foods. Probiotic exposure was associated with lower A1c (*p* < 0.001), lower triglycerides (*p* < 0.001), lower ASCVD risk score (*p* = 0.01) and higher HDL-C (*p* < 0.001). Probiotic supplement exposure was associated with lower LDL-C (*p* = 0.003) and total cholesterol (*p* = 0.047).

**Conclusion:**

Our study reinforces the beneficial association between probiotic ingestion and cardiovascular health in patients with existing atherosclerotic disease. Further studies to better determine potential mechanistic connections between the gut microbiota on cardiovascular risk factors is warranted.

## Introduction

Atherosclerotic cardiovascular disease (ASCVD) is a chronic inflammatory disease that leads to adverse events such as myocardial infarctions, stroke, and limb ischemia (1). Collectively, mortality rates of these complications range from 6.3–11.3% annually, with coronary artery disease (CAD) as the leading cause of death in developed nations (1).

Therapies that reduce chronic inflammation and improve the traditional cardiovascular risk profile reduce atherosclerotic risk (2). One emerging target for reducing systemic inflammation is the gut microbiota (3, 4). Through gut microbial modulation, multiple beneficial physiologic pathways could result in improved cardiovascular physiology (5). A healthy gut microbiome supports the body’s immune system by directly competing for space and nutrients with microbial pathogens and indirectly by producing antimicrobial agents (3, 6, 7). Beneficial bacteria prevent pathogen translocation across the gut into the circulation and prevent pathogens from producing and releasing molecules such as bacterial fragments or lipopolysaccharides that can translocate and cause systemic inflammation (3, 6, 7). Other methods by which probiotics may contribute to metabolic disorders and ASCVD may include normalization of adipogenesis and regulation of insulin secretion, fat accumulation, energy homeostasis, and plasma cholesterol levels (7).

The cardiovascular risk profiles of patients with existing CAD who take supplemental probiotics compared to those who do not supplement could provide further insight into whether gut modulation is associated with risk factors for ASCVD that could drive additional events. Prior data have shown that probiotics are associated with reduced prevalence of obesity, diabetes, hypertension, and dyslipidemia in unselected individuals over 18 years of age within the NHANES database (8). However, whether probiotic ingestion is associated with improved cardiovascular risk factor profiles in those with established CAD or at elevated risk for CAD remains unknown. This is particularly important because patients with ASCVD or multiple cardiovascular risk factors commonly take medications, including ACE inhibitors and HMG-CoA reductase inhibitors, that can influence to composition of the gut microbiota (9). Patients with existing ASCVD or CAD are at higher risk of ASCVD events then the general population but within that group, patients can have variable risk for future ASCVD event (10). Controlling patients’ disease and lowering their ASCVD risk scores will help reduce their risk of future ASCVD events (11). If probiotics are shown to reduce ASCVD risk scores in patients with existing disease, they may serve as another option in clinicians’ lifestyle modification and pharmacologic toolbelt. In this study, we determined whether traditional cardiovascular risk profiles differed between those with CAD or elevated risk for CAD, and whether there were differences between probiotic supplementation compared to ingestion of foods high in probiotic content.

## Methods

### Data source

The data came from the National Health and Nutrition Examination Survey (NHANES). NHANES is a vital U.S. program that evaluates the health and nutritional well-being of both adults and children. This extensive survey gathers a wealth of demographic, socioeconomic, dietary, and health-related information through interviews, complemented by detailed medical, dental, and physiological examinations, including laboratory tests. NHANES findings play a critical role in gauging the prevalence of diseases and risk factors, evaluating nutritional status, and providing essential data for studies and health research to shape public health policies and services (12). The demographic, questionnaire, dietary, examination, and laboratory files were used across the 1999 – March 2020 years. The cohort was defined as adults (20 years and older) with at least one of the following: coronary heart disease, angina, and heart attack or at least two of the following: diabetes, high blood pressure, and high cholesterol. These were all self-report measures that NHANES deems reliable. Those with a diastolic blood pressure reading of 0 were excluded from the cohort. This included 14,992 subjects (46,217,980 weighted). As NHANES is a national database, the sample can be weighted to be representative of the US population, however the weights are created based on household information like age, race, and sex, not on probiotic use (12). This weightage, in addition to the fact that not all respondents answered the probiotic questions, results in slightly reduced sample (12). When the analyses are run, the weighted totals can be used. The weighted sample is represented by “N” in the manuscript tables.

### Measures

The primary independent variable was two category probiotic use. This was defined as taking a probiotic supplement (Category 1) or a probiotic food (Category 2). The lists of probiotic supplements and probiotic foods are in Supplementary Tables 7, 8. The outcomes were laboratory vitals: hemoglobin A1C, high density lipoprotein (HDL-C), low density lipoprotein (LDL-C), total cholesterol, triglyceride level, systolic blood pressure, and diastolic blood pressure. Body mass index (BMI) and ASCVD risk score were also treated as outcomes. All outcomes were continuous variables and treated as such throughout the analyses. The covariates included were age (continuous), sex (male, female), race/ethnicity (non-Hispanic white, non-Hispanic black, non-Hispanic other, Hispanic), marital status (married/partnered, not married), ratio of family income to poverty (continuous), education (less than high school, high school, and some college and higher), and metabolic equivalent (MET) score. The MET score was created by multiplying the number of minutes an activity was done with the recommended NHANES MET score from the physical activity questionnaire, then summing all items together (13, 14). The MET score was categorized into three categories according to tertiles (low, medium, and high). Medications from the questionnaire files were also included (ACE Inhibitor, Aldosterone Antagonist, Antiplatelet, Aldosterone Receptor Blockers, Beta Blockers, Dihydropyridine Calcium Channel Blockers, Digitalis, Diuretics, Direct Oral Anticoagulants, Ezetimibe, Loop Diuretic, non-Dihydropyridine Calcium Channel Blockers, Niacin, Nitrate, P2Y12 Inhibitor, Statins, Thiazides, and Vasodilator).

### Analyses

R v 4.0.3 was used for all analyses and an alpha level of 0.05 was chosen. All summary information and analyses were weighted using the survey package in R according to NHANES directions. Overall summary information of each variable was reported with mean and standard deviation for continuous variables and weighted percentage for categorical variables. Comparisons by probiotic use (yes/no) were done with 2-sample t-tests for continuous variables tand chi-square tests for categorical variables. Comparisons were then done between the type of probiotic use (supplement/food). Participants who reported exposure to both probiotic supplements and food were excluded from this probiotic-type comparison because of the small size of this group (4 participants). Comparisons were also done within the probiotic supplement group between the strains of bacteria. The probiotic supplements were grouped into three groups: probiotic ingredients that included solely *Lactobacillus, Lactobacillus* and *Bifidobacterium* as a combination (*Lactobacillus/Bifidobacterium combination*), and solely *Bifidobacterium* along with other strains not included in the previously mentioned groups (*Bifidobacterium/other*). The proportion of the cardiovascular population that used probiotics was also tracked and analyzed. Unadjusted linear regression models were created for each outcome (A1C, HDL-C, LDL-C, total cholesterol, triglyceride level, systolic blood pressure, diastolic blood pressure, body mass index (BMI), and ASCVD risk score) with probiotic use (yes/no) as the sole predictor. If the unadjusted model was significant, it was adjusted for age (continuous), sex (male, female), race/ethnicity (non-Hispanic white, non-Hispanic black, non-Hispanic other, Hispanic), marital status (married/partnered, not married), ratio of family income to poverty (continuous), education (less than high school, high school, and some college and higher), and MET score (categorical). Unadjusted linear regression models were also created for each outcome with probiotic type (supplement/food) as the sole predictor. Again, if the unadjusted model was significant, it was adjusted with age (continuous), sex (male, female), race/ethnicity (non-Hispanic white, non-Hispanic black, non-Hispanic other, Hispanic), marital status (married/partnered, not married), ratio of family income to poverty (continuous), education (less than high school, high school, and some college and higher), and MET score (categorical). Statistical significance was defined as a two-sided *p*-value <0.05.

## Results

### Baseline characteristics and probiotic exposure

**Figure 1:**
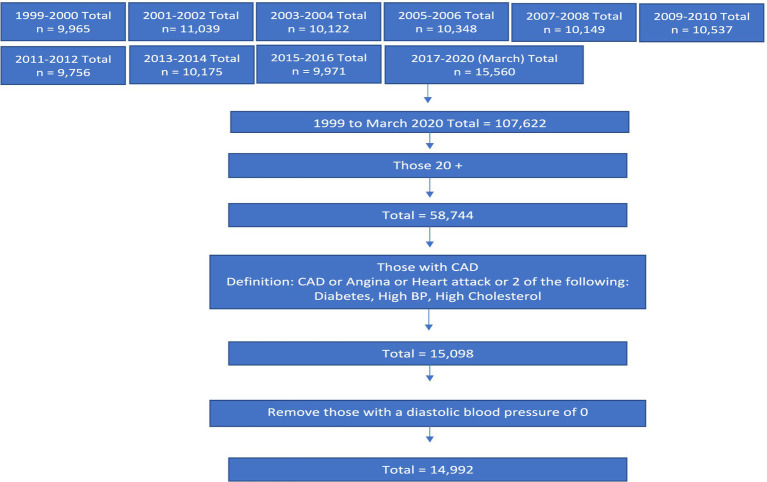
Participant flowchart.

**Figure 2:**
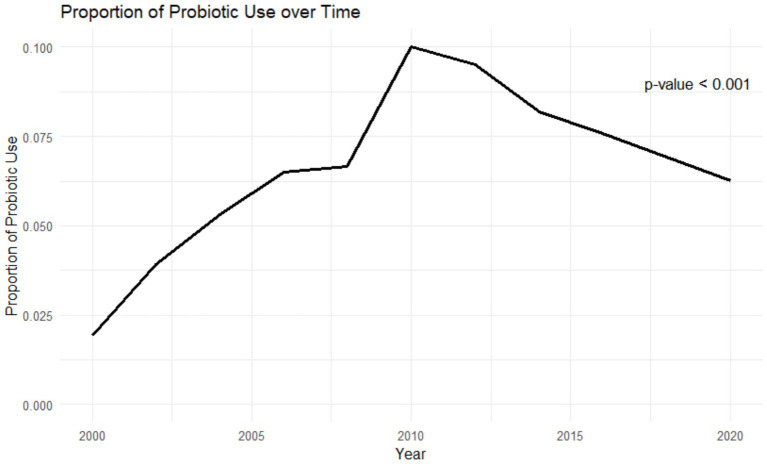
Average probiotic use over time.

**Figure 3:**
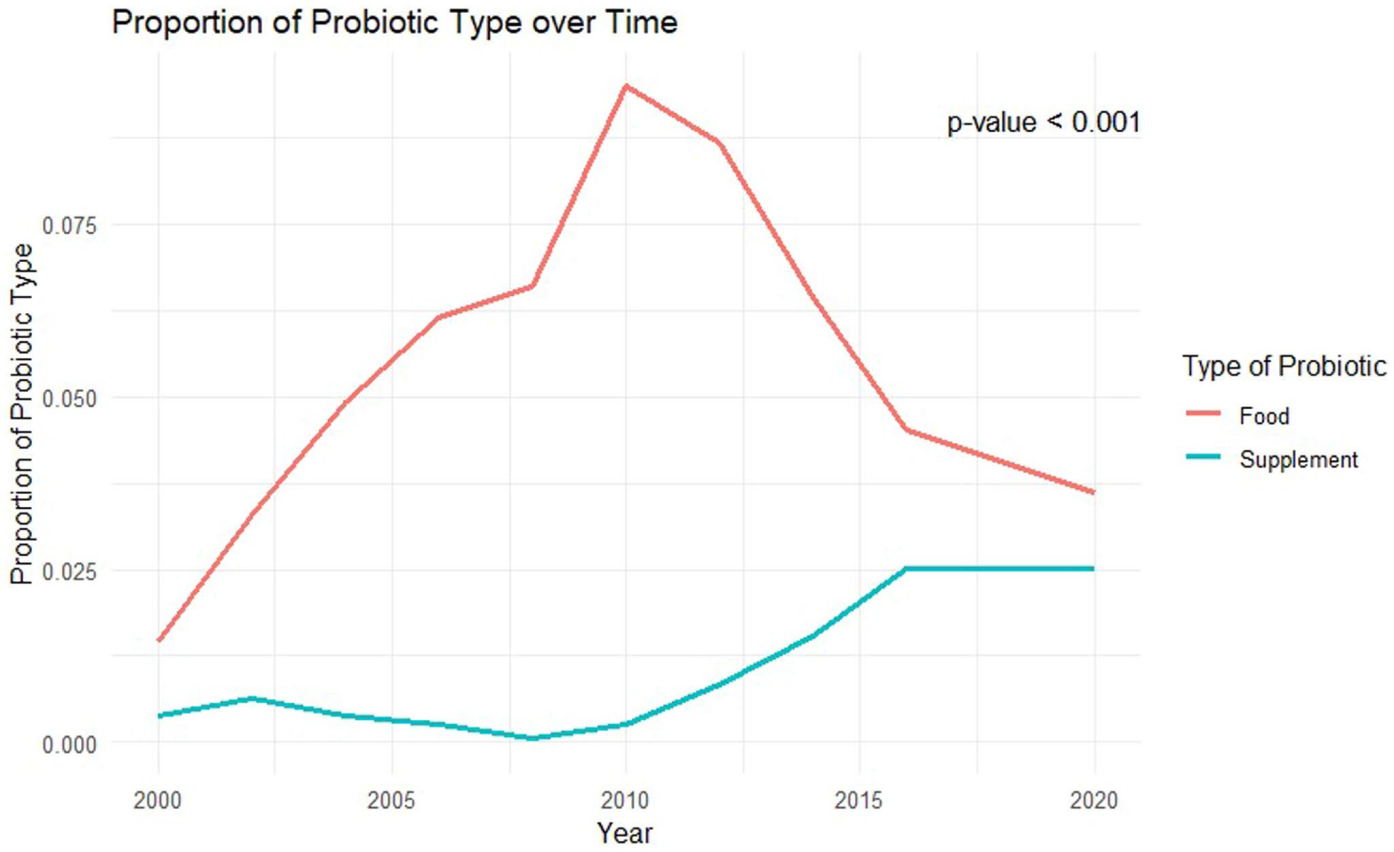
Average probiotic type used over time.

There were 14,992 participants who met cardiovascular disease criteria. The participant exclusion flowchart is included in Figure 1. After weighting, this sample represented 46,217,980 people in the USA. Weighted results showed there were 4,062,022 adults who were exposed to probiotic supplements or foods. Baseline characteristics of this population are included in Table 1. In terms of demographics, the probiotic exposure group was more likely to be female, non-Hispanic white, and to have higher income and education levels than the non-probiotic exposure group. In terms of medication use, the non-probiotic exposure group had a higher percentage of participants use dihydropyridine calcium-channel blockers (20.3% vs. 25.5%, *p* = 0.01), diuretics (12.1% vs. 15.5%, *p* = 0.02), and nitrates (1.3% vs. 3%, *p* = 0.01). The proportion of participants who used probiotics in general varied by year as shown in Figure 2. The trend of probiotic use over time was found to be significantly different from the trend of no probiotic use over time with a *p*-value <0.001. Figure 3 shows that the trend in the proportion of participants who used each type of probiotic also differed by year with a trend difference *p*-value <0.001.

**Table 1:** Comparison of subjects with and without probiotic use (*n* = 14,992; *N* = 46,217,980).

	Total (*N* = 46,217,980)	Probiotic use: yes (*n* = 4,062,022)	Probiotic use: no (*n* = 42,155,958)	*p*-value
Outcomes				
A1c (%)	6.2 ± 1.4	6.1 ± 1.1	6.2 ± 1.4	**<0.001**
HDL-C (mg/dL)	50 ± 15.5	53 ± 15.4	50 ± 15.4	**<0.001**
LDL-C (mg/dL)	112 ± 39	109 ± 38	113 ± 39	0.16
Total cholesterol (mg/dL)	196 ± 48	194 ± 50	196 ± 48	0.34
Triglyceride level (mg/dL)	160 ± 138	142 ± 88	162 ± 142	**<0.001**
ASCVD risk score (%)	19 ± 16	17 ± 15	19 ± 16	**0.01**
Blood pressure (systolic) (mmHg)	131 ± 20	130 ± 19	131 ± 20	0.06
Blood pressure (diastolic) (mmHg)	71 ± 13	70 ± 13	72 ± 13	**0.02**
BMI (kg/m^2^)	31.1 ± 7.0	30.8 ± 7.0	31.2 ± 7.0	0.26
Demographics				
Age (years)	60.7 ± 13.7	61.9 ± 12.7	60.5 ± 13.8	**0.01**
Sex				**< 0.001**
Male	50.2%	38.6%	51.3%	
Female	49.8%	61.4%	48.7%	
Race/Ethnicity				**< 0.001**
NHW	71.6%	79.4%	70.8%	
NHB	12.4%	7.4%	12.9%	
NHO	6.3%	6.2%	6.3%	
Hispanic	9.7%	7.0%	10.0%	
Marital status				0.93
Married/Partner	63.5%	63.7%	63.5%	
Not married	36.5%	36.3%	36.5%	
Ratio of family income to poverty	2.89 ± 1.61	3.28 ± 1.59	2.85 ± 1.60	**< 0.001**
Education level				**< 0.001**
< HS	21.4%	12.1%	22.3%	
HS	26.4%	21.2%	26.9%	
College+	52.3%	66.8%	50.9%	
MET score	840.15 ± 1,044.67	828.55 ± 1,017.01	841.47 ± 1,047.85	0.81
MET score (Categorical)				0.52
Low	41.9%	39.2%	42.2%	
Medium	32.2%	34.2%	32.0%	
High	25.9%	26.5%	25.8%	
Medications				
ACE inhibitor (yes)	43.0%	39.7%	43.4%	0.12
Aldosterone antagonist (yes)	2.2%	2.8%	2.2%	0.35
Antiplatelet (yes)	2.6%	1.8%	2.7%	0.09
ARBs (yes)	22.6%	25.8%	22.3%	0.12
Beta blockers (yes)	12.7%	12.0%	12.8%	0.60
DHP CCBs (yes)	25.0%	20.3%	25.5%	**0.01**
Digitalis (yes)	2.8%	2.1%	2.9%	0.21
Diuretics (yes)	15.2%	12.1%	15.5%	**0.02**
DOAC (yes)	0.3%	0.2%	0.4%	0.28
Ezetemibe (yes)	1.8%	2.4%	1.7%	0.28
Loop Diuretic (yes)	11.7%	9.8%	11.9%	0.11
nDHP CCBs (yes)	5.4%	5.4%	5.4%	0.98
Niacin (yes)	1.4%	2.4%	1.3%	0.07
Nitrate (yes)	2.9%	1.3%	3.0%	**0.01**
P2Y12 Inhibitor (yes)	7.2%	6.4%	7.3%	0.49
Statin (yes)	62.9%	63.9%	62.8%	0.57
Thiazide (yes)	28.6%	29.4%	28.6%	0.72
Vasodilator (yes)	1.5%	1.4%	1.5%	0.88

HDL-C, High-density lipoprotein cholesterol; LDL-C, Low-density lipoprotein cholesterol; ASCVD, Atherosclerotic cardiovascular disease; BMI, Body mass index; MET, Metabolic equivalent task; ACE, Angiotensin converting enzyme; ARB, Angiotensin receptor blocker; DHP, Dihydropyridine; CCB, Calcium channel blocker; DOAC, Direct oral anticoagulant; nDHP, non-Dihydropyridine. Bold formatting indicates *p*-values of statistical significance.

### Cardiovascular parameters

Cardiovascular parameters that were significantly different between probiotic exposure and non-probiotic exposure groups included A1C (6.1% ± 1.1 vs. 6.2% ± 1.4, *p* < 0.001), HDL-C (53 ± 15 vs. 50 ± 15 md/dL, *p* < 0.001), triglycerides (142 ± 88 vs. 162 ± 142, *p* < 0.001), ASCVD risk score (17% ± 15 vs. 19% ± 0.16, *p* = 0.01), and diastolic blood pressure (70 ± 13 vs. 72 ± 13, *p* = 0.02). Table 2 summarizes the unadjusted linear regression models for cardiometabolic parameters by probiotic use. Probiotic use was associated with higher HDL-C, and lower A1C, triglycerides, ASCVD risk score, and diastolic blood pressure. Tables 3–5 and Supplementary Tables 1, 2 summarize the adjusted linear regression models for A1c, HDL-C, Triglyceride level, ASCVD risk score, and diastolic blood pressure based on probiotic exposure. After multivariable adjustment, probiotic exposure was significantly associated with lower A1C (b −0.15; 95%CI: −0.24, −0.01), triglycerides (b −20.64; 95%CI: −34.86, −6.43), and ASCVD risk score (b −0.02; 95%CI: −0.04, −0.01). Probiotic exposure was also associated with higher HDL-C (b 1.60; 95%CI: 0.11, 3.08) in the adjusted models.

**Table 2:** Unadjusted linear regression models for cm outcomes by probiotic use (Yes).

Outcome	Estimate	95% CI	*p*-value
A1c (%)	−0.18	(−0.27, −0.08)	**< 0.001**
HDL-C (mg/dL)	3.16	(1.98, 4.34)	**< 0.001**
LDL-C (mg/dL)	−3.39	(−8.10, 1.32)	0.16
Total Cholesterol (mg/dL)	−1.98	(−6.07, 2.11)	0.34
Triglyceride Level (mg/dL)	−20.40	(−31.35, −9.45)	**<0.001**
ASCVD Risk Score (%)	−0.02	(−0.04, −0.01)	**0.01**
Blood Pressure (systolic) (mmHg)	−1.41	(−2.84, 0.03)	0.06
Blood Pressure (diastolic) (mmHg)	−1.50	(−2.73, −0.28)	**0.02**
BMI (kg/m^2^)	−0.32	(−0.88, 0.24)	0.26

HDL-C, High-density lipoprotein cholesterol; LDL-C, Low-density lipoprotein cholesterol; ASCVD, Atherosclerotic cardiovascular disease; BMI, Body mass index. Bold formatting indicates *p*-values of statistical significance.

**Table 3:** Adjusted linear regression model for outcome: A1c.

Variable	Estimate	95% CI	*p*-value
Probiotic use			
No (ref)	-	-	-
Yes	−0.15	(−0.24, −0.01)	**0.002**
Age (years)	0.003	(−0.004, 0.001)	0.10
Sex			
Female (ref)	-	-	-
Male	0.11	(0.03, 0.17)	**0.003**
Race/Ethnicity			
NHW (ref)	-	-	-
NHB	0.53	(0.41, 0.65)	**<0.001**
NHO	0.31	(0.16, 0.47)	**<0.001**
Hispanic	0.66	(0.50, 0.82)	**<0.001**
Marital status			
Married/Partner (ref)	−0.02	-	0.65
Not Married		(−0.11, 0.07)	
Ratio of Family income to poverty	−0.05	(−0.08, −0.03)	**< 0.001**
Education Level			
< HS (ref)	-	-	-
HS	−0.09	(−0.22, 0.04)	0.16
College+	−0.17	(−0.29, −0.06)	**0.003**
MET Score			
Low (ref)	-		
Medium	−0.03	(−0.11, 0.06)	0.52
High	−0.08	(−0.18, 0.02)	0.10

NHW, Non-Hispanic White; NHB, Non-Hispanic Black; NHO, Non-Hispanic Other; HS, Highschool; MET, Metabolic equivalent task. Bold formatting indicates *p*-values of statistical significance.

**Table 4:** Adjusted linear regression model for outcome: HDL-C.

Variable	Estimate	95% CI	*p*-value
Probiotic use			
No (ref)	-	-	-
Yes	1.60	(0.11, 3.08)	**0.04**
Age (years)	0.21	(0.18, 0.24)	**<0.001**
Sex			
Female (ref)	-	-	-
Male	−10.61	(−11.62, −9.60)	**<0.001**
Race/Ethnicity			
NHW (ref)	-	-	-
NHB	4.73	(3.65, 5.81)	**<0.001**
NHO	−0.06	(−1.51, 1.40)	0.94
Hispanic	0.31	(−0.80, 1.41)	0.58
Marital status			
Married/Partner (ref)	-	-	-
Not Married	1.04	(0.18, 1.91)	**0.02**
Ratio of family income to poverty	0.87	(0.55, 1.19)	**< 0.001**
Education level			
< HS (ref)	-	-	-
HS	0.74	(−0.86, 2.33)	0.36
College+	1.90	(0.45, 3.35)	**0.01**
MET score			
Low (ref)	-	-	
Medium	0.28	(−0.71, 1.27)	0.57
High	2.17	(1.08, 3.27)	**< 0.001**

NHW, Non-Hispanic White; NHB, Non-Hispanic Black; NHO, Non-Hispanic Other; HS, Highschool; MET, Metabolic equivalent task. Bold formatting indicates *p*-values of statistical significance.

**Table 5:** Adjusted linear regression model for outcome: triglyceride level.

Variable	Estimate	95% CI	*p*-value
Probiotic use			
No (ref)	-	-	-
Yes	−20.64	(−34.86, −6.43)	**0.005**
Age (years)	−1.38	(−1.81, −0.94)	**<0.001**
Sex			
Female (ref)	-	-	-
Male	19.67	(8.05, 31.28)	**0.001**
Race/Ethnicity			
NHW (ref)	-	-	-
NHB	−51.07	(−64.30, −37.83)	**<0.001**
NHO	5.52	(−25.25, 36.30)	0.72
Hispanic	1.25	(−13.99, 16.50)	0.87
Marital status			
Married/Partner (ref)	-	-	0.59
Not married	−3.37	(−15.74, 8.99)	
Ratio of family income to poverty	−3.14	(−7.06, 0.77)	0.11
Education level			
< HS (ref)	-	-	-
HS	4.13	(−13.88, 22.14)	0.65
College+	−2.81	(−18.98, 13.35)	0.73
MET score			
Low (ref)	-	-	
Medium	−2.09	(−16.39, 12.21)	0.77
High	−14.54	(−29.28, 0.20)	0.053

NHW, Non-Hispanic White; NHB, Non-Hispanic Black; NHO, Non-Hispanic Other; HS, Highschool; MET, Metabolic equivalent task. Bold formatting indicates *p*-values of statistical significance.

### Associations of CV risk factors by probiotic type

Within the probiotic exposure group, a weighted population of 763,288 were exposed to probiotic supplements and a weighted population of 3,179,008 were exposed to probiotic foods including yogurt. The baseline characteristics of probiotic supplement exposure and probiotic food participants is presented in Supplementary Table 3. Cardiometabolic parameters that differed between probiotic supplements and probiotic foods included LDL-C (95 ± 36 vs. 112 ± 37, *p* = 0.003) and total cholesterol (185 ± 46 vs. 196 ± 51, *p* = 0.047). The unadjusted linear regression models for cardiometabolic outcomes by probiotic type found in Supplementary Table 4 shows a negative association between probiotic supplement use and both LDL-C and total cholesterol levels, with adults who used supplements having significantly lower LDL-C (b −17.07; 95%CI: −28.29, −5.85) and significantly lower total cholesterol (b −10.61; 95%CI: −23.78, −0.14) compared to those who consumed probiotic foods. The probiotic supplement participants were found to be older than the probiotic food/yogurt participants (65 ± 11 vs. 61 ± 13, *p* < 0.001). Supplementary Tables 5, 6 summarize the adjusted linear regression models for LDL-C and total cholesterol based on probiotic type exposure. After multi-variable adjustment, probiotic supplement use remained associated with lower LDL-C (b −27.84; 95%CI: −41.26, −14.41) and total cholesterol (b −16.76; 95%CI: −27.88, −5.63).

Within the probiotic supplement group, there were multiple different strains of bacteria used. The most common bacteria included multiple species within the geni *Lactobacillus* and *Bifidobacterium*. Other strains included *Bacillus coagulans*, *Streptococcus salivarius*, etc. There were 486 unique probiotic supplement brands that were ingested by the probiotic use group. There were 134 brands that consisted solely of *Lactobacillus*, 235 brands that included a combination of *Lactobacillus* and *Bifidobacterium*, and 110 brands that included just *Bifidobacterium* or just other bacteria. Table 6 shows the comparison of demographic and cardiovascular risk parameters between these groups. There were no significant demographic differences between probiotic supplement groups aside from the *Bifidobacterium*/other group was less likely to be non-Hispanic white than the *Lactobacillus* group. In terms of cardiovascular risk profiles, after initial analysis, the *Bifidobacterium*/other group was associated with lower HDL-C and diastolic blood pressure than the *Lactobacillus* group. However, after adjusted linear regression, as shown in Table 7, only the diastolic blood pressure was significantly lower in the *Bifidobacterium*/other group compared to the *Lactobacillus* group.

**Table 6:** Comparison of subjects by probiotic supplement type (*n* = 169; *N* = 763,288).

	Lact (*N* = 307,565)	Combo (*N* = 337,775)	Other/Bifi (*N* = 117,949)	*p*-value
Outcomes				
A1c (%)	6.0 ± 0.9	5.8 ± 0.7	6.1 ± 0.9	0.38
HDL-C (mg/dL)	55 ± 19	57 ± 17	48 ± 9	**0.01**
LDL-C (mg/dL)	99 ± 46	94 ± 31	90 ± 25	0.78
Total cholesterol (mg/dL)	190 ± 55	183 ± 36	178 ± 43	0.74
Triglyceride level (mg/dL)	122 ± 57	133 ± 86	137 ± 57	0.60
ASCVD risk score (%)	18 ± 15	17 ± 15	21 ± 15	0.89
Blood pressure (systolic) (mmHg)	130 ± 21	133 ± 20	135 ± 24	0.88
Blood pressure (diastolic) (mmHg)	70 ± 10	73 ± 12	61 ± 14	**0.01**
BMI (kg/m^2^)	31 ± 8	32 ± 7	31 ± 8	0.94
Demographics				
Age (years)	63.9 ± 12	66.7 ± 9.3	63.6 ± 14.5	0.24
Sex				0.06
Male	35.4%	35.0%	68.1%	
Female	64.6%	65.0%	31.9%	
Race/Ethnicity				**0.01**
NHW	89.0%	83.4%	61.7%	
NHB	6.7%	6.0%	10.6%	
NHO	2.3%	1.6%	15.1%	
Hispanic	2.1%	9.0%	12.5%	
Marital status				0.80
Married/Partner	67.3%	61.9%	69.3%	
Not married	32.7%	38.1%	30.7%	
Ratio of family income to poverty	3.2 ± 1.5	3.8 ± 1.49	2.7 ± 1.5	0.07
Education level				0.45
< HS	12.5%	7.1%	3.9%	
HS	21.6%	15.6%	10.1%	
College+	65.8%	77.3%	86.1%	
MET score	839.6 ± 756.7	787.3 ± 1,009.0	804.6 ± 958.2	0.95
MET score (Categorical)				0.41
Low	33.2%	30.5%	35.1%	
Medium	33.6%	52.2%	26.6%	
High	33.1%	17.2%	38.3%	

HDL-C, High-density lipoprotein cholesterol; LDL-C, Low-density lipoprotein cholesterol; ASCVD, Atherosclerotic cardiovascular disease; BMI, Body mass index; MET, Metabolic equivalent task. Bold formatting indicates *p*-values of statistical significance.

**Table 7:** Unadjusted linear regression models for CM outcomes by probiotic supplement type.

Outcome	Probiotic supplement	Estimate	95% CI	*p*-value
HDL-C	*Lactobacillus (ref)*	-	-	-
	*Lactobacillus/Bifidobacterium*	2.4	(−7.34, 12.31)	0.61
	*Bifidobacterium/o*ther	−6.71	(−14.94, 1.53)	0.11
Blood pressure (diastolic)	*Lactobacillus (ref)*	-	-	-
	*Lactobacillus/Bifidobacterium*	3.00	(−2.06, 8.06)	0.23
	*Bifidobacterium/o*ther	−10.49	(−18.30, −2.68)	**0.01**

HDL-C, High-density lipoprotein cholesterol. Bold formatting indicates *p*-values of statistical significance.

## Discussion

### Comparisons between probiotic vs. non-probiotic groups

Analyses of NHANES data suggest that probiotic ingestion is associated with improved cardiometabolic parameters in patients with existing ASCVD or two or more traditional cardiovascular risk factors. Specifically, probiotic use was associated with a 1.6% decrease in hemoglobin a1c, 12.3% decrease in triglycerides, 10.5% decrease in ASCVD risk score, a 2.8% decrease in diastolic blood pressure, and a 6% increase in HDL-C. These differences were present after controlling for demographics and other possible confounders including medication use and exercise activity. While some of these differences are modest despite being statistically significant, they show that gut microbiome modulation with the use of probiotics is associated with a positive impact on multiple pathways involved in the pathogenesis of ASCVD.

In our study, the observed lower ASCVD risk score in those taking probiotics suggests probiotic ingestion is likely associated with fewer cardiovascular events in the next ten years. These data are consistent with a prior study showing that two-unit improvement resulted in ASCVD risk score results in an additional 0.9 life-years with ASCVD (15, 16). Taken together, our data suggest there is merit in testing the provocative and biologically plausible hypothesis that probiotic supplementation will reduce cardiovascular risk in conjunction with current guideline-directed medical therapy.

Our results have some differences from prior studies that analyzed the effects of probiotics on metabolic diseases. Lau et al. similarly used NHANES data to study the association of probiotic supplementation and metabolic-related disorders (8). They included 38,802 adults in their study and found that probiotic supplementation was associated with lower prevalence of obesity and hypertension along with decreases in BMI, systolic blood pressure and diastolic blood pressure along with an increase in HDL-C. Notably, Lau et al. did not find significant differences in hemoglobin A1c or total cholesterol between test groups as we did (8). These differences may be partially explained by the differences in the baseline characteristics of our sample populations. Further, we used NHANES’ built-in weighting parameters to increase the power of our study, allowing us to detect more modest differences in cardiovascular health measures.

Notably, our finding that probiotic use was associated with lower hemoglobin A1c. This finding is consistent with a meta-analysis of randomized controlled trials conducted by Ruan et al. in 2015, which suggested that probiotic ingestion may improve glycemic control, particularly in those with high fasting blood glucose (17).

Previous cross-sectional studies have shown an association between ingestion of probiotic supplements and foods and a lower prevalence of obesity and hypertension in the general population but did not specifically focus on individuals with ASCVD or multiple cardiac risk factors who are highest risk for adverse cardiovascular events (2, 3, 7). There have been meta-analyses of small randomized-control trials, which included participants with ASCVD or multiple cardiac risk factors, that have shown similar results including reduced levels of LDL-C cholesterol, glucose, and increased levels HDL-C (18–22). However, these analyses are based on small, selected subject populations and are difficult to generalize to a population level (18–22). To our knowledge, our study is the first one to determine associations between probiotic supplementation on CAD risk factors specifically in a group with existing ASCVD or multiple ASCVD risk factors generalizable to a population level.

We found that exposure to probiotic supplementation or probiotic foods was associated with a lower A1C percentage, lower circulating triglycerides, and higher HDL-C levels. Consistent with these findings, probiotic use was associated with a lower ASCVD risk score. While mechanistic conclusions cannot be discerned from our data, prior mechanistic work does suggest that probiotic supplementation can reduce systemic inflammation, reduce cholesterol levels, and improve endothelial function (3). A trial of 15 men with stable CAD demonstrated that six weeks of supplementation with twenty billion CFUs of *Lactobacillus plantarum* induced favorable metabolic effects including downregulation of inflammation driven by IL-1β, TNF-a, and upregulation of regulatory T-cells (3). These mechanisms are likely contributing to the improved cardiovascular profile in our patients and further studies should be conducted to determine the exact pathways of action.

At baseline, the non-probiotic group was more likely to be prescribed non-dihydropyridine calcium channel blockers, diuretics, and nitrates for their ASCVD and disease sequelae. The reasons for greater use of these medication in the non-probiotic-using population is unclear. This could be due to differences in baseline characteristics of these populations. It is also possible that probiotic use could be reducing the need for these medications which are commonly used for symptomatic relief from heart failure or angina. Further research into the potential mechanisms of effect of probiotics are warranted to discern probiotic biological effects that might have favorable effects on volume status and vascular calcification burden in heart failure, angina, and hypertension.

### Comparisons between probiotic ingestion types

Our data suggest that individuals who ingest their probiotic in the form of a supplement rather than as a food product were more likely to have lower LDL-C and triglyceride levels. While this observation could be secondary to unmeasured differences in the two studies’ populations, most of the probiotic supplements in our study contained above ten billion colony forming units (CFUs) whereas most yogurts and many of the probiotic foods have much lower concentrations of probiotic (commonly around one million CFUs) (23). Therefore, it is possible that those who take supplements are more likely to be colonized by a greater concentration of probiotic and realize biological effects.

Figures 2, 3 showed that the rates of probiotic use have varied over the past two decades. Interestingly, the rates of use have not been steadily increasing but rather peaked in 2010 at 10% of the population and have remained below that level since. The use of probiotic foods has accounted for most of the probiotic use throughout the study period and the use trend follows a similar yearly pattern to that of probiotic use in general. Probiotic supplement use has increased in recent years but has plateaued at 2.5% of the population since 2016. The cause for these differences and the yearly trends is uncertain but could be impacted by a mix of cultural, social, and economic trends. For example, consumers preferring frozen yogurt to ice cream because of the proposed health benefits has contributed to the North American frozen yogurt market size to increase to $511 million in 2023, which is projected to continue growing (24). If clinicians aim to increase the use of probiotics, further knowledge regarding these trends may be vital to their marketing and adoption approaches.

Little research has been conducted comparing the impact of probiotic supplements versus probiotic foods on cardiovascular risk factors. Prior studies that have been completed have found that food and supplements have similar impacts (8, 25). Our study shows that probiotic supplementation is associated with a significantly lower LDL-C and total cholesterol levels compared to ingestion of probiotic as a food product. Differences between our findings and those of these prior studies could be due to differences in the populations studied. Our patients have existing or are high risk for ASCVD and so the impact of supplements may show a greater benefit than in healthy, general populations. This data may also indicate that individuals who take supplements may be more health conscious or may take higher, more concentrated doses administered with a greater influence on LDL-C and total cholesterol levels. As stated previously, the large power of our study may also reveal significant findings that previous studies were not able to show. Additional interventional studies will be necessary to better delineate the reasons behind this unique finding.

Within the probiotic supplement group, our data suggests that diastolic blood pressure was the only significant difference between solely *Lactobacillus* brands and *Bifidobacterium*/other strain brands. HDL-C was higher in the *Lactobacillus* and *Lactobacillus*/*Bifidobacterium* combination groups than in the *Bifidobacterium*/other group, however these differences were not statistically significant. The *Lactobacillus* group and the *Lactobacillus*/*Bifidobacterium* combination group did not have any significant statistical differences in cardiovascular risk profiles. There has been limited research comparing the efficacy of specific probiotic strains, however, the most promising probiotic strains are generally considered to be members of the genera: *Lactobacillus*, *Bifidobacterium* and *Enterococcus* (26). Comparing individual strains for efficacy in reducing metabolic disease and ASCVD should be a topic for further study to determine if there is a clinical benefit to selecting one strain over another in patients with ASCVD.

Our study has some limitations. Like all cross-sectional studies, we cannot infer causality from our results. Nevertheless, the data generate multiple potential testable hypotheses about the impact of probiotic supplementation on CV risk factors in patients with ASCVD to further determine if our associations may have causal foundations. Much of the NHANES database, including dietary history, is self-reported. To mitigate inaccuracies, NHANES only provides survey information that is deemed reliable, however this remains a limitation (27). Another limitation is that this study did not evaluate the impact of health-promoting behaviors or biases aside from those listed above. Other biases that could have impacted our cohorts’ cardiovascular parameters include but are not limited to differences in prescribed medication adherence, health benefits of individual diets, and sleep hygiene. Additionally, the duration, frequency, and dose of probiotic exposure were not considered due to the confines of the database, which is an important limitation when translating these findings to clinical practice. The supplements we studied also varied in their non-probiotic ingredients. Some of the supplements included prebiotics along with other vitamins and minerals which may have contributed to varying outcomes. However, the NHANES data was not granular enough to parse out these differences in ingredients and control for them. Previous studies have assessed dairy product intake and associations with metabolic syndrome using NHANES data, but our study is the first to assess dairy product intake along with probiotic supplementation in patients with existing cardiovascular disease (28). Another strength of our dataset is that we were able to account for demographic differences in patients, particularly race. Racial and geographical differences have been found to be associated with variations in microbiome throughout the human body (29). Our analysis controlled for multiple racial and ethnic groups, as reported by respondents, and determined that these beneficial aspects of probiotic ingestion were still significant. Interindividual differences in gut microbiome can impact responses to dietary and probiotic supplementation intervention (30). While probiotic supplementation did result in a positive response in a large portion of our sample, our study did not include individual gut microbiome samples in our analysis to determine whether non-responders had specific microbiome make-ups that impaired their probiotic response. Balanced against these limitations are strengths including a large cross-sectional, a large, validated dataset that was weighted to represent the US population, and the novelty of our findings in this population. Another strength of our study is our analysis of probiotic ingestion type and probiotic strain, which has previously been limited in the current literature. Further controlled trials should be conducted that will account for probiotic consumption frequency, dose, and quantity along with individual gut microbiome data, to strengthen the causative association between probiotic ingestion and improvement in cardiovascular risk profiles.

## Conclusion

Our study reinforces the beneficial association between probiotic ingestion and cardiovascular health, particularly in patients with existing atherosclerotic disease or multiple CV risk factors. Probiotic ingestion was associated with higher HDL-C levels and lower A1c levels, lower triglyceride levels and lower ASCVD risk scores. Our study found that probiotic supplements were more associated with lower LDL-C and total cholesterol levels than probiotic foods. Our study supports further testing probiotic supplementation, both type and route of administration, as a method for reduce CV risk in high-risk patients.

## Data Availability

Publicly available datasets were analyzed in this study. This data can be found at: https://wwwn.cdc.gov/nchs/nhanes/ (NHANES).
