# Supplementary material for: Association of probiotic supplementation and cardiovascular risk profiles of patients with coronary artery disease—a cross-sectional analysis of the NHANES database between 1999–2019
Source: Front Nutr. 2025 Feb 28;12:1495633. doi: 10.3389/fnut.2025.1495633 (PMC11906339; doi:10.3389/fnut.2025.1495633)
Supplement: Supplementary file 1 [file Table_1.docx]

**SUPPLEMENTARY TABLES**

**Supplementary Table 1**: Adjusted Linear Regression Model for Outcome: ASCVD Score

| **Variable** | **Estimate** | **95% CI** | **p-value** |
| --- | --- | --- | --- |
| Probiotic Use  No (ref)  Yes | -  -0.02 | -  (-0.04, -0.01) | -  **<0.001** |
| Age | 0.01 | (0.01, 0.01) | **<0.001** |
| Sex  Female (ref)  Male | -  0.06 | -  (0.05, 0.07) | -  **<0.001** |
| Race/Ethnicity  NHW (ref)  NHB  NHO  Hispanic | -  0.03  0.005  0.005 | -  (0.02, 0.04)  (-0.01, 0.02)  (-0.01, 0.02) | -  **<0.001**  0.56  0.46 |
| Marital Status  Married/Partner (ref)  Not Married | -  0.01 | -  (0.0004, 0.02) | -  **0.04** |
| Ratio of Family income to poverty | -0.01 | (-0.01, -0.01) | **<0.001** |
| Education Level  < HS (ref)  HS  College+ | -  -0.02  -0.02 | -  (-0.03, -0.003)  (-0.03, -0.01) | -  **0.02**  **0.004** |
| MET Score  Low (ref)  Medium  High | -  0.003  -0.01 | -  (-0.01, 0.01)  (-0.02, 0.0003) | 0.50  0.06 |

**Supplementary Table 2**: Adjusted Linear Regression Model for Outcome: Blood Pressure (diastolic)

| **Variable** | **Estimate** | **95% CI** | **p-value** |
| --- | --- | --- | --- |
| Probiotic Use  No (ref)  Yes | -  -1.12 | -  (-2.67, 0.44) | -  0.16 |
| Age | -0.30 | (-0.33, -0.27) | **<0.001** |
| Sex  Female (ref)  Male | -  1.96 | -  (1.18, 2.74) | -  **<0.001** |
| Race/Ethnicity  NHW (ref)  NHB  NHO  Hispanic | -  2.79  1.36  0.43 | -  (1.76, 3.82)  (-0.12, 2.83)  (-0.76, 1.61) | -  **<0.001**  0.07  0.48 |
| Marital Status  Married/Partner (ref)  Not Married | -  0.55 | -  (-0.30, 1.41) | -  0.20 |
| Ratio of Family income to poverty | 0.39 | (0.13, 0.65) | **0.003** |
| Education Level  < HS (ref)  HS  College+ | -  0.75  -0.10 | -  (-0.61, 2.12)  (-1.15, 0.94) | -  0.28  0.85 |
| MET Score  Low (ref)  Medium  High | -  0.57  -0.06 | -  (-0.42, 1.57)  (-1.04, 0.92) | 0.26  0.91 |

**Supplemental Table 3**: Comparison of Subjects by Probiotic Type^1^ (n = 1,000; N = 3,942,297)

|  | **Total**  **(N = 3,942,297)** | **Probiotic Supplement**  **(N = 763,288)** | **Probiotic Food/Yogurt**  **(N = 3,179,008)** | **p-value** |
| --- | --- | --- | --- | --- |
| **Outcomes** | | | | |
| A1c (%) | 6.0 ± 1.1 | 6.0 ± 0.8 | 6.1 ± 1.2 | 0.29 |
| HDL (mg/dL) | 53 ± 15 | 55 ± 17 | 53 ± 15 | 0.23 |
| LDL (mg/dL) | 109 ± 38 | 95 ± 36 | 112 ± 37 | **0.003** |
| Total Cholesterol (mg/dL) | 194 ± 50 | 185 ± 46 | 196 ± 51 | **0.047** |
| Triglyceride Level (mg/dL) | 140 ± 81 | 129 ± 71 | 142 ± 83 | 0.34 |
| ASCVD Risk Score (%) | 17 ± 15 | 18 ± 15 | 16 ± 15 | 0.36 |
| Blood Pressure (systolic) (mmHg) | 130 ± 19 | 132 ± 21 | 129 ± 19 | 0.14 |
| Blood Pressure (diastolic) (mmHg) | 70 ± 13 | 70 ± 12 | 70 ± 13 | 0.79 |
| BMI (kg/m^2^) | 30.9 ± 7.0 | 31.4 ± 7.5 | 30.7 ± 6.9 | 0.46 |
| **Demographics** | | | | |
| Age (years) | 61.9 ± 12.7 | 65.1 ± 11.4 | 61.1 ± 12.8 | **<0.001** |
| Sex  Male  Female | 39.5%  60.5% | 38.8%  61.2% | 39.7%  60.3% | 0.85 |
| Race/Ethnicity  NHW  NHB  NHO  Hispanic | 79.0%  7.6%  6.3%  7.2% | 81.9%  7.2%  4.3%  6.6% | 78.3%  7.7%  6.7%  7.3% | 0.64 |
| Marital Status  Married/Partner  Not Married | 64.0%  36.0% | 65.2%  34.8% | 63.8%  36.2% | 0.81 |
| Ratio of Family income to poverty | 3.29 ± 1.58 | 3.39 ± 1.54 | 3.27 ± 1.58 | 0.47 |
| Education Level  < HS  HS  College+ | 12.2%  21.6%  66.1% | 9.0%  17.7%  73.4% | 13.0%  22.6%  64.4% | 0.18 |
| MET Score | 839.36 ± 1,027.94 | 809.32 ± 907.24 | 846.18 ± 1,054.12 | 0.74 |
| MET Score (Categorical)  Low  Medium High | 38.8%  34.4%  26.9% | 33.2%  41.5%  25.3% | 40.0%  32.7%  27.2% | 0.39 |

^1^ those with both supplement and food removed.

**Supplemental Table 4**: Unadjusted Linear Regression Models for CM Outcomes by Probiotic Type (Supplement)

| **Outcome** | **Estimate** | **95% CI** | **p-value** |
| --- | --- | --- | --- |
| A1c (%) | -0.09 | (-0.27, 0.08) | 0.29 |
| HDL (mg/dL) | 2.17 | (-1.39, 5.73) | 0.23 |
| LDL (mg/dL) | -17.07 | (-28.29, -5.85) | **0.003** |
| Total Cholesterol (mg/dL) | -10.61 | (-23.78, -0.14) | **0.047** |
| Triglyceride Level (mg/dL) | -13.07 | (-40.31, 14.16) | 0.34 |
| ASCVD Risk Score (%) | 0.02 | (-0.02, 0.06) | 0.36 |
| Blood Pressure (systolic) | 3.15 | (-1.04, 7.35) | 0.14 |
| Blood Pressure (diastolic) | 0.37 | (-2.39, 3.12) | 0.79 |
| BMI | 0.71 | (-1.19, 2.60) | 0.46 |

**Supplemental Table 5**: Adjusted Linear Regression Model for Outcome: LDL

| **Variable** | **Estimate** | **95% CI** | **p-value** |
| --- | --- | --- | --- |
| Probiotic Type  Food/Yogurt (ref)  Supplement | -  -27.84 | -  (-41.26, -14.41) | -  **<0.001** |
| Age (years) | -0.34 | (-0.77, 0.10) | 0.13 |
| Sex  Female (ref)  Male | -  -8.61 | -  (-23.32, 6.11) | -  0.24 |
| Race/Ethnicity  NHW (ref)  NHB  NHO  Hispanic | -  7.42  -14.98  -6.40 | -  (-12.31, 27.16)  (-27.87, -2.08)  (-22.59, 9.80) | -  0.45  **0.02**  0.43 |
| Marital Status  Married/Partner (ref)  Not Married | -  4.43 | -  (-9.30, 18.16) | -  0.52 |
| Ratio of Family income to poverty | -0.95 | (-4.34, 2.45) | 0.58 |
| Education Level  < HS (ref)  HS  College+ | -  6.95  7.67 | -  (-12.87, 26.77)  (-7.95, 23.28) | -  0.48  0.33 |
| MET Score  Low (ref)  Medium  High | -  9.92  10.49 | -  (-3.16, 23.01)  (-3.10, 24.08) | 0.13  0.13 |

**Supplemental Table 6**: Adjusted Linear Regression Model for Outcome: Total Cholesterol

| **Variable** | **Estimate** | **95% CI** | **p-value** |
| --- | --- | --- | --- |
| Probiotic Type  Food/Yogurt (ref)  Supplement | -  -16.76 | -  (-27.88, -5.63) | -  **0.004** |
| Age (years) | -0.48 | (-0.84, -0.12) | **0.01** |
| Sex  Female (ref)  Male | -  -19.43 | -  (-29.65, -9.20) | -  **<0.001** |
| Race/Ethnicity  NHW (ref)  NHB  NHO  Hispanic | -  -2.53  -11.33  2.29 | -  (-16.09, 11.03)  (-24.73, 2.07)  (-9.00, 13.58) | -  0.71  0.10  0.69 |
| Marital Status  Married/Partner (ref)  Not Married | -  6.56 | -  (-4.15, 17.27) | -  0.23 |
| Ratio of Family income to poverty | -0.71 | (-3.47, 2.06) | 0.61 |
| Education Level  < HS (ref)  HS  College+ | -  7.53  11.33 | -  (-7.98, 23.05)  (-1.20, 23.87) | -  0.34  0.08 |
| MET Score  Low (ref)  Medium  High | -  9.24  6.18 | -  (-2.45, 20.94)  (-5.71, 18.07) | 0.12  0.30 |

Supplemental Table 7: List of Probiotic Foods

| **ID** | **LABEL** |
| --- | --- |
| **100148** | Sauerkraut, cooked, fat added in cooking W/ VEGETABLE OIL, NFS (INCLUDE OIL, NFS) |
| **204458** | Cabbage salad or coleslaw, with dressing W/ YOGURT DRESSING |
| **205057** | Sauerkraut, cooked, fat added in cooking W/ ANIMAL FAT OR MEAT DRIPPINGS |
| **206291** | Potato salad W/ NONFAT YOGURT |
| **206539** | Apple salad with dressing W/ YOGURT, NS AS TO TYPE OF MILK OR FLAVOR |
| **206632** | Fruit salad (excluding citrus fruits) with cream W/ YOGURT |
| **206756** | Tuna salad W/ PLAIN YOGURT, NONFAT MILK |
| **206770** | Sauerkraut, cooked, fat added in cooking W/ BUTTER, NFS |
| **206821** | Cucumber salad with creamy dressing W/ NONFAT YOGURT |
| **207450** | Apple salad with dressing W/ YOGURT DRESSING |
| **11115000** | BUTTERMILK, FLUID (INCLUDE KEFIR MILK) |
| **11115100** | BUTTERMILK, FLUID, 1% FAT |
| **11115200** | BUTTERMILK, FLUID, 2% FAT |
| **11115300** | BUTTERMILK, FLUID, WHOLE |
| **11115400** | KEFIR, NS AS TO FAT CONTENT |
| **11400000** | YOGURT, NFS |
| **11400010** | YOGURT, GREEK, NS AS TO TYPE OF MILK OR FLAVOR |
| **11410000** | YOGURT, NS AS TO TYPE OF MILK/FLAVOR |
| **11411010** | YOGURT, PLAIN, NS AS TO TYPE OF MILK |
| **11411100** | YOGURT, PLAIN, WHOLE MILK |
| **11411200** | YOGURT, PLAIN, LOWFAT MILK |
| **11411300** | YOGURT, PLAIN, NONFAT MILK |
| **11411390** | YOGURT, GREEK, NS AS TO TYPE OF MILK, PLAIN |
| **11411400** | YOGURT, GREEK, PLAIN, WHOLE MILK |
| **11411410** | YOGURT, GREEK, PLAIN, LOW FAT |
| **11411420** | YOGURT, GREEK, PLAIN, NONFAT MILK |
| **11420000** | YOGURT, VANILLA, LEMON, COFFEE, NS AS TO MILK TYPE |
| **11421000** | YOGURT, VANILLA, LEMON, COFFEE, WHOLE MILK |
| **11422000** | YOGURT, VANILLA, LEMON, COFFEE, LOWFAT MILK |
| **11422100** | YOGURT, VANILLA, LEMON, COFFEE, LOWFAT MILK, LOW CAL SWTNR |
| **11423000** | YOGURT, VANILLA, LEMON, COFFEE, NONFAT MILK |
| **11424000** | YOGURT, VANILLA, LEMON, COFFEE, NONFAT MILK, LOW CAL SWEET |
| **11424500** | YOGURT, GREEK, VANILLA, WHOLE MILK |
| **11424510** | YOGURT, GREEK, VANILLA, LOW FAT |
| **11424520** | YOGURT, GREEK, VANILLA, NONFAT |
| **11425000** | YOGURT, CHOCOLATE, NS AS TO TYPE OF MILK |
| **11426000** | YOGURT, CHOCOLATE, WHOLE MILK |
| **11427000** | YOGURT, CHOCOLATE, NONFAT MILK |
| **11428000** | YOGURT, GREEK, CHOCOLATE, NONFAT |
| **11430000** | YOGURT, FRUIT VARIETY, NS AS TO MILK TYPE |
| **11431000** | YOGURT, FRUIT VARIETY, WHOLE MILK |
| **11432000** | YOGURT, FRUIT VARIETY, LOWFAT MILK |
| **11432500** | YOGURT, FRUIT VARIETY, LOWFAT MILK, W/ LOW CAL SWEETENER |
| **11433000** | YOGURT, FRUIT VARIETY, NONFAT MILK |
| **11433500** | YOGURT, FRUITED, NONFAT MILK, LOW CAL SWEETENER |
| **11433990** | YOGURT, GREEK, NS AS TO TYPE OF MILK, FRUIT |
| **11434000** | YOGURT, GREEK, FRUIT, WHOLE MILK |
| **11434010** | YOGURT, GREEK, FRUIT, LOW FAT |
| **11434020** | YOGURT, GREEK, FRUIT, NONFAT |
| **11434090** | YOGURT, NS AS TO TYPE OF MILK, FLAVORS OTHER THAN FRUIT |
| **11434100** | YOGURT, WHOLE MILK, FLAVORS OTHER THAN FRUIT |
| **11434200** | YOGURT, LOW FAT MILK, FLAVORS OTHER THAN FRUIT |
| **11434300** | YOGURT, NONFAT MILK, FLAVORS OTHER THAN FRUIT |
| **11435000** | YOGURT, GREEK, NS AS TO TYPE OF MILK, FLAVORS OTHER THAN FRU |
| **11435010** | YOGURT, GREEK, WHOLE MILK, FLAVORS OTHER THAN FRUIT |
| **11435020** | YOGURT, GREEK, LOW FAT MILK, FLAVORS OTHER THAN FRUIT |
| **11435030** | YOGURT, GREEK, NONFAT MILK, FLAVORS OTHER THAN FRUIT |
| **11435100** | YOGURT, GREEK, WITH OATS |
| **11436000** | YOGURT, LIQUID |
| **11440010** | CHIPOTLE DIP, YOGURT BASED |
| **11440020** | DILL DIP, YOGURT BASED |
| **11440030** | ONION DIP, YOGURT BASED |
| **11440040** | RANCH DIP, YOGURT BASED |
| **11440050** | SPINACH DIP, YOGURT BASED |
| **11440070** | VEGETABLE DIP, YOGURT BASED |
| **11444000** | YOGURT, FRUIT & NUTS, NS AS TO TYPE OF MILK |
| **11445000** | YOGURT, FRUIT & NUTS, LOWFAT MILK |
| **11446000** | FRUIT AND LOWFAT YOGURT PARFAIT |
| **11459990** | YOGURT, FROZEN, NS AS TO FLAVOR, NS TO TYPE OF MILK |
| **11460000** | YOGURT, FROZEN, NOT CHOCOLATE, TYPE OF MILK NS |
| **11460100** | YOGURT, FROZEN, CHOCOLATE, TYPE OF MILK NS |
| **11460150** | YOGURT, FROZEN, NS AS TO FLAVOR, LOWFAT MILK |
| **11460160** | YOGURT, FROZEN, CHOCOLATE, LOWFAT MILK |
| **11460170** | YOGURT, FROZEN, NOT CHOCOLATE, LOWFAT MILK |
| **11460190** | YOGURT, FROZEN, NS AS TO FLAVOR, NONFAT MILK |
| **11460200** | YOGURT, FROZEN, CHOCOLATE, NONFAT MILK |
| **11460250** | YOGURT,FROZEN,NOT CHOCOLATE,W/ SORBET/SORBET-COATED |
| **11460300** | YOGURT, FROZEN, NOT CHOCOLATE, NONFAT MILK |
| **11460400** | YOGURT,FRZ,CHOCOLATE,NONFAT MILK,W/ LOW-CAL SWEET |
| **11460410** | YOGURT,FRZ,NOT CHOC,NONFAT MILK,W/ LOW-CAL SWEET |
| **11460420** | YOGURT, FROZEN, NS AS TO FLAVOR, WHOLE MILK |
| **11460430** | YOGURT, FROZEN, CHOCOLATE, WHOLE MILK |
| **11460440** | YOGURT, FROZEN, NOT CHOCOLATE, WHOLE MILK |
| **11460500** | FROZEN YOGURT, SOFT SERVE, VANILLA |
| **11460510** | FROZEN YOGURT, SOFT SERVE, CHOCOLATE |
| **11461000** | YOGURT, FROZEN, CHOCOLATE-COATED |
| **11461100** | YOGURT, FROZEN, CAROB-COATED |
| **11461200** | YOGURT, FROZEN, SANDWICH |
| **11461210** | FROZEN YOGURT BAR, VANILLA |
| **11461220** | FROZEN YOGURT BAR, CHOCOLATE |
| **11461250** | YOGURT, FROZEN, CONE, CHOCOLATE |
| **11461260** | YOGURT, FROZEN, CONE, NOT CHOCOLATE |
| **11461270** | YOGURT, FROZEN, CONE, NOT CHOCOLATE, LOWFAT MILK |
| **11461280** | YOGURT, FROZ, CONE, CHOCOLATE, LOWFAT MILK |
| **11461300** | FROZEN YOGURT CONE, VANILLA, WAFFLE CONE |
| **11461320** | FROZEN YOGURT CONE, CHOCOLATE, WAFFLE CONE |
| **11480010** | YOGURT, WHOLE MILK, BF |
| **11480020** | YOGURT, WHOLE MILK, BF, W/FRUIT& MULTIGRAIN CEREAL,NFS |
| **11480030** | YOGURT, WHOLE MILK, BF, W/FRUIT&MULTIGRAIN CEREAL + IRON |
| **11480040** | YOGURT, WHOLE MILK, BF, W/FRUIT&MULTIGRAIN CEREAL + DHA |
| **11480100** | BABY TODDLER YOGURT, WITH FRUIT |
| **14210000** | CHEESE, YOGURT, NFS |
| **41420200** | NATTO (FERMENTED SOYBEAN PRODUCT) |
| **41420380** | TOFU YOGURT |
| **42401100** | YOGURT, COCONUT MILK |
| **53104580** | CHEESECAKE-TYPE DESSERT, MADE W/ YOGURT, W/ FRUIT |
| **53112150** | CAKE,FROZEN YOGURT & CAKE LAYER,NOT CHOC,W/ ICING |
| **53112160** | CAKE,FROZEN YOGURT & CAKE LAYER,CHOCOLATE,W/ ICING |
| **53366000** | PIE, YOGURT, FROZEN |
| **53540402** | KELLOGG'S NUTRI-GRAIN YOGURT BAR |
| **53540500** | BREAKFAST BAR, DATE, W/ YOGURT COATING |
| **53540902** | NATURE VALLEY CHEWY GRANOLA BAR W/YOGURT COATING |
| **53544300** | GRANOLA BAR, HIGH FIBER, YOGURT COATING, NOT CHOC |
| **53710502** | KELLOGG'S NUTRI-GRAIN YOGURT BAR |
| **53710902** | NATURE VALLEY CHEWY GRANOLA BAR WITH YOGURT COATING |
| **53714300** | GRANOLA BAR, HIGH FIBER, COATED W/ NON-CHOC YOGURT COATING |
| **53714510** | BREAKFAST BAR, DATE, WITH YOGURT COATING |
| **54408250** | PRETZEL, YOGURT COVERED |
| **54430010** | YOGURT CHIPS |
| **57344015** | SPECIAL K FRUIT & YOGURT |
| **57419000** | YOGURT BURST CHEERIOS |
| **63401015** | APPLE AND GRAPE SALAD W/ YOGURT & WALNUTS |
| **67100260** | BABY TODDLER FRUIT, WITH YOGURT |
| **67100460** | BABY TODDLER FRUIT AND VEGETABLES, WITH YOGURT |
| **67250100** | BANANA JUICE W/ LOWFAT YOGURT, BABY FOOD |
| **67250150** | MIXED FRUIT JUICE W/ LOWFAT YOGURT, BABY FOOD |
| **67404070** | APPLE YOGURT DESSERT, BABY, STRAINED |
| **67404300** | BLUEBERRY YOGURT DESSERT, BABY, STRAINED |
| **67404500** | MIXED FRUIT YOGURT DESSERT, BABY, STRAINED |
| **67408500** | BANANA YOGURT DESSERT, BABY, STRAINED |
| **67413700** | PEACH YOGURT DESSERT, BABY, STRAINED |
| **67430500** | YOGURT AND FRUIT SNACK, BABY FOOD |
| **75230000** | SAUERKRAUT, NS AS TO ADDED FAT |
| **75230010** | SAUERKRAUT, NO FAT ADDED |
| **75230020** | SAUERKRAUT, FAT ADDED |
| **75230100** | SAUERKRAUT, CANNED, LO NA |
| **75502520** | CABBAGE, KIMCHI (KIM CHEE) STYLE |
| **75503010** | CUCUMBER PICKLES, DILL |
| **75503020** | CUCUMBER PICKLES, RELISH |
| **75503030** | CUCUMBER PICKLES, SOUR |
| **75503040** | CUCUMBER PICKLES, SWEET |
| **75503050** | CUCUMBER PICKLES, FRESH (INCLUDE BREAD & BUTTER) |
| **75503100** | MUSTARD PICKLES (INCL CHOW-CHOW, HOT DOG RELISH) |
| **75503110** | CUCUMBER PICKLES, DILL, REDUCED SALT |
| **75503140** | CUCUMBER PICKLES, SWEET, REDUCED SALT |
| **75510000** | OLIVES, NFS |
| **75510010** | OLIVES, GREEN |
| **75510020** | OLIVES, BLACK |
| **75510030** | OLIVES, GREEN, STUFFED |
| **75510050** | OLIVE TAPENADE |
| **75511100** | PICKLES, NS AS TO VEGETABLE |
| **75511200** | PICKLES, MIXED |
| **75511300** | PICKLES, FRIED |
| **75534500** | TSUKEMONO, JAPANESE PICKLES |
| **75601250** | KIMCHI SOUP |
| **81103041** | MARGARINE-LIKE SPREAD, MADE W/ YOGURT, STICK, SALTED |
| **81104011** | MARGARINE-LIKE SPREAD,RED CAL,40% FAT,MADE W/ YOGURT,TUB |
| **83107100** | MAYONNAISE, MADE W/ YOGURT (INCLUDE YOGANNAISE) |
| **83115000** | YOGURT DRESSING |
| **83204030** | MAYONNAISE, REGULAR, �WITH OLIVE OIL |
| **91701030** | ALMONDS, YOGURT-COVERED |
| **91708150** | YOGURT COVERED FRUIT SNACKS CANDY, W/ ADDED VITAMIN C |
| **91708160** | YOGURT COVERED FRUIT SNACKS CANDY ROLLS, W/ HIGH VITAMIN C |
| **91731150** | PEANUTS, YOGURT-COVERED |
| **91739600** | RAISINS, YOGURT-COVERED |
| **92306920** | TEA, KOMBUCHA |

Supplemental Table 8: List of Probiotic Supplements

| **Supplement ID** | **Supplement Description** |
| --- | --- |
| **1000317600** | NATURE'S BOUNTY ACIDOPHILUS |
| **1000315000** | NATURE'S SUNSHINE BIFIDOPHILUS FLORA FORCE BIFIDOBACTERIUM LONGUM & LACTOBACILLUS ACIDOPHILUS 470 MG |
| **1000251300** | KYO-DOPHILUS |
| **1000191100** | SOLGAR ADVANCED 40+ ACIDOPHILUS NON-DAIRY LACTOSE FREE |
| **1888226201** | DEFAULT LACTOBACILLUS ACIDOPHILUS |
| **1000235400** | TWINLAB ALLERDOPHILUS ACIDOPHILUS CAPSULES HIGH POTENCY 182 MG |
| **1888226201** | DEFAULT LACTOBACILLUS ACIDOPHILUS |
| **1000299400** | NATURE'S WAY ONCE DAILY PRIMADOPHILUS |
| **1888226201** | DEFAULT LACTOBACILLUS ACIDOPHILUS |
| **1000286300** | SAV-ON OSCO ONE CHOICE THE MOST COMPLETE MULTIVITAMIN WITH HERBS WITH FLORAGLO LUTEIN |
| **1000153700** | FLORADIX TABLETS WITH IRON, VITAMINS, YEAST AND HERB EXTRACTS |
| **1888226201** | DEFAULT LACTOBACILLUS ACIDOPHILUS |
| **1000153700** | FLORADIX TABLETS WITH IRON, VITAMINS, YEAST AND HERB EXTRACTS |
| **1000257000** | WALGREENS ACIDOPHILUS 10 MG |
| **1000299400** | NATURE'S WAY ONCE DAILY PRIMADOPHILUS |
| **1000242000** | AMERICAN HEALTH CHEWABLE ACIDOPHILUS WITH BIFIDUS MILK FREE |
| **1000318100** | SPRING VALLEY ACIDOPHILUS |
| **1000374700** | BACID PROBIOTIC CONTAINS A BLEND OF BENEFICIAL BACTERIA INCLUDING LACTOBACILLUS ACIDOPHILUS |
| **1000310500** | PRO-BIOTICS ACIDOPHILUS |
| **1000301000** | HERBALIFE FLORAFIBER LACTOBACILLUS ACIDOPHILUS AND FIBER |
| **1000398000** | COUNTRY LIFE POWER-DOPHILUS WITH FOS HYPOALLERGENIC MILK FREE |
| **1000056900** | PURITAN'S PRIDE POTENT ACIDOPHILUS WITH PECTIN |
| **1888226201** | DEFAULT LACTOBACILLUS ACIDOPHILUS |
| **1000324000** | SHAKLEE BIFIDUS & ACIDOPHILUS OPTIFLORA PROBIOTIC COMPLEX |
| **1000324100** | SHAKLEE OPTIFLORA FOS, INULIN, & MORE PREBIOTIC DIETARY SUPPLEMENT |
| **1000287000** | SAV-ON OSCO CENTRAL-VITE SELECT MULTIVITAMIN/MULTIMINERAL FOR MATURE ADULTS WITH FLORAGLO LUTEIN |
| **1000287000** | SAV-ON OSCO CENTRAL-VITE SELECT MULTIVITAMIN/MULTIMINERAL FOR MATURE ADULTS WITH FLORAGLO LUTEIN |
| **1000425300** | FLORADIX KINDER LOVE CHILDREN'S MULTIVITAMIN LIQUID EXTRACT FORMULA VITAMINS A, B, C, D AND E WITH CALCIUM, HERBAL EXTRA |
| **1000362400** | VITAMIN WORLD CHEWABLE ACIDOPHILUS WITH BIFIDUS NATURAL STRAWBERRY FLAVOR |
| **1000287000** | SAV-ON OSCO CENTRAL-VITE SELECT MULTIVITAMIN/MULTIMINERAL FOR MATURE ADULTS WITH FLORAGLO LUTEIN |
| **1000459000** | RBC DIGESTION FORMULA WITH ENZYMES, PROBIOTICS AND NANOCLUSTERS |
| **1000242000** | AMERICAN HEALTH CHEWABLE ACIDOPHILUS WITH BIFIDUS MILK FREE |
| **1000251300** | KYO-DOPHILUS |
| **1888226201** | DEFAULT LACTOBACILLUS ACIDOPHILUS |
| **1000459600** | NATREN'S MEGA DOPHILUS DAIRY POWDER |
| **1888226201** | DEFAULT LACTOBACILLUS ACIDOPHILUS |
| **1000318100** | SPRING VALLEY ACIDOPHILUS |
| **1000191100** | SOLGAR ADVANCED 40+ ACIDOPHILUS NON-DAIRY LACTOSE FREE |
| **1000242000** | AMERICAN HEALTH CHEWABLE ACIDOPHILUS WITH BIFIDUS MILK FREE |
| **1000497100** | PROBIOTICA LACTOBACILLUS REUTERI CHEWABLE TABLETS |
| **1888226201** | DEFAULT LACTOBACILLUS ACIDOPHILUS |
| **1000279700** | GNC NATURAL BRAND ULTRA ACIDOPHILUS 350 MG |
| **1000317600** | NATURE'S BOUNTY ACIDOPHILUS |
| **1000455700** | O'DONNELL FORMULAS, INC. FLORA-BALANCE BACILLUS LATEROSPORUS BOD STRAIN CAPSULES |
| **1000242000** | AMERICAN HEALTH CHEWABLE ACIDOPHILUS WITH BIFIDUS MILK FREE |
| **1888226201** | DEFAULT LACTOBACILLUS ACIDOPHILUS |
| **1000447000** | FLORA FLOR-ESSENCE |
| **1888324001** | DEFAULT PROBIOTIC |
| **1000422400** | BREAKTHROUGH FORMULATIONS MEGA-FLORA DIGESTIVE TRACT FLORA |
| **1000324000** | SHAKLEE BIFIDUS & ACIDOPHILUS OPTIFLORA PROBIOTIC COMPLEX |
| **1000279700** | GNC NATURAL BRAND ULTRA ACIDOPHILUS 350 MG |
| **1000318100** | SPRING VALLEY ACIDOPHILUS |
| **1000459000** | RBC DIGESTION FORMULA WITH ENZYMES, PROBIOTICS AND NANOCLUSTERS |
| **1000428700** | SALUS FLORADIX CALCIUM LIQUID MINERAL NO ADDED SUGAR HERBAL |
| **1000366800** | NATURE'S WAY ONCE DAILY PRIMADOPHILUS FOR CHILDREN |
| **1000536900** | NATURE'S LIFE LACTOBACILLUS ACIDOPHILUS APPLE PECTIN LIVE, ACTIVE ORGANISMS PROBIOTIC CAPSULES |
| **1000425300** | FLORADIX KINDER LOVE CHILDREN'S MULTIVITAMIN LIQUID EXTRACT FORMULA VITAMINS A, B, C, D AND E WITH CALCIUM, HERBAL EXTRA |
| **1000301000** | HERBALIFE FLORAFIBER LACTOBACILLUS ACIDOPHILUS AND FIBER |
| **1000317600** | NATURE'S BOUNTY ACIDOPHILUS |
| **1000443800** | ALLERGY RESEARCH GROUP GI FLORA L.ACIDOPHILUS, L.CASEI, L. RHAMNOSUS, B. LONGUM HYPOALLERGENIC |
| **1000318100** | SPRING VALLEY ACIDOPHILUS |
| **1000371700** | HEALTH PLUS INC. SUPER COLON CLEANSE WITH HERBS & ACIDOPHILUS |
| **1000425300** | FLORADIX KINDER LOVE CHILDREN'S MULTIVITAMIN LIQUID EXTRACT FORMULA VITAMINS A, B, C, D AND E WITH CALCIUM, HERBAL EXTRA |
| **1000412300** | GNC NATURAL BRAND POTENT ACIDOPHILUS |
| **1000507900** | GNC NATURAL BRAND MEGA ACIDOPHILUS |
| **1000287000** | SAV-ON OSCO CENTRAL-VITE SELECT MULTIVITAMIN/MULTIMINERAL FOR MATURE ADULTS WITH FLORAGLO LUTEIN |
| **1888226201** | DEFAULT LACTOBACILLUS ACIDOPHILUS |
| **1000529600** | ECOQUEST / INFINITY ESSENTIALS FOR LIFE 2 ENZYME, FLORA AND CHROMIUM COMPLEX |
| **1000310500** | PRO-BIOTICS ACIDOPHILUS |
| **1000483800** | ENZYMATIC THERAPY NATURAL MEDICINES ACIDOPHILUS PEARLS WITH L. ACIDOPHILUS AND B. LONGUM |
| **1000541800** | WALGREENS ACIDOPHILUS PLUS CITRUS PECTIN FREEZE DRIED |
| **1000517800** | NATREN'S MEGA DOPHILUS DAIRY CAPSULES |
| **1000317601** | NATURE'S BOUNTY PROBIOTIC ACIDOPHILUS |
| **1888226201** | DEFAULT LACTOBACILLUS ACIDOPHILUS |
| **1000490200** | FLORADIX CALCIUM-MAGNESIUM WITH ZINC, VITAMIN D AND HERBS |
| **1000301000** | HERBALIFE FLORAFIBER LACTOBACILLUS ACIDOPHILUS AND FIBER |
| **1000542200** | FLORA CERTIFIED ORGANIC FLAX OIL COLD-PRESSED & UNREFINED |
| **1888226201** | DEFAULT LACTOBACILLUS ACIDOPHILUS |
| **1000378001** | NEW CHAPTER EVERY MAN II WHOLE-FOOD PROBIOTIC MULTI-NUTRIENT, MINERAL & HERBAL COMPLEX |
| **1888226201** | DEFAULT LACTOBACILLUS ACIDOPHILUS |
| **1000530800** | NATURE MADE ACIDOPHILUS 500 MILLION LIVE CELLS PER TABLET |
| **1000484500** | FLORA BILBERRY EXTRACT 25% STANDARDIZED ANTHOCYANIDINS WITH FREEZE-DRIED BLUEBERRY POWDER |
| **1000287000** | SAV-ON OSCO CENTRAL-VITE SELECT MULTIVITAMIN/MULTIMINERAL FOR MATURE ADULTS WITH FLORAGLO LUTEIN |
| **1000317601** | NATURE'S BOUNTY PROBIOTIC ACIDOPHILUS |
| **1000056800** | PURITAN'S PRIDE ACIDOPHILUS |
| **1000319800** | SUNDOWN ACIDOPHILUS XTRA |
| **1000522900** | PB8 PRO-BIOTIC ACIDOPHILUS FOR LIFE |
| **1000287000** | SAV-ON OSCO CENTRAL-VITE SELECT MULTIVITAMIN/MULTIMINERAL FOR MATURE ADULTS WITH FLORAGLO LUTEIN |
| **1000553700** | JARROW FORMULAS BABY'S JARRO-DOPHILUS WITH CLINICALLY DOCUMENTED STRAINS 3 BILLION PER GRAM HYPOALLERGENIC NON-DAIRY PRO |
| **1000522900** | PB8 PRO-BIOTIC ACIDOPHILUS FOR LIFE |
| **1000524400** | METAGENICS ULTRA FLORA PLUS DF CAPSULES |
| **1000242000** | AMERICAN HEALTH CHEWABLE ACIDOPHILUS WITH BIFIDUS MILK FREE |
| **1000599700** | NEW CHAPTER EVERY WOMAN'S ONE DAILY WHOLE-FOOD PROBIOTIC MULTI-VITAMIN, MINERAL & HERBAL COMPLEX |
| **1000634200** | PROBIOTIC PEARLS WITH L. ACIDOPHILUS AND B. LONGUM |
| **1000629500** | CULTURELLE WITH LACTOBACILLUS GG |
| **1000628500** | NATURE'S WAY ONCE DAILY PRIMADOPHILUS KIDS TRUE POTENCY 3 BILLION CFU TRUE IDENTITY LACTOBACILLI & BIFIDOBACTERIA ORANGE |
| **1000661200** | NATURE'S WAY PRIMADOPHILUS BIFIDUS ONCE DAILY TRUE POTENCY 5 BILLION CFU TRUE IDENTITY BIFIDOBACTERIA & LACTOBACILLI TRU |
| **1000317601** | NATURE'S BOUNTY PROBIOTIC ACIDOPHILUS |
| **1000599600** | NEW CHAPTER EVERY WOMAN WHOLE-FOOD PROBIOTIC MULTI-VITAMIN, MINERAL & HERBAL COMPLEX |
| **1000605900** | PURE RESEARCH PRODUCTS, LLC DEL-IMMUNE V LACTOBACILLUS RHAMNOSUS LYSED POWDER |
| **1000607500** | MEGAFLORA OPTIMAL POTENCY PROBIOTIC FORMULA 100% WHOLE FOOD MEGAFOOD |
| **1000629000** | NEW CHAPTER ORGANICS PROBIOTIC NUTRIENTS PERFECT PRENATAL |
| **1888226201** | DEFAULT LACTOBACILLUS ACIDOPHILUS |
| **1888593301** | GENERIC8 ACIDOPHILUS & PROBIOTIC COMPLEX |
| **1000366802** | NATURE'S WAY ONCE DAILY PRIMADOPHILUS CHILDREN POWDER WITH FOS TRUE IDENTITY BIFIDOBACTERIA & LACTOBACILLI TRUE POTENCY |
| **1000671500** | LIFETIME LIQUID ACIDOPHILUS SOY BASE MILK FREE |
| **1000600400** | FLORA WHITE WILLOW BARK 55 MG STANDARDIZED SALICIN |
| **1000602000** | JARROW FORMULAS JARRO-DOPHILUS ORIGINAL WITH CLINICALLY PROVEN STRAIN BB536 HIGH POTENCY 6 SPECIES 3.4 BILLION |
| **1000627800** | DDS-100 ACIDOPHILUS WITH FOS CHEWABLE TABLETS (2 BILLION CFU/G) UAS LABORATORIES |
| **1000301000** | HERBALIFE FLORAFIBER LACTOBACILLUS ACIDOPHILUS AND FIBER |
| **1000317601** | NATURE'S BOUNTY PROBIOTIC ACIDOPHILUS |
| **1000667700** | ENERGETIX FLORA SYNERGY |
| **1000371700** | HEALTH PLUS INC. SUPER COLON CLEANSE WITH HERBS & ACIDOPHILUS |
| **1000324000** | SHAKLEE BIFIDUS & ACIDOPHILUS OPTIFLORA PROBIOTIC COMPLEX |
| **1000612000** | GNC NATURAL BRAND ACIDOPHILUS 2 BILLION CFU |
| **1000586100** | FLORA UDO'S CHOICE WHOLESOME FAST FOOD ALL-VEGETARIAN FIBER, ENZYMES & PHYTONUTRIENTS |
| **1000586200** | FLORA UDO'S CHOICE OIL BLEND CERTIFIED ORGANIC BLEND OF FLAX AND OTHER NUTRITIONALLY SUPERIOR OILS |
| **1000583400** | METAGENICS LACTO VIDEN ID DAIRY FREE, STRAIN MODIFIED LACTOBACILLUS BLEND |
| **1000661200** | NATURE'S WAY PRIMADOPHILUS BIFIDUS ONCE DAILY TRUE POTENCY 5 BILLION CFU TRUE IDENTITY BIFIDOBACTERIA & LACTOBACILLI TRU |
| **1000651400** | DIGESTIVE ENZYMES & PROBIOTICS WITH ENZYGUARD-D 4LIFE |
| **1000607400** | GARDEN OF LIFE PRIMAL DEFENSE HSO PROBIOTIC FORMULA |
| **1000655200** | NUTRICOLOGY PROGREENS WITH ADVANCED PROBIOTIC FORMULA |
| **1000611500** | NATROL ACIDOPHILUS PROBIOTIC |
| **1888226201** | DEFAULT LACTOBACILLUS ACIDOPHILUS |
| **1000605900** | PURE RESEARCH PRODUCTS, LLC DEL-IMMUNE V LACTOBACILLUS RHAMNOSUS LYSED POWDER |
| **1000522900** | PB8 PRO-BIOTIC ACIDOPHILUS FOR LIFE |
| **1000599800** | NEW CHAPTER VITAMIN B COMPLEX WHOLE-FOOD HERBAL PROBIOTIC NUTRIENT COMPLEX |
| **1000599900** | NEW CHAPTER ORGANICS PROBIOTIC NUTRIENTS EVERY MAN |
| **1000605900** | PURE RESEARCH PRODUCTS, LLC DEL-IMMUNE V LACTOBACILLUS RHAMNOSUS LYSED POWDER |
| **1000651400** | DIGESTIVE ENZYMES & PROBIOTICS WITH ENZYGUARD-D 4LIFE |
| **1000318101** | SPRING VALLEY ACIDOPHILUS |
| **1888226201** | DEFAULT LACTOBACILLUS ACIDOPHILUS |
| **1000599700** | NEW CHAPTER EVERY WOMAN'S ONE DAILY WHOLE-FOOD PROBIOTIC MULTI-VITAMIN, MINERAL & HERBAL COMPLEX |
| **1000610500** | GNC NATURAL BRAND ACIDOPHILUS 4 BILLION CFU |
| **1000610100** | PROCAPS LABORATORIES FIBERMUCIL U.S.P. PSYLLIUM BULK AND WATER SOLUBLE FIBER FRIENDLY FLORA LACTOBACILLUS |
| **1000251300** | KYO-DOPHILUS |
| **1000629600** | JARROW FORMULAS ENHANCED PROBIOTIC SYSTEM JARRO-DOPHILUS EPS ENTERIC COATED |
| **1888597601** | GENERIC20 GR8-DOPHILUS 8 STRAINS & 4 BILLION POTENCY WITH FOS |
| **1000607400** | GARDEN OF LIFE PRIMAL DEFENSE HSO PROBIOTIC FORMULA |
| **1000056900** | PURITAN'S PRIDE POTENT ACIDOPHILUS WITH PECTIN |
| **1000589900** | OPTIMUM FREEZE DRIED ACIDOPHILUS LACTOBACILLI |
| **1000605900** | PURE RESEARCH PRODUCTS, LLC DEL-IMMUNE V LACTOBACILLUS RHAMNOSUS LYSED POWDER |
| **1000611300** | THE VITAMIN SHOPPE ACIDOPHILUS WITH PECTIN |
| **1000634200** | PROBIOTIC PEARLS WITH L. ACIDOPHILUS AND B. LONGUM |
| **1000860400** | BIOSPEC PROBIOTIC PLUS HIGH POTENCY, MULTI-STRAIN PROBIOTIC |
| **1000873800** | NATURE'S SUNSHINE PROBIOTIC ELEVEN |
| **1000747800** | NOW BERRY DOPHILUS 4 PROBIOTIC STRAINS 2.5 BILLION INPUT NATURAL BERRY FLAVOR XYLITOL SWEETENED CHEWABLES |
| **1000830700** | ALIGN PROBIOTIC |
| **1000617301** | RAINBOW LIGHT JUST 1 ONCE NATURALS WOMEN'S ONE MULTIVITAMIN/MINERAL NOW WITH PROBIOTICS |
| **1000617301** | RAINBOW LIGHT JUST 1 ONCE NATURALS WOMEN'S ONE MULTIVITAMIN/MINERAL NOW WITH PROBIOTICS |
| **1888226201** | DEFAULT LACTOBACILLUS ACIDOPHILUS |
| **1888324001** | DEFAULT PROBIOTIC |
| **1000875800** | SPRING VALLEY 1 PER DAY DOSE ALL NATURAL CRANBERRY WITH VITAMIN C & PROBIOTICS NEW IMPROVED FORMULA |
| **1000830700** | ALIGN PROBIOTIC |
| **1000830700** | ALIGN PROBIOTIC |
| **1000854700** | SUSTENEX DAILY PROBIOTIC 2 BILLION CELLS OF GANEDENBC30 |
| **1888324001** | DEFAULT PROBIOTIC |
| **1000774000** | NATURE'S BOUNTY PROBIOTIC ACIDOPHILUS CAPSULES |
| **1000800600** | BLUEBONNET MILK-FREE ACIDOPHILUS PLUS FOS L. ACIDOPHILUS, L. BULGARICUS, BIFIDUS |
| **1000522901** | NUTRITION NOW PB8 PRO-BIOTIC ACIDOPHILUS FOR LIFE |
| **1000812800** | VITAMIN WORLD ACIDOPHILUS & PSYLLIUM HUSK 4 BILLION ACTIVE CULTURES 4,000 MG PSYLLIUM HUSK PER SERVING |
| **1888324001** | DEFAULT PROBIOTIC |
| **1888324001** | DEFAULT PROBIOTIC |
| **1000617301** | RAINBOW LIGHT JUST 1 ONCE NATURALS WOMEN'S ONE MULTIVITAMIN/MINERAL NOW WITH PROBIOTICS |
| **1000242001** | AMERICAN HEALTH CHEWABLE MILK FREE ACIDOPHILUS AND BIFIDUM ONE BILLION ORGANISMS VEGETARIAN FORMULA |
| **1000870200** | RAINBOW LIGHT 50+ MINI-TAB AGE-DEFENSE FORMULA FOOD-BASED MULTIVITAMIN WITH COQ10 1,000 IU VITAMIN D3 PROBIOTICS & DIGES |
| **1888226201** | DEFAULT LACTOBACILLUS ACIDOPHILUS |
| **1000742100** | NSI NUTRACEUTICAL SCIENCES INSTITUTE PROBIOTIC 15-35 15 STRAINS / 35 BILLION MICROORGANISMS PER SERVING |
| **1888226201** | DEFAULT LACTOBACILLUS ACIDOPHILUS |
| **1000628500** | NATURE'S WAY ONCE DAILY PRIMADOPHILUS KIDS TRUE POTENCY 3 BILLION CFU TRUE IDENTITY LACTOBACILLI & BIFIDOBACTERIA ORANGE |
| **1888324001** | DEFAULT PROBIOTIC |
| **1000880800** | FRIENDLY FLORA PREBIOTIC AND PROBIOTIC COMPLEX FOS AND LACTOBACILLUS 5 BILLION CFU PROCAPS LABORATORIES |
| **1000889200** | GARDEN OF LIFE RAW VITAMIN CODE 50 & WISER WOMEN RAW WHOLE FOOD MULTI LIVE PROBIOTICS & ENZYMES RAW FOOD-CREATED NUTRIEN |
| **1000629500** | CULTURELLE WITH LACTOBACILLUS GG |
| **1000798800** | FLORASTOR KIDS SACCHAROMYCES BOULARDII LYO PACKETS 250 MG |
| **1000823000** | REXALL NATURALS GESTAZYME MULTI-ENZYME PROBIOTIC FORMULA ACIDOPHILUS PROBIOTIC FORMULA 1 BILLION ACTIVE CULTURES PER SER |
| **1000874700** | ORIGIN ACIDOPHILUS EASY TO SWALLOW |
| **1000888800** | STOCKBRIDGE NATURALS TRI-DOPHILUS |
| **1000885701** | SPRING VALLEY PROBIOTIC ACIDOPHILUS 1 BILLION ACTIVE CULTURES |
| **1000857100** | NATURE'S PLUS SOURCE OF LIFE ANIMAL PARADE ACIDOPHIKIDZ WITH PROBIOTICS, FOS & RHODODENDRON CHILDREN'S CHEWABLE WITH WHO |
| **1000319801** | SUNDOWN NATURALS PROBIOTIC ACIDOPHILUS XTRA 40 MILLION LIVE CELLS PER SERVING VEGETARIAN FORMULA |
| **1000617301** | RAINBOW LIGHT JUST 1 ONCE NATURALS WOMEN'S ONE MULTIVITAMIN/MINERAL NOW WITH PROBIOTICS |
| **1000896200** | RITE AID PHARMACY PROBIOTIC COLON CARE LACTOBACILLUS ACIDOPHILUS, BIFIDOBACTERIUM LONGUM, B. BIFIDUM PROBIOTIC ONE DAILY |
| **1000865500** | SPRING VALLEY SUPER STRENGTH PROBIOTIC ACIDOPHILUS 2 BILLION ACTIVE CULTURES |
| **1000737800** | REXALL NATURALS ULTIMATE PROBIOTIC FORMULA ACIDOPHILUS MADE WITH BIO FLORA 2 BILLION ACTIVE CULTURES |
| **1000892300** | DIGESTIVE ADVANTAGE LACTOSE DEFENSE FORMULA DUAL-ACTION! ENZYME PLUS PROBIOTICS TAKE EVERY DAY |
| **1000840100** | GARDEN OF LIFE PRIMAL DEFENSE ULTRA ULTIMATE PROBIOTIC FORMULA 15 BILLION CELLS DAILY 13 BENEFICIAL CULTURES WITH HSOS W |
| **1888226201** | DEFAULT LACTOBACILLUS ACIDOPHILUS |
| **1000830400** | RAINBOW LIGHT ACTIVE HEALTH TEEN FOOD-BASED MULTIVITAMIN TARGETED TEEN NUTRITION 75 MILLION LIVE ACTIVE PROBIOTICS |
| **1000798200** | FLORAJEN3 HIGH POTENCY PROBIOTIC ACIDOPHILUS BIFIDUM LONGUM 15 BILLION LIVE CULTURES PER CAPSULE |
| **1000870200** | RAINBOW LIGHT 50+ MINI-TAB AGE-DEFENSE FORMULA FOOD-BASED MULTIVITAMIN WITH COQ10 1,000 IU VITAMIN D3 PROBIOTICS & DIGES |
| **1000854300** | VIBRANT HEALTH GREEN VIBRANCE 25 BILLION PROBIOTICS PER DOSE FROM 12 STRAINS ORGANIC GREENS & FREEZE DRIED GRASS JUICES |
| **1000629500** | CULTURELLE WITH LACTOBACILLUS GG |
| **1000850500** | NATURE'S PLUS ADULT'S EAR, NOSE & THROAT LOZENGES WITH K12 PROBIOTICS |
| **1000971900** | PURITAN'S PRIDE VITA-FRESH LIFE'S GREENS WITH ADVANCED PROBIOTIC FORMULA 29,205 TOTAL ORAC VALUE VEGETARIAN |
| **1000946900** | NATURE'S WAY PRIMADOPHILUS REUTERI SUPERIOR PROBIOTIC MULTI STRAIN PLUS SCFOS TRUE POTENCY 5 BILLION CFU ENTERIC-COATED ONCE DAILY |
| **1888324001** | DEFAULT PROBIOTIC |
| **1888324001** | DEFAULT PROBIOTIC |
| **1000917300** | GNC WOMEN'S ULTRA MEGA WITH PROBIOTICS MULTIVITAMIN DELIVERS 1 BILLION LIVE, ACTIVE CULTURES WITH 1,600 IU OF VITAMIN D-3 |
| **1000885701** | SPRING VALLEY PROBIOTIC ACIDOPHILUS 1 BILLION ACTIVE CULTURES |
| **1888226201** | DEFAULT LACTOBACILLUS ACIDOPHILUS |
| **1000991900** | Y.S. ORGANIC BEE FARMS 100% PURE BEE POLLEN CAPSULES MULTI FLORAL, WILD CRAFTED |
| **1000725801** | RAINBOW LIGHT MEN'S ONE JUST 1 ONCE FOOD-BASED MULTIVITAMIN ENERGY B-COMPLEX & PROBIOTICS |
| **1000948600** | RENEW LIFE FLORABEAR FOR KIDS |
| **1888324001** | DEFAULT PROBIOTIC |
| **1888226201** | DEFAULT LACTOBACILLUS ACIDOPHILUS |
| **1000725802** | RAINBOW LIGHT MEN'S ONE JUST 1 ONCE FOOD-BASED MULTIVITAMIN ENERGY B-COMPLEX & PROBIOTICS |
| **1000725801** | RAINBOW LIGHT MEN'S ONE JUST 1 ONCE FOOD-BASED MULTIVITAMIN ENERGY B-COMPLEX & PROBIOTICS |
| **1000522902** | NUTRITION NOW PB8 PRO-BIOTIC ACIDOPHILUS FOR LIFE ORIGINAL FORMULA 14 BILLION GOOD BACTERIA |
| **1000953600** | THE VITAMIN SHOPPE ULTIMATE '10' PROBIOTIC 13 BILLION ORGANISMS WITH FRUCTO-OLIGOSACCHARIDES ENTERIC COATED |
| **1000994700** | FUTUREBIOTICS COLON GREEN GENTLE, ALL-NATURAL FIBER WITH PROBIOTICS AND ENZYMES NOW WITH SUPER-STABLE PROBIOTICS |
| **1000903100** | PHILLIPS' COLON HEALTH PROBIOTIC CAPS BAYER |
| **1000934200** | FLORADIX FLORAVITAL IRON + HERBS LIQUID EXTRACT FORMULA RICH IN IRON AND B-VITAMINS VEGETARIAN LIQUID FORMULA |
| **1000961000** | WINDMILL NATURAL ACIDOPHILUS PRO-BIOTIC BLEND WITH PECTIN |
| **1000984700** | NATURE CITY TRUELIFE PB PROBIOTIC AND PREBIOTIC BLEND PROFESSIONAL STRENGTH! 30 BILLION LIVE 'GOOD BACTERIA' CELLS PER DOSE PROVIDES 6 HEALTH BOOSTING PROBIOTIC STRAINS |
| **1888226201** | DEFAULT LACTOBACILLUS ACIDOPHILUS |
| **1000593301** | TRADER JOE'S ACIDOPHILUS & PROBIOTIC COMPLEX 2 BILLION ORGANISMS PER TABLET |
| **1000951900** | 4X PROBIOTIC UP&UP CONTAINS 4 STRAINS OF NATURAL, BENEFICIAL BACTERIA WITH B. INFANTIS 10 MG |
| **1000972400** | NATURE'S WAY PRIMADOPHILUS ORIGINAL ONCE DAILY TRUE POTENCY 5 BILLION CFU TRUE IDENTITY ACIDOPHILUS & RHAMNOSUS TRUE RELEASE TARGETED DELIVERY ENTERIC-COATED FOR ALL AGES |
| **1000885701** | SPRING VALLEY PROBIOTIC ACIDOPHILUS 1 BILLION ACTIVE CULTURES |
| **1000553702** | JARROW FORMULAS BABY JARRO-DOPHILUS +FOS WITH CLINICALLY DOCUMENTED STRAINS 3 BILLION PROBIOTIC |
| **1000951200** | SCHIFF SUSTENEX PROBIOTIC GUMMIES |
| **1000965000** | GARDEN OF LIFE RAW PROBIOTICS WOMEN 50 & WISER 85 BILLION LIVE CULTURES 33 PROBIOTIC STRAINS PROBIOTIC-CREATED VITAMINS, MINERALS, ENZYMES & PREBIOTICS CONTAINS TARGETED PROBIOTICS RAW WHOLE FOOD |
| **1000885701** | SPRING VALLEY PROBIOTIC ACIDOPHILUS 1 BILLION ACTIVE CULTURES |
| **1000910500** | SPRING VALLEY PROBIOTIC MULTI-ENZYME DIGESTIVE FORMULA 1 BILLION ACTIVE CULTURES PER SERVING |
| **1000737800** | REXALL NATURALS ULTIMATE PROBIOTIC FORMULA ACIDOPHILUS MADE WITH BIO FLORA 2 BILLION ACTIVE CULTURES |
| **1000242002** | AMERICAN HEALTH CHEWABLE MILK FREE ACIDOPHILUS AND BIFIDUM ONE BILLION ORGANISMS VEGETARIAN FORMULA |
| **1888226201** | DEFAULT LACTOBACILLUS ACIDOPHILUS |
| **1000607400** | GARDEN OF LIFE PRIMAL DEFENSE HSO PROBIOTIC FORMULA |
| **1000916400** | ULTIMATE FLORA CRITICAL CARE 50 BILLION EXTRA-STRENGTH PROBIOTIC 10 PROBIOTIC STRAINS RENEW LIFE |
| **1000315001** | NATURE'S SUNSHINE BIFIDOPHILUS FLORA FORCE PROBIOTIC 4 BILLION TOTAL MICROORGANISMS PER CAPSULE |
| **1000880800** | FRIENDLY FLORA PREBIOTIC AND PROBIOTIC COMPLEX FOS AND LACTOBACILLUS 5 BILLION CFU PROCAPS LABORATORIES |
| **1000908400** | REPHRESH PRO-B PROBIOTIC FEMININE |
| **1000972400** | NATURE'S WAY PRIMADOPHILUS ORIGINAL ONCE DAILY TRUE POTENCY 5 BILLION CFU TRUE IDENTITY ACIDOPHILUS & RHAMNOSUS TRUE RELEASE TARGETED DELIVERY ENTERIC-COATED FOR ALL AGES |
| **1000903100** | PHILLIPS' COLON HEALTH PROBIOTIC CAPS BAYER |
| **1888324001** | DEFAULT PROBIOTIC |
| **1000949900** | ENZYMATIC THERAPY PROBIOTIC PEARLS HIGH POTENCY ADVANCED 3-LAYER SOFTGEL ONCE DAILY LACTOBACILLI & BIFIDOBACTERIA |
| **1000948600** | RENEW LIFE FLORABEAR FOR KIDS |
| **1000903100** | PHILLIPS' COLON HEALTH PROBIOTIC CAPS BAYER |
| **1000904300** | PROJOBA INTERNATIONAL PROBACILLUS PLUS |
| **1000617302** | RAINBOW LIGHT WOMEN'S ONE JUST 1 ONCE FOOD-BASED MULTIVITAMIN 800 IU VITAMIN D3 & PROBIOTICS |
| **1000980500** | KID'S KYO-DOPHILUS PROBIOTIC ONE PER DAY |
| **1000986900** | TRADER JOE'S ACTIVE 50+ ONCE DAILY MULTIVITAMIN & MINERAL PLUS PROBIOTICS 1000 IU VITAMIN D |
| **1888226201** | DEFAULT LACTOBACILLUS ACIDOPHILUS |
| **1888324001** | DEFAULT PROBIOTIC |
| **1000985700** | KLAIRE LABS SACCHAROMYCES BOULARDII 3+ BILLION CFUS PROBIOTIC |
| **1000986600** | THERALAC PROBIOTIC MASTER 5 HUMAN STRAINS 2 PREBIOTICS 5 + 2 30 BILLION CFU PER CAPSULE WITH LACTOSTIM |
| **1000522902** | NUTRITION NOW PB8 PRO-BIOTIC ACIDOPHILUS FOR LIFE ORIGINAL FORMULA 14 BILLION GOOD BACTERIA |
| **1000974100** | CULTURELLE DIGESTIVE HEALTH PROBIOTIC PROBIOTIC WITH NATURALLY SOURCED DAIRY-FREE LACTOBACILLUS GG ONCE DAILY CAPSULES 10 BILLION ACTIVE CULTURES |
| **1000962900** | NUTRILITE INTESTIFLORA-7 STICK PACKS |
| **1000880800** | FRIENDLY FLORA PREBIOTIC AND PROBIOTIC COMPLEX FOS AND LACTOBACILLUS 5 BILLION CFU PROCAPS LABORATORIES |
| **1000903600** | SOLGAR ADVANCED ACIDOPHILUS PLUS 500 MILLION MICROORGANISMS PER USE |
| **1000903100** | PHILLIPS' COLON HEALTH PROBIOTIC CAPS BAYER |
| **1888992400** | GENERIC36 ACIDOPHILUS PROBIOTIC BLEND |
| **1000972400** | NATURE'S WAY PRIMADOPHILUS ORIGINAL ONCE DAILY TRUE POTENCY 5 BILLION CFU TRUE IDENTITY ACIDOPHILUS & RHAMNOSUS TRUE RELEASE TARGETED DELIVERY ENTERIC-COATED FOR ALL AGES |
| **1000885701** | SPRING VALLEY PROBIOTIC ACIDOPHILUS 1 BILLION ACTIVE CULTURES |
| **1000916400** | ULTIMATE FLORA CRITICAL CARE 50 BILLION EXTRA-STRENGTH PROBIOTIC 10 PROBIOTIC STRAINS RENEW LIFE |
| **1000908900** | L'IL CRITTERS PROBIOTIC ACIDOPHILUS DAILY |
| **1000903100** | PHILLIPS' COLON HEALTH PROBIOTIC CAPS BAYER |
| **1000977200** | SUNDOWN NATURALS CHEWABLE ACIDOPHILUS WITH BIFIDUM 1 BILLION ACTIVE CULTURES PER WAFER VEGETARIAN FORMULA |
| **1888324001** | DEFAULT PROBIOTIC |
| **1000607400** | GARDEN OF LIFE PRIMAL DEFENSE HSO PROBIOTIC FORMULA |
| **1000949200** | NATURE'S BOUNTY CHEWABLE PROBIOTIC ACIDOPHILUS 1 BILLION ORGANISMS WITH BIFIDUM CHEWABLE MILK FREE WAFER |
| **1000973200** | PREMIER RESEARCH LABS GALLBLADDER-ND PROBIOTIC-DERIVED FORMULA FEATURING ND TECHNOLOGY |
| **1000918000** | FLORA UDO'S CHOICE UDO'S OIL DHA 3-6-9 BLEND BASED ON THE IDEAL 2:1:1 RATIO OF OMEGA FATTY ACIDS |
| **1000919300** | NEW CHAPTER ORGANICS PROBIOTIC ALL-FLORA WHOLE FOOD LIVE PROBIOTICS |
| **1000980500** | KID'S KYO-DOPHILUS PROBIOTIC ONE PER DAY |
| **1000725801** | RAINBOW LIGHT MEN'S ONE JUST 1 ONCE FOOD-BASED MULTIVITAMIN ENERGY B-COMPLEX & PROBIOTICS |
| **1000903100** | PHILLIPS' COLON HEALTH PROBIOTIC CAPS BAYER |
| **1000903100** | PHILLIPS' COLON HEALTH PROBIOTIC CAPS BAYER |
| **1001043500** | ULTIMATE FLORA KIDS PROBIOTIC 3 BILLION LIVE CULTURES PER TABLET PROBIOTIC RENEW LIFE |
| **1001057200** | FINEST NUTRITION ACIDOPHILUS L. ACIDOPHILUS 1 BILLION CFUS (LIVE ORGANISMS) PER TABLET ONE PER DAY |
| **1001087400** | JARROW FORMULAS JARRO-DOPHILUS + FOS 6 BENEFICIAL PROBIOTIC STRAINS WITH CLINICALLY DOCUMENTED STRAINS 3.4 BILLION PER CAPSULE PROBIOTIC |
| **1001064300** | DESIGNS FOR HEALTH PROBIOTIC SYNERGY POWDER 20 BILLION ORGANISMS PER SERVING |
| **1001027800** | GNC PROBIOTICS ULTRA 25 BILLION CFUS PROBIOTIC COMPLEX |
| **1001065100** | NATURE'S BLEND PROBIOTIC BLEND |
| **1001007200** | SUNDOWN NATURALS ULTIMATE PROBIOTIC FORMULA ACIDOPHILUS 2 BILLION ACTIVE CULTURES VEGETARIAN FORMULA 1 PER DAY |
| **1000910500** | SPRING VALLEY PROBIOTIC MULTI-ENZYME DIGESTIVE FORMULA 1 BILLION ACTIVE CULTURES PER SERVING |
| **1000617302** | RAINBOW LIGHT WOMEN'S ONE JUST 1 ONCE FOOD-BASED MULTIVITAMIN 800 IU VITAMIN D3 & PROBIOTICS |
| **1000366803** | NATURE'S WAY ONCE DAILY PRIMADOPHILUS CHILDREN POWDER WITH SCFOS TRUE IDENTITY BIFIDOBACTERIA & LACTOBACILLI TRUE POTENCY 3 BILLION CFU |
| **1000974100** | CULTURELLE DIGESTIVE HEALTH PROBIOTIC PROBIOTIC WITH NATURALLY SOURCED DAIRY-FREE LACTOBACILLUS GG ONCE DAILY CAPSULES 10 BILLION ACTIVE CULTURES |
| **1000628501** | NATURE'S WAY ONCE DAILY PRIMADOPHILUS KIDS TRUE POTENCY 3 BILLION CFU TRUE IDENTITY LACTOBACILLI & BIFIDOBACTERIA ORANGE AGES 2-12 |
| **1001052000** | GNC PROBIOTICS PROBIOTIC COMPLEX 4 4 BILLION CFUS |
| **1001065700** | BIOGAIA PROBIOTICS GERBER SOOTHE COLIC DROPS PROBIOTIC |
| **1001125100** | VSL#3 THE LIVING SHIELD 112.5 BILLION LIVE LACTIC ACID BACTERIA PER CAPSULE |
| **1000889101** | GARDEN OF LIFE RAW BEYOND VITAMINS & MINERALS VITAMIN CODE 50 & WISER MEN RAW WHOLE FOOD MULTI LIVE PROBIOTICS & ENZYMES RAW FOOD-CREATED NUTRIENTS WITH CODE FACTORS RAW FOOD |
| **1000885701** | SPRING VALLEY PROBIOTIC ACIDOPHILUS 1 BILLION ACTIVE CULTURES |
| **1001043500** | ULTIMATE FLORA KIDS PROBIOTIC 3 BILLION LIVE CULTURES PER TABLET PROBIOTIC RENEW LIFE |
| **1001113200** | DIGESTZEN PB ASSIST+ PROBIOTIC DEFENSE FORMULA DOTERRA |
| **1001083000** | 365 PROBIOTIC COMPLEX WITH ACIDOPHILUS DOUBLE STRENGTH |
| **1001043500** | ULTIMATE FLORA KIDS PROBIOTIC 3 BILLION LIVE CULTURES PER TABLET PROBIOTIC RENEW LIFE |
| **1000865500** | SPRING VALLEY SUPER STRENGTH PROBIOTIC ACIDOPHILUS 2 BILLION ACTIVE CULTURES |
| **1001049000** | METAGENICS ULTRAFLORA IMMUNE HEALTH PROBIOTIC |
| **1000916400** | ULTIMATE FLORA CRITICAL CARE 50 BILLION EXTRA-STRENGTH PROBIOTIC 10 PROBIOTIC STRAINS RENEW LIFE |
| **1001048300** | GNC ULTRA 50 BILLION CFUS PROBIOTIC COMPLEX GUARANTEED POTENCY 50 |
| **1001025500** | ULTIMATE FLORA EXTRA CARE DAILY PROBIOTIC 30 BILLION LIVE CULTURES PER CAPSULE 10 PROBIOTIC STRAINS ONE CAPSULE ONCE A DAY PROBIOTIC RENEW LIFE |
| **1000916400** | ULTIMATE FLORA CRITICAL CARE 50 BILLION EXTRA-STRENGTH PROBIOTIC 10 PROBIOTIC STRAINS RENEW LIFE |
| **1001075500** | KYO-DOPHILUS PROBIOTICS PLUS CRANBERRY EXTRACT |
| **1001013000** | SWANSON ULTRA PROBIOTIC COMPLEX |
| **1000593301** | TRADER JOE'S ACIDOPHILUS & PROBIOTIC COMPLEX 2 BILLION ORGANISMS PER TABLET |
| **1000951201** | SCHIFF DIGESTIVE ADVANTAGE PROBIOTIC GUMMIES |
| **1001013000** | SWANSON ULTRA PROBIOTIC COMPLEX |
| **1001034800** | NATURE'S BOUNTY ULTRA STRENGTH ADVANCED PROBIOTIC 10 10 PROBIOTIC ORGANISMS WITH ACTIVE CULTURE SUPPORT 20 BILLION LIVE PROBIOTIC CULTURES |
| **1000974100** | CULTURELLE DIGESTIVE HEALTH PROBIOTIC PROBIOTIC WITH NATURALLY SOURCED DAIRY-FREE LACTOBACILLUS GG ONCE DAILY CAPSULES 10 BILLION ACTIVE CULTURES |
| **1000889101** | GARDEN OF LIFE RAW BEYOND VITAMINS & MINERALS VITAMIN CODE 50 & WISER MEN RAW WHOLE FOOD MULTI LIVE PROBIOTICS & ENZYMES RAW FOOD-CREATED NUTRIENTS WITH CODE FACTORS RAW FOOD |
| **1881048201** | GENERIC7 PROBIOTIC ACIDOPHILUS |
| **1001087400** | JARROW FORMULAS JARRO-DOPHILUS + FOS 6 BENEFICIAL PROBIOTIC STRAINS WITH CLINICALLY DOCUMENTED STRAINS 3.4 BILLION PER CAPSULE PROBIOTIC |
| **1001106800** | THE HONEST CO. BABY & TODDLER MULTI POWDER COMPLETE MULTI-VITAMIN + MINERALS + SUPERFOODS + ANTIOXIDANTS + AMINO ACIDS + PROBIOTICS WHOLE-FOOD BASED ULTRA PURE GENTLE & ENRICHING HYPOALLERGENIC 100% N |
| **1000522902** | NUTRITION NOW PB8 PRO-BIOTIC ACIDOPHILUS FOR LIFE ORIGINAL FORMULA 14 BILLION GOOD BACTERIA |
| **1000949200** | NATURE'S BOUNTY CHEWABLE PROBIOTIC ACIDOPHILUS 1 BILLION ORGANISMS WITH BIFIDUM CHEWABLE MILK FREE WAFER |
| **1001097100** | 21ST CENTURY HIGH POTENCY ACIDOPHILUS |
| **1000903101** | PHILLIPS' COLON HEALTH PROBIOTIC CAPS DAILY PROBIOTIC ONE DAILY BAYER |
| **1000602001** | JARROW FORMULAS JARRO-DOPHILUS ORIGINAL WITH CLINICALLY DOCUMENTED STRAINS 6 BENEFICIAL PROBIOTIC STRAINS 3.4 BILLION PER CAPSULE PROBIOTIC |
| **1000903101** | PHILLIPS' COLON HEALTH PROBIOTIC CAPS DAILY PROBIOTIC ONE DAILY BAYER |
| **1888324001** | DEFAULT PROBIOTIC |
| **1001055500** | BIORAY CYTOFLORA PROBIOTIC IMMUNITY TONIC DAILY |
| **1000974100** | CULTURELLE DIGESTIVE HEALTH PROBIOTIC PROBIOTIC WITH NATURALLY SOURCED DAIRY-FREE LACTOBACILLUS GG ONCE DAILY CAPSULES 10 BILLION ACTIVE CULTURES |
| **1000530801** | NATURE MADE ACIDOPHILUS PROBIOTICS 1 BILLION LIVE CELLS PER SERVING |
| **1000999600** | NATURE'S BOUNTY ACIDOPHILUS PROBIOTIC 100 MILLION ORGANISMS LACTOBACILLUS ACIDOPHILUS 1 PER DAY TABLET |
| **1000910501** | SPRING VALLEY PROBIOTIC MULTI-ENZYME DIGESTIVE FORMULA WITH ACTIVE ACIDOPHILUS CULTURES |
| **1001106300** | HEALTHY ORIGINS NATURAL PROBIOTIC 30 BILLION CFU'S 8 STRAINS AND 30 BILLION COLONY FORMING UNITS FLORAFIT |
| **1000725802** | RAINBOW LIGHT MEN'S ONE JUST 1 ONCE FOOD-BASED MULTIVITAMIN ENERGY B-COMPLEX & PROBIOTICS |
| **1001034800** | NATURE'S BOUNTY ULTRA STRENGTH ADVANCED PROBIOTIC 10 10 PROBIOTIC ORGANISMS WITH ACTIVE CULTURE SUPPORT 20 BILLION LIVE PROBIOTIC CULTURES |
| **1001038700** | BASIC'S NATURAL ACIDOPHILUS BASIC VITAMINS |
| **1001034800** | NATURE'S BOUNTY ULTRA STRENGTH ADVANCED PROBIOTIC 10 10 PROBIOTIC ORGANISMS WITH ACTIVE CULTURE SUPPORT 20 BILLION LIVE PROBIOTIC CULTURES |
| **1001089000** | RITE AID PHARMACY NATURAL ACIDOPHILUS PROBIOTIC COMPLEX MILK FREE 300 MG |
| **1001090900** | MASON NATURAL CHEWABLE SOLUBLE FIBER WITH PROBIOTICS ADULTS & KIDS |
| **1000725802** | RAINBOW LIGHT MEN'S ONE JUST 1 ONCE FOOD-BASED MULTIVITAMIN ENERGY B-COMPLEX & PROBIOTICS |
| **1000999600** | NATURE'S BOUNTY ACIDOPHILUS PROBIOTIC 100 MILLION ORGANISMS LACTOBACILLUS ACIDOPHILUS 1 PER DAY TABLET |
| **1001077500** | TRUNATURE DIGESTIVE PROBIOTIC 10 BILLION ACTIVE CULTURES |
| **1001103200** | KLAIRE LABS THER-BIOTIC INFANT FORMULA 10+ BILLION CFUS MULTI-SPECIES PROBIOTIC |
| **1888226201** | DEFAULT LACTOBACILLUS ACIDOPHILUS |
| **1000251302** | KYO-DOPHILUS PROBIOTIC |
| **1001102300** | TOTAL PROBIOTICS NUTRI-WEST |
| **1000951201** | SCHIFF DIGESTIVE ADVANTAGE PROBIOTIC GUMMIES |
| **1000324000** | SHAKLEE BIFIDUS & ACIDOPHILUS OPTIFLORA PROBIOTIC COMPLEX |
| **1001007200** | SUNDOWN NATURALS ULTIMATE PROBIOTIC FORMULA ACIDOPHILUS 2 BILLION ACTIVE CULTURES VEGETARIAN FORMULA 1 PER DAY |
| **1000607400** | GARDEN OF LIFE PRIMAL DEFENSE HSO PROBIOTIC FORMULA |
| **1001062400** | NATURE MADE TRIPLE PROBIOTIC |
| **1001028400** | GNC PROBIOTIC COMPLEX CHEWABLE TABLET 1.5 BILLION CFUS |
| **1001122500** | HEALTHWAY MEDICAL, P.C. PRO-C 750 MG VITAMIN C WITH PROBIOTICS VEGETARIAN |
| **1000870200** | RAINBOW LIGHT 50+ MINI-TAB AGE-DEFENSE FORMULA FOOD-BASED MULTIVITAMIN WITH COQ10 1,000 IU VITAMIN D3 PROBIOTICS & DIGES |
| **1001154300** | FUSION PLUS IRON / FOLIC ACID / VITAMIN / PROBIOTIC CAPSULES |
| **1888324001** | DEFAULT PROBIOTIC |
| **1000840100** | GARDEN OF LIFE PRIMAL DEFENSE ULTRA ULTIMATE PROBIOTIC FORMULA 15 BILLION CELLS DAILY 13 BENEFICIAL CULTURES WITH HSOS W |
| **1000951201** | SCHIFF DIGESTIVE ADVANTAGE PROBIOTIC GUMMIES |
| **1000916400** | ULTIMATE FLORA CRITICAL CARE 50 BILLION EXTRA-STRENGTH PROBIOTIC 10 PROBIOTIC STRAINS RENEW LIFE |
| **1000999600** | NATURE'S BOUNTY ACIDOPHILUS PROBIOTIC 100 MILLION ORGANISMS LACTOBACILLUS ACIDOPHILUS 1 PER DAY TABLET |
| **1000830701** | ALIGN PROBIOTIC B. INFANTIS 35624 WITH: UNIQUE B. INFANTIS 35624 |
| **1000903101** | PHILLIPS' COLON HEALTH PROBIOTIC CAPS DAILY PROBIOTIC ONE DAILY BAYER |
| **1000885701** | SPRING VALLEY PROBIOTIC ACIDOPHILUS 1 BILLION ACTIVE CULTURES |
| **1000951201** | SCHIFF DIGESTIVE ADVANTAGE PROBIOTIC GUMMIES |
| **1000324101** | SHAKLEE OPTIFLORA PREBIOTIC COMPLEX FOS, INULIN, & MORE |
| **1001108700** | CELL NUTRITIONALS PROBIOTICS AR ACID RESISTENT 16 STRAIN FORMULA COMPREHENSIVE, DAIRY FREE FORMULA PROVIDING 12 BILLION VIABLE CELLS PER CAPSULE AT TIME OF MANUFACTURE |
| **1001026200** | COUNTRY LIFE DAIRY-FREE ACIDOPHILUS WITH PECTIN |
| **1001043500** | ULTIMATE FLORA KIDS PROBIOTIC 3 BILLION LIVE CULTURES PER TABLET PROBIOTIC RENEW LIFE |
| **1000903101** | PHILLIPS' COLON HEALTH PROBIOTIC CAPS DAILY PROBIOTIC ONE DAILY BAYER |
| **1001077500** | TRUNATURE DIGESTIVE PROBIOTIC 10 BILLION ACTIVE CULTURES |
| **1000964100** | JUICEFESTIV FRUITFESTIV 23 FRUITS, ANTIOXIDANTS, PROBIOTICS & DIGESTIVE ENZYMES FEATURES: ACAI, POMEGRANATE, BLUEBERRY AND NONI NATROL |
| **1000903101** | PHILLIPS' COLON HEALTH PROBIOTIC CAPS DAILY PROBIOTIC ONE DAILY BAYER |
| **1000242002** | AMERICAN HEALTH CHEWABLE MILK FREE ACIDOPHILUS AND BIFIDUM ONE BILLION ORGANISMS VEGETARIAN FORMULA |
| **1001103300** | FLORABABY ADVANCED PROBIOTIC FORMULA FOR INFANTS & TODDLERS 5 STRAINS OF BENEFICIAL PROBIOTICS 4 BILLION LIVE CULTURES PER SERVING PROBIOTIC RENEW LIFE |
| **1001110200** | TRUBIOTICS DAILY PROBIOTIC FROM THE MAKERS OF ONE A DAY BAYER |
| **1001085400** | ACCUFLORA ADVANCED CD PROBIOTIC 5 PROBIOTIC STRAINS ADVANCED CD: CONTROLLED DELIVERY |
| **1001089000** | RITE AID PHARMACY NATURAL ACIDOPHILUS PROBIOTIC COMPLEX MILK FREE 300 MG |
| **1000774001** | NATURE'S BOUNTY PROBIOTIC ACIDOPHILUS 100 MILLION ORGANISMS LACTOBACILLUS ACIDOPHILUS 1 PER DAY CAPSULE |
| **1000830701** | ALIGN PROBIOTIC B. INFANTIS 35624 WITH: UNIQUE B. INFANTIS 35624 |
| **1000838601** | NEW VITALITY RUBY REDS NEW & IMPROVED FORMULA POMEGRANATE, ACAI & MAQUI A DELICIOUS FRUIT AND VEGETABLE WITH POTENT VITAMINS, MINERALS, ENZYMES, HERBS, NUTRIENTS AND PROBIOTICS 100% DAILY VALUE 7 VITA |
| **1001162800** | MULTI-PROBIOTIC 4000 DOUGLAS LABORATORIES |
| **1000617303** | RAINBOW LIGHT WOMEN'S ONE FOOD-BASED MULTIVITAMIN EASY TO DIGEST WITH PROBIOTICS & ENZYMES |
| **1001240000** | RENEW LIFE EVERYDAY ULTIMATE FLORA PROBIOTIC 15 BILLION LIVE CULTURES PER CAPSULE 10 PROBIOTIC STRAINS PROBIOTIC |
| **1001198000** | ISOTONIX DIGESTIVE ENZYMES WITH PROBIOTICS |
| **1000999601** | NATURE'S BOUNTY ACIDOPHILUS PROBIOTIC 100 MILLION ORGANISMS |
| **1001273500** | BLUE ROCK HOLISTICS SUPER SHIELD SUPREME MULTI-STRAIN ADULT PROBIOTIC FORMULA |
| **1001184600** | DR. MERCOLA COMPLETE PROBIOTICS 70 BILLION CFU ACID AND BILE RESISTANT 10 STRAINS |
| **1001274100** | PROBIOTIC AMERICA PERFECT BIOTICS 30 BILLION CFUS 15 STRAINS ROOM TEMP STABLE DAILY PROBIOTIC |
| **1000530801** | NATURE MADE ACIDOPHILUS PROBIOTICS 1 BILLION LIVE CELLS PER SERVING |
| **1001176301** | PURITAN'S PRIDE PREMIUM PROBIOTIC 10 20 BILLION LIVE PROBIOTIC CULTURES WITH CLINICALLY STUDIED STRAINS |
| **1001213400** | YOUNG LIVING LIFE 5 HIGH-POTENCY PROBIOTIC 10 BILLION ACTIVE CULTURES |
| **1001224700** | CULTURELLE KIDS CHEWABLES DAILY PROBIOTIC FORMULA 5 BILLION ACTIVE CULTURES! PROBIOTIC WITH NATURALLY SOURCED LACTOBACILLUS GG |
| **1880999601** | DEFAULT LACTOBACILLUS ACIDOPHILUS |
| **1000999601** | NATURE'S BOUNTY ACIDOPHILUS PROBIOTIC 100 MILLION ORGANISMS |
| **1001201700** | GENESTRA BRANDS HMF NEURO POWDER PROBIOTIC |
| **1000999601** | NATURE'S BOUNTY ACIDOPHILUS PROBIOTIC 100 MILLION ORGANISMS |
| **1001224700** | CULTURELLE KIDS CHEWABLES DAILY PROBIOTIC FORMULA 5 BILLION ACTIVE CULTURES! PROBIOTIC WITH NATURALLY SOURCED LACTOBACILLUS GG |
| **1001257100** | PURITAN'S PRIDE ACIDOPHILUS COMPLEX CONTAINS: L. ACIDOPHILUS, B. BIFIDUM 1 BILLION ACTIVE CULTURES |
| **1001184600** | DR. MERCOLA COMPLETE PROBIOTICS 70 BILLION CFU ACID AND BILE RESISTANT 10 STRAINS |
| **1001180800** | SWANSON PROBIOTICS DR. STEPHEN LANGER'S ULTIMATE 16 STRAIN PROBIOTIC WITH TRACE MINERALS & FOS 3 BILLION CFU VEGETARIAN FORMULA |
| **1000903101** | PHILLIPS' COLON HEALTH PROBIOTIC CAPS DAILY PROBIOTIC ONE DAILY BAYER |
| **1001257300** | PURITAN'S PRIDE PROBIOTIC ACIDOPHILUS STOMACH FRIENDLY 100 MILLION ACTIVE CULTURES VEGETARIAN |
| **1001187100** | KROGER PROBIOTIC COLON SUPPORT WITH BIO-RELEASE 4 STRAINS OF GOOD BACTERIA ONCE DAILY |
| **1001034801** | NATURE'S BOUNTY ULTRA STRENGTH PROBIOTIC 10 20 BILLION LIVE PROBIOTIC CULTURES 10 PROBIOTIC STRAINS |
| **1001147400** | DIGESTIVE ADVANTAGE KIDS DAILY PROBIOTIC GUMMIES |
| **1001176300** | PURITAN'S PRIDE PROBIOTIC 10 FROM 10 DIFFERENT PROBIOTIC ORGANISMS 20 BILLION ACTIVE CULTURES PER SERVING |
| **1001240000** | RENEW LIFE EVERYDAY ULTIMATE FLORA PROBIOTIC 15 BILLION LIVE CULTURES PER CAPSULE 10 PROBIOTIC STRAINS PROBIOTIC |
| **1000885702** | SPRING VALLEY PROBIOTIC ACIDOPHILUS 1 BILLION ACTIVE CULTURES PER CAPLET |
| **1001243700** | GARDEN OF LIFE DR. FORMULATED PROBIOTICS ONCE DAILY MEN'S 50 BILLION GUARANTEED 15 PROBIOTIC STRAINS RAW PROBIOTIC |
| **1000999601** | NATURE'S BOUNTY ACIDOPHILUS PROBIOTIC 100 MILLION ORGANISMS |
| **1001077500** | TRUNATURE DIGESTIVE PROBIOTIC 10 BILLION ACTIVE CULTURES |
| **1000903101** | PHILLIPS' COLON HEALTH PROBIOTIC CAPS DAILY PROBIOTIC ONE DAILY BAYER |
| **1001180900** | LEE SWANSON SIGNATURE LINE ULTIMATE PROBIOTIC FORMULA OVER 66 BILLION ORGANISMS PER CAPSULE |
| **1001160300** | BIOTRUST PRO-X10 EQUIVALENT TO 50 BILLION CFU PER DAY! NOW WITH PREFORPRO ADVANCED PROBIOTIC AND GI HEALTH FORMULA |
| **1000993201** | CULTURELLE KIDS PACKETS DAILY PROBIOTIC FORMULA 5 BILLION ACTIVE CULTURES! PROBIOTIC WITH NATURALLY SOURCED LACTOBACILLUS GG |
| **1001207200** | GARDEN OF LIFE DR. FORMULATED PROBIOTICS ONCE DAILY ULTRA 90 BILLION GUARANTEED 15 PROBIOTIC STRAINS RAW PROBIOTIC |
| **1001238100** | FLORA UDO'S CHOICE UDO'S OIL 3-6-9 BLEND BASED ON THE IDEAL 2:1:1 RATIO OF OMEGA FATTY ACIDS MADE WITH FLAX, SESAME, SUNFLOWER, AND OTHER SEED OILS |
| **1000999601** | NATURE'S BOUNTY ACIDOPHILUS PROBIOTIC 100 MILLION ORGANISMS |
| **1001263000** | BODY DYNAMICS MAXZORB NUTRITION TOTAL DIGESTION PROBIOTIC (MULTISTRAIN WITH) DIGESTIVE ENZYMES 50 BILLION CFU/G |
| **1001034801** | NATURE'S BOUNTY ULTRA STRENGTH PROBIOTIC 10 20 BILLION LIVE PROBIOTIC CULTURES 10 PROBIOTIC STRAINS |
| **1000993201** | CULTURELLE KIDS PACKETS DAILY PROBIOTIC FORMULA 5 BILLION ACTIVE CULTURES! PROBIOTIC WITH NATURALLY SOURCED LACTOBACILLUS GG |
| **1000522903** | PB8 ORIGINAL FORMULA WITH LACTOBACILLUS & BIFIDOBACTERIUM SPECIES |
| **1000986601** | THERALAC BIO-REPLENISHING PROBIOTIC 5 COLONIZING STRAINS 2 PREBIOTICS 5+2 WITH LACTOSTIM 30 BILLION COLONIZING CFU/CAPSULE MASTER SUPPLEMENTS |
| **1001034801** | NATURE'S BOUNTY ULTRA STRENGTH PROBIOTIC 10 20 BILLION LIVE PROBIOTIC CULTURES 10 PROBIOTIC STRAINS |
| **1001162200** | COUNTRY LIFE DAIRY-FREE PROBIOTIC POWER-DOPHILUS MULTI-STRAIN FORMULA 12 BILLION CFUS PER SERVING (AT THE TIME OF MANUFACTURE) |
| **1000885702** | SPRING VALLEY PROBIOTIC ACIDOPHILUS 1 BILLION ACTIVE CULTURES PER CAPLET |
| **1001243800** | GARDEN OF LIFE DR. FORMULATED PROBIOTICS ORGANIC KIDS+ PROBIOTICS + VITAMINS C & D 5 BILLION GUARANTEED 14 PROBIOTIC STRAINS USDA ORGANIC NO SUGAR ADDED PROBIOTIC |
| **1000672002** | IVL INSTITUTE FOR VIBRANT LIVING GO RUBY GO! THE ULTIMATE SUPER FOOD FRUIT DRINK CONTAINS PROBIOTICS |
| **1000488704** | RAINBOW LIGHT PRENATAL ONE FOOD-BASED MULTIVITAMIN EASY TO DIGEST WITH PROBIOTICS & ENZYMES |
| **1001218300** | OLLY KIDS HAPPY TUMMY PROBIOTIC, PREBIOTIC & PEPPERMINT |
| **1001241700** | FINEST NUTRITION DIGESTIVE PROBIOTIC MAXIMUM CARE 30 BILLION ACTIVE CULTURES PER CAPSULE ONE PER DAY |
| **1000830702** | ALIGN PROBIOTIC CLINICALLY STUDIED B. INFANTIS 35624 |
| **1000617303** | RAINBOW LIGHT WOMEN'S ONE FOOD-BASED MULTIVITAMIN EASY TO DIGEST WITH PROBIOTICS & ENZYMES |
| **1001261400** | ADVOCARE PROBIOTIC RESTORE ULTRA PROBIOTIC |
| **1001147400** | DIGESTIVE ADVANTAGE KIDS DAILY PROBIOTIC GUMMIES |
| **1000242003** | AMERICAN HEALTH CHEWABLE ACIDOPHILUS AND BIFIDUM ONE BILLION ORGANISMS VEGETARIAN FORMULA |
| **1001211100** | METAGENICS ULTRAFLORA CHILDREN'S DAILY PROBIOTIC CHEWABLE TABLETS |
| **1000522903** | PB8 ORIGINAL FORMULA WITH LACTOBACILLUS & BIFIDOBACTERIUM SPECIES |
| **1001223100** | NATURE'S WAY PRIMADOPHILUS FORTIFY DAILY PROBIOTIC 30 BILLION LIVE PROBIOTIC CULTURES PER CAPSULE DELAYED RELEASE PROBIOTIC |
| **1001231200** | CVS HEALTH PROBIOTIC FORMULA ACIDOPHILUS 1 BILLION ACTIVE CULTURES |
| **1001180900** | LEE SWANSON SIGNATURE LINE ULTIMATE PROBIOTIC FORMULA OVER 66 BILLION ORGANISMS PER CAPSULE |
| **1001224700** | CULTURELLE KIDS CHEWABLES DAILY PROBIOTIC FORMULA 5 BILLION ACTIVE CULTURES! PROBIOTIC WITH NATURALLY SOURCED LACTOBACILLUS GG |
| **1001207400** | FUTUREBIOTICS LONGEST LIVING ACIDOPHILUS+ OVER 1.5 BILLION DAIRY-FREE ORGANISMS PER CAPSULE NOW 100% VEGETARIAN |
| **1000830702** | ALIGN PROBIOTIC CLINICALLY STUDIED B. INFANTIS 35624 |
| **1000242003** | AMERICAN HEALTH CHEWABLE ACIDOPHILUS AND BIFIDUM ONE BILLION ORGANISMS VEGETARIAN FORMULA |
| **1000593301** | TRADER JOE'S ACIDOPHILUS & PROBIOTIC COMPLEX 2 BILLION ORGANISMS PER TABLET |
| **1001034801** | NATURE'S BOUNTY ULTRA STRENGTH PROBIOTIC 10 20 BILLION LIVE PROBIOTIC CULTURES 10 PROBIOTIC STRAINS |
| **1000516601** | BARLEAN'S GREENS ORGANIC GREEN FOOD CONCENTRATES GREEN PROTEIN SOURCE PLANT-BASED VITAMINS & MINERALS VEGETARIAN FORMULA HERBAL ANTIOXIDANTS HERBAL TONICS FLAX LIGNANS PROBIOTICS & ENZYMES |
| **1000607401** | GARDEN OF LIFE PRIMAL DEFENSE HSO PROBIOTIC FORMULA VEGETARIAN WHOLE FOOD |
| **1001224900** | HYPERBIOTICS PRO-KIDS THE PERFECT CHILDREN'S PROBIOTIC 3 BILLION CFU ONE VERY TINY PEARL PATENTED, TIME-RELEASE, MICRO-PEARLS 100% VEGETARIAN |
| **1001275700** | FLORASTOR DAILY PROBIOTIC UNIQUE STRAIN WITH LARGER, STRONGER CELLS THAN OTHER PROBIOTICS SACCHAROMYCES BOULARDII LYO CNCM I-745 250 MG |
| **1000617303** | RAINBOW LIGHT WOMEN'S ONE FOOD-BASED MULTIVITAMIN EASY TO DIGEST WITH PROBIOTICS & ENZYMES |
| **1000993201** | CULTURELLE KIDS PACKETS DAILY PROBIOTIC FORMULA 5 BILLION ACTIVE CULTURES! PROBIOTIC WITH NATURALLY SOURCED LACTOBACILLUS GG |
| **1880903101** | GENERIC PROBIOTIC COLON SUPPORT |
| **1001202300** | KLAIRE LABS LACTOPRIME PLUS SCD COMPLIANT 25+ BILLION CFUS MULTI-SPECIES PROBIOTIC |
| **1000951202** | SCHIFF DIGESTIVE ADVANTAGE PROBIOTIC GUMMIES POWERED BY GANEDEN BC30 PROBIOTIC |
| **1001239800** | RENEW LIFE ULTIMATE FLORA PROBIOTIC GUMMIES 2 BILLION LIVE CULTURES PER SERVING FOR KIDS AGES 2-102 PROBIOTIC |
| **1001007201** | SUNDOWN NATURALS ULTIMATE PROBIOTIC FORMULA ACIDOPHILUS 2 BILLION ACTIVE CULTURES VEGETARIAN FORMULA 1 PER DAY |
| **1000324000** | SHAKLEE BIFIDUS & ACIDOPHILUS OPTIFLORA PROBIOTIC COMPLEX |
| **1000324101** | SHAKLEE OPTIFLORA PREBIOTIC COMPLEX FOS, INULIN, & MORE |
| **1001177400** | JARROW FORMULAS JARRO-DOPHILUS EPS ENHANCED PROBIOTIC SYSTEM WITH CLINICALLY DOCUMENTED STRAINS ROOM TEMPERATURE STABLE 5 BILLION PER CAPSULE 8 STRAINS ENTEROGUARD PROBIOTIC |
| **1001257700** | REXALL PROBIOTIC ACIDOPHILUS 100 MILLION ACTIVE CULTURES |
| **1001263400** | CUSTOM PROBIOTICS HIGH COUNT, MULTI-STRAIN ACIDOPHILUS AND BIFIDUS 60 BILLION CFU'S/CAPSULE ADULT FORMULA CP-1 DAIRY FREE |
| **1880999601** | DEFAULT LACTOBACILLUS ACIDOPHILUS |
| **1001034801** | NATURE'S BOUNTY ULTRA STRENGTH PROBIOTIC 10 20 BILLION LIVE PROBIOTIC CULTURES 10 PROBIOTIC STRAINS |
| **1001113200** | DIGESTZEN PB ASSIST+ PROBIOTIC DEFENSE FORMULA DOTERRA |
| **1000964101** | NATROL JUICEFESTIV DAILY FRUIT 23 FRUITS +ANTIOXIDANTS, PROBIOTICS & DIGESTIVE ENZYMES MADE WITH ORGANIC FRUITS |
| **1001194200** | MEMBER'S MARK ULTIMATE PROBIOTIC FORMULA ACIDOPHILUS MADE WITH BIOFLORA 2 BILLION ACTIVE CULTURES 1 CAPLET PER DAY |
| **1888324001** | DEFAULT PROBIOTIC |
| **1001239900** | RENEW LIFE ULTIMATE FLORA PROBIOTIC FIZZY DRINK MIX 15 BILLION LIVE CULTURES PER SERVING PROBIOTIC |
| **1001257200** | PURITAN'S PRIDE PREMIUM PROBIOTIC ACIDOPHILUS 100 MILLION ACTIVE CULTURES |
| **1000522903** | PB8 ORIGINAL FORMULA WITH LACTOBACILLUS & BIFIDOBACTERIUM SPECIES |
| **1000908400** | REPHRESH PRO-B PROBIOTIC FEMININE |
| **1001205500** | DR. WHITAKER PEARLBIOTIC SPHERES DELIVERS 2 BILLION LIVE PROBIOTICS IN 7 STRAINS |
| **1881264601** | GENERIC48 ALL NATURAL ACIDOPHILUS HIGH POTENCY |
| **1000798801** | FLORASTORKIDS DAILY PROBIOTIC UNIQUE STRAIN WITH LARGER, STRONGER CELLS THAN OTHER PROBIOTICS SACCHAROMYCES BOULARDII LYO CNCM I-745 SACHETS \| 250 MG \| POWDER |
| **1001110200** | TRUBIOTICS DAILY PROBIOTIC FROM THE MAKERS OF ONE A DAY BAYER |
| **1001270700** | GARDEN OF LIFE RAW PROBIOTICS WOMEN 85 BILLION LIVE CULTURES 32 PROBIOTIC STRAINS PROBIOTIC-CREATED VITAMINS, MINERALS, ENZYMES & PREBIOTICS CONTAINS TARGETED PROBIOTICS RAW WHOLE FOOD |
| **1001229400** | LEAF ORIGIN ESSENTIAL CULTURES DAILY PROBIOTIC 30 BILLION CFU 15 UNIQUE STRAINS |
| **1001201500** | SCHIFF DIGESTIVE ADVANTAGE DAILY PROBIOTIC POWERED BY GANEDEN BC30 PROBIOTIC |
| **1001224700** | CULTURELLE KIDS CHEWABLES DAILY PROBIOTIC FORMULA 5 BILLION ACTIVE CULTURES! PROBIOTIC WITH NATURALLY SOURCED LACTOBACILLUS GG |
| **1001125100** | VSL#3 THE LIVING SHIELD 112.5 BILLION LIVE LACTIC ACID BACTERIA PER CAPSULE |
| **1001147400** | DIGESTIVE ADVANTAGE KIDS DAILY PROBIOTIC GUMMIES |
| **1000903101** | PHILLIPS' COLON HEALTH PROBIOTIC CAPS DAILY PROBIOTIC ONE DAILY BAYER |
| **1000951202** | SCHIFF DIGESTIVE ADVANTAGE PROBIOTIC GUMMIES POWERED BY GANEDEN BC30 PROBIOTIC |
| **1000854301** | VIBRANT HEALTH GREEN VIBRANCE PLANT-BASED ADVANCED DAILY SUPERFOOD +25 BILLION PROBIOTICS CLINICALLY FORMULATED \| VERSION 15.0 |
| **1001248800** | NUCIFIC BIO-X4 4-IN-1 WEIGHT MANAGEMENT PROBIOTIC |
| **1000617303** | RAINBOW LIGHT WOMEN'S ONE FOOD-BASED MULTIVITAMIN EASY TO DIGEST WITH PROBIOTICS & ENZYMES |
| **1000661201** | NATURE'S WAY PRIMADOPHILUS BIFIDUS ONCE DAILY TRUE POTENCY 5 BILLION CFU FOR ADULTS TRUE IDENTITY BIFIDOBACTERIA & LACTOBACILLI TRUE RELEASE TARGETED DELIVERY ENTERIC-COATED |
| **1001240000** | RENEW LIFE EVERYDAY ULTIMATE FLORA PROBIOTIC 15 BILLION LIVE CULTURES PER CAPSULE 10 PROBIOTIC STRAINS PROBIOTIC |
| **1001268500** | COUNTRY LIFE CORE DAILY-1 MULTIVITAMINS 1 DAILY WOMEN 50+ ONE TABLET INCLUDES: 200+ MG OF WOMEN'S HEALTH BLEND OVER 30 RAW WHOLE FOODS 'COENZYMATED' B VITAMINS AND PROBIOTICS, DIGESTIVE ENZYMES |
| **1001222600** | OLLY KIDS' MULTI + PROBIOTIC A BLEND OF VITAMINS A, C, D, E, BS & ZINC |
| **1001267500** | BIOGAIA PROTECTIS DROPS WITH VITAMIN D PROBIOTIC |
| **1000488704** | RAINBOW LIGHT PRENATAL ONE FOOD-BASED MULTIVITAMIN EASY TO DIGEST WITH PROBIOTICS & ENZYMES |
| **1001147400** | DIGESTIVE ADVANTAGE KIDS DAILY PROBIOTIC GUMMIES |
| **1001243900** | GARDEN OF LIFE DR. FORMULATED PROBIOTICS URINARY TRACT+ 50 BILLION GUARANTEED 16 PROBIOTIC STRAINS MADE WITH ORGANIC CRANBERRY & ACACIA FIBER RAW PROBIOTIC |
| **1000711401** | NATURE'S BLEND HIGH POTENCY CHEWABLE ACIDOPHILUS |
| **1000916401** | ULTIMATE FLORA CRITICAL CARE 50 BILLION LIVE CULTURES PER CAPSULE 10 PROBIOTIC STRAINS DELAYED RELEASE CAPSULES PROBIOTIC RENEW LIFE |
| **1000242003** | AMERICAN HEALTH CHEWABLE ACIDOPHILUS AND BIFIDUM ONE BILLION ORGANISMS VEGETARIAN FORMULA |
| **1001251800** | VIBRANT HEALTH GREEN VIBRANCE JUNIOR FOR TODDLERS + TWEENS VERSION 4.0 9 BILLION PROBIOTICS PER DOSE FROM 9 STRAINS GREEN SUPERFOOD + PROTEIN + D3 SPECIFICALLY DESIGNED FOR A GROWING CHILD |
| **1001263500** | CULTURELLE PROBIOTICS DIGESTIVE HEALTH DAILY PROBIOTIC WITH 100% LACTOBACILLUS GG 10 BILLION ACTIVE CULTURES CHEWABLES ONCE DAILY TABLETS |
| **1001279800** | NUTRILITE KIDS SEASONAL STRENGTH PROBIOTIC |
| **1001258600** | INNATE RESPONSE FORMULAS FLORA 20-14 ULTRA STRENGTH 14 NON-COMPETING STRAINS 20 BILLION ACTIVE PROBIOTIC CELLS |
| **1001222600** | OLLY KIDS' MULTI + PROBIOTIC A BLEND OF VITAMINS A, C, D, E, BS & ZINC |
| **1000999601** | NATURE'S BOUNTY ACIDOPHILUS PROBIOTIC 100 MILLION ORGANISMS |
| **1000319802** | SUNDOWN NATURALS PROBIOTIC ACIDOPHILUS XTRA 40 MILLION LIVE CELLS PER SERVING VEGETARIAN FORMULA |
| **1001263500** | CULTURELLE PROBIOTICS DIGESTIVE HEALTH DAILY PROBIOTIC WITH 100% LACTOBACILLUS GG 10 BILLION ACTIVE CULTURES CHEWABLES ONCE DAILY TABLETS |
| **1000617303** | RAINBOW LIGHT WOMEN'S ONE FOOD-BASED MULTIVITAMIN EASY TO DIGEST WITH PROBIOTICS & ENZYMES |
| **1001248800** | NUCIFIC BIO-X4 4-IN-1 WEIGHT MANAGEMENT PROBIOTIC |
| **1000903101** | PHILLIPS' COLON HEALTH PROBIOTIC CAPS DAILY PROBIOTIC ONE DAILY BAYER |
| **1000242003** | AMERICAN HEALTH CHEWABLE ACIDOPHILUS AND BIFIDUM ONE BILLION ORGANISMS VEGETARIAN FORMULA |
| **1001214500** | DR. SINATRA HEART HEALTHY PROBIOTIC SOLUTIONS |
| **1000530801** | NATURE MADE ACIDOPHILUS PROBIOTICS 1 BILLION LIVE CELLS PER SERVING |
| **1001165200** | FLORA UDO'S CHOICE SUPER 5 LOZENGE PROBIOTIC 2 BILLION CELLS AT TIME OF MANUFACTURE |
| **1001222600** | OLLY KIDS' MULTI + PROBIOTIC A BLEND OF VITAMINS A, C, D, E, BS & ZINC |
| **1000949201** | NATURE'S BOUNTY CHEWABLE PROBIOTIC ACIDOPHILUS 1 BILLION ORGANISMS WITH LACTIS CHEWABLE MILK FREE WAFER |
| **1001085400** | ACCUFLORA ADVANCED CD PROBIOTIC 5 PROBIOTIC STRAINS ADVANCED CD: CONTROLLED DELIVERY |
| **1001207100** | GARDEN OF LIFE DR. FORMULATED PROBIOTICS MOOD+ 50 BILLION GUARANTEED 16 PROBIOTIC STRAINS MADE WITH ORGANIC ASHWAGANDHA, BLUEBERRIES & ACACIA FIBER RAW PROBIOTIC |
| **1001236100** | UP4 PROBIOTICS ADULT PROBIOTIC 15 BILLION CFU GUARANTEED DDS-1 TRADEMARKED SUPERSTRAIN |
| **1000617303** | RAINBOW LIGHT WOMEN'S ONE FOOD-BASED MULTIVITAMIN EASY TO DIGEST WITH PROBIOTICS & ENZYMES |
| **1000242003** | AMERICAN HEALTH CHEWABLE ACIDOPHILUS AND BIFIDUM ONE BILLION ORGANISMS VEGETARIAN FORMULA |
| **1000324000** | SHAKLEE BIFIDUS & ACIDOPHILUS OPTIFLORA PROBIOTIC COMPLEX |
| **1000324101** | SHAKLEE OPTIFLORA PREBIOTIC COMPLEX FOS, INULIN, & MORE |
| **1000993201** | CULTURELLE KIDS PACKETS DAILY PROBIOTIC FORMULA 5 BILLION ACTIVE CULTURES! PROBIOTIC WITH NATURALLY SOURCED LACTOBACILLUS GG |
| **1001062400** | NATURE MADE TRIPLE PROBIOTIC |
| **1000425301** | FLORADIX KINDER LOVE CHILDREN'S MULTIVITAMIN LIQUID HERBAL EXTRACT FORMULA RICH IN VITAMINS A, B, C, D & E GOOD SOURCE OF CALCIUM HERBAL EXTRACTS AND FRUIT JUICES VEGETARIAN LIQUID FORMULA |
| **1001201700** | GENESTRA BRANDS HMF NEURO POWDER PROBIOTIC |
| **1001284900** | SUNIBIOTICS POTENT PROBIOTICS WITH ORGANIC PREBIOTICS POWDER SUPERFOOD PROBIOTIC 20 BILLION CELLS PER SERVING NEW FORMULA DAILY PROBIOTIC USDA ORGANIC |
| **1000974101** | CULTURELLE DIGESTIVE HEALTH DAILY PROBIOTIC FORMULA NOW 100% VEGETARIAN ONCE DAILY VEGETARIAN CAPSULES |
| **1000628502** | NATURE'S WAY ONCE DAILY PRIMADOPHILUS KIDS TRUE POTENCY 3 BILLION CFU TRUE IDENTITY LACTOBACILLI & BIFIDOBACTERIA AGES 2-12 |
| **1000999601** | NATURE'S BOUNTY ACIDOPHILUS PROBIOTIC 100 MILLION ORGANISMS |
| **1001180000** | ULTIMATE FLORA MEN'S COMPLETE 90 BILLION LIVE CULTURES PER CAPSULE 14 GPS PROBIOTIC STRAINS RENEW LIFE DELAYED RELEASE CAPSULES PROBIOTIC |
| **1001147400** | DIGESTIVE ADVANTAGE KIDS DAILY PROBIOTIC GUMMIES |
| **1000522903** | PB8 ORIGINAL FORMULA WITH LACTOBACILLUS & BIFIDOBACTERIUM SPECIES |
| **1000854301** | VIBRANT HEALTH GREEN VIBRANCE PLANT-BASED ADVANCED DAILY SUPERFOOD +25 BILLION PROBIOTICS CLINICALLY FORMULATED \| VERSION 15.0 |
| **1888324001** | DEFAULT PROBIOTIC |
| **1000488704** | RAINBOW LIGHT PRENATAL ONE FOOD-BASED MULTIVITAMIN EASY TO DIGEST WITH PROBIOTICS & ENZYMES |
| **1001192000** | PROBIOTIC 14 UNIQUE STRAINS 500 MG |
| **1888324001** | DEFAULT PROBIOTIC |
| **1001279200** | BACTICURE NATURAL PROBIOTIC ORGANIC PRODUCT |
| **1000854301** | VIBRANT HEALTH GREEN VIBRANCE PLANT-BASED ADVANCED DAILY SUPERFOOD +25 BILLION PROBIOTICS CLINICALLY FORMULATED \| VERSION 15.0 |
| **1001077500** | TRUNATURE DIGESTIVE PROBIOTIC 10 BILLION ACTIVE CULTURES |
| **1001240000** | RENEW LIFE EVERYDAY ULTIMATE FLORA PROBIOTIC 15 BILLION LIVE CULTURES PER CAPSULE 10 PROBIOTIC STRAINS PROBIOTIC |
| **1001201900** | SEEKING HEALTH PROBIOTA SENSITIVE SCD AND GAPS COMPLIANT PROBIOTIC PHYSICIAN FORMULATED |
| **1001190800** | MEMBER'S MARK 5X PROBIOTIC FORMULATED WITH 5 STRAINS OF HEALTHY BACTERIA INCLUDING B. INFANTIS |
| **1001187600** | NATURE'S BOUNTY PROBIOTIC GUMMIES 4 BILLION LIVE CULTURES PER SERVING CONTAINS PROBIOTIC STRAIN BACILLUS COAGULANS UNIQUE IS-2 |
| **1001227700** | MEGAFOOD KIDS N' US MEGAFLORA 5 BILLION CFU PROBIOTIC |
| **1001085400** | ACCUFLORA ADVANCED CD PROBIOTIC 5 PROBIOTIC STRAINS ADVANCED CD: CONTROLLED DELIVERY |
| **1888324001** | DEFAULT PROBIOTIC |
| **1888324001** | DEFAULT PROBIOTIC |
| **1001182900** | 365 DAILY PROBIOTIC WITH ACIDOPHILUS 4 BILLION CULTURES 4 IMPORTANT PROBIOTIC STRAINS PER TABLET |
| **1001213400** | YOUNG LIVING LIFE 5 HIGH-POTENCY PROBIOTIC 10 BILLION ACTIVE CULTURES |
| **1001251800** | VIBRANT HEALTH GREEN VIBRANCE JUNIOR FOR TODDLERS + TWEENS VERSION 4.0 9 BILLION PROBIOTICS PER DOSE FROM 9 STRAINS GREEN SUPERFOOD + PROTEIN + D3 SPECIFICALLY DESIGNED FOR A GROWING CHILD |
| **1001207300** | GARDEN OF LIFE RAW PROBIOTICS KIDS USDA ORGANIC RAW VEGETARIAN RAW WHOLE FOOD |
| **1001147400** | DIGESTIVE ADVANTAGE KIDS DAILY PROBIOTIC GUMMIES |
| **1001257300** | PURITAN'S PRIDE PROBIOTIC ACIDOPHILUS STOMACH FRIENDLY 100 MILLION ACTIVE CULTURES VEGETARIAN |
| **1001187600** | NATURE'S BOUNTY PROBIOTIC GUMMIES 4 BILLION LIVE CULTURES PER SERVING CONTAINS PROBIOTIC STRAIN BACILLUS COAGULANS UNIQUE IS-2 |
| **1001224900** | HYPERBIOTICS PRO-KIDS THE PERFECT CHILDREN'S PROBIOTIC 3 BILLION CFU ONE VERY TINY PEARL PATENTED, TIME-RELEASE, MICRO-PEARLS 100% VEGETARIAN |
| **1001088100** | NATURE MADE DIGESTIVE HEALTH PROBIOTIC 10 BILLION LIVE CELLS ONCE DAILY |
| **1001263500** | CULTURELLE PROBIOTICS DIGESTIVE HEALTH DAILY PROBIOTIC WITH 100% LACTOBACILLUS GG 10 BILLION ACTIVE CULTURES CHEWABLES ONCE DAILY TABLETS |
| **1001241700** | FINEST NUTRITION DIGESTIVE PROBIOTIC MAXIMUM CARE 30 BILLION ACTIVE CULTURES PER CAPSULE ONE PER DAY |
| **1001239900** | RENEW LIFE ULTIMATE FLORA PROBIOTIC FIZZY DRINK MIX 15 BILLION LIVE CULTURES PER SERVING PROBIOTIC |
| **1001241800** | FINEST NUTRITION PROBIOTIC 10 BILLION CFU PER SERVING ONE PER DAY |
| **1000953601** | THE VITAMIN SHOPPE ULTIMATE 10 PROBIOTIC TARGETED RELEASE 13 BILLION |
| **1000530801** | NATURE MADE ACIDOPHILUS PROBIOTICS 1 BILLION LIVE CELLS PER SERVING |
| **1880999601** | DEFAULT LACTOBACILLUS ACIDOPHILUS |
| **1000488704** | RAINBOW LIGHT PRENATAL ONE FOOD-BASED MULTIVITAMIN EASY TO DIGEST WITH PROBIOTICS & ENZYMES |
| **1000798801** | FLORASTORKIDS DAILY PROBIOTIC UNIQUE STRAIN WITH LARGER, STRONGER CELLS THAN OTHER PROBIOTICS SACCHAROMYCES BOULARDII LYO CNCM I-745 SACHETS \| 250 MG \| POWDER |
| **1001224700** | CULTURELLE KIDS CHEWABLES DAILY PROBIOTIC FORMULA 5 BILLION ACTIVE CULTURES! PROBIOTIC WITH NATURALLY SOURCED LACTOBACILLUS GG |
| **1001177000** | BOTANIC CHOICE ACIDOPHILUS 100 MILLION ORGANISMS |
| **1001251800** | VIBRANT HEALTH GREEN VIBRANCE JUNIOR FOR TODDLERS + TWEENS VERSION 4.0 9 BILLION PROBIOTICS PER DOSE FROM 9 STRAINS GREEN SUPERFOOD + PROTEIN + D3 SPECIFICALLY DESIGNED FOR A GROWING CHILD |
| **1001264500** | HYPERBIOTICS PRO-15 THE PERFECT PROBIOTIC 5 BILLION CFU 15 STRAINS 15X MORE EFFECTIVE THAN CAPSULES! PATENTED, TIME-RELEASE MICRO-PEARLS VEGETARIAN NATURAL PROBIOTIC |
| **1001110200** | TRUBIOTICS DAILY PROBIOTIC FROM THE MAKERS OF ONE A DAY BAYER |
| **1000774001** | NATURE'S BOUNTY PROBIOTIC ACIDOPHILUS 100 MILLION ORGANISMS LACTOBACILLUS ACIDOPHILUS 1 PER DAY CAPSULE |
| **1001240000** | RENEW LIFE EVERYDAY ULTIMATE FLORA PROBIOTIC 15 BILLION LIVE CULTURES PER CAPSULE 10 PROBIOTIC STRAINS PROBIOTIC |
| **1000324101** | SHAKLEE OPTIFLORA PREBIOTIC COMPLEX FOS, INULIN, & MORE |
| **1001180900** | LEE SWANSON SIGNATURE LINE ULTIMATE PROBIOTIC FORMULA OVER 66 BILLION ORGANISMS PER CAPSULE |
| **1001274100** | PROBIOTIC AMERICA PERFECT BIOTICS 30 BILLION CFUS 15 STRAINS ROOM TEMP STABLE DAILY PROBIOTIC |
| **1888324001** | DEFAULT PROBIOTIC |
| **1000903101** | PHILLIPS' COLON HEALTH PROBIOTIC CAPS DAILY PROBIOTIC ONE DAILY BAYER |
| **1001224200** | FLORA UDO'S CHOICE INFANT'S PROBIOTIC 6 INFANT & TODDLER-SPECIFIC STRAINS 3 BILLION CELLS AT TIME OF MANUFACTURE |
| **1001187600** | NATURE'S BOUNTY PROBIOTIC GUMMIES 4 BILLION LIVE CULTURES PER SERVING CONTAINS PROBIOTIC STRAIN BACILLUS COAGULANS UNIQUE IS-2 |
| **1001275700** | FLORASTOR DAILY PROBIOTIC UNIQUE STRAIN WITH LARGER, STRONGER CELLS THAN OTHER PROBIOTICS SACCHAROMYCES BOULARDII LYO CNCM I-745 250 MG |
| **1888324001** | DEFAULT PROBIOTIC |
| **1000951202** | SCHIFF DIGESTIVE ADVANTAGE PROBIOTIC GUMMIES POWERED BY GANEDEN BC30 PROBIOTIC |
| **1001240000** | RENEW LIFE EVERYDAY ULTIMATE FLORA PROBIOTIC 15 BILLION LIVE CULTURES PER CAPSULE 10 PROBIOTIC STRAINS PROBIOTIC |
| **1000974101** | CULTURELLE DIGESTIVE HEALTH DAILY PROBIOTIC FORMULA NOW 100% VEGETARIAN ONCE DAILY VEGETARIAN CAPSULES |
| **1001264700** | EQUATE PROBIOTIC GUMMIES DIETARY PROBIOTIC |
| **1888324001** | DEFAULT PROBIOTIC |
| **1001287900** | BODY BIOTICS INTERNATIONAL BODY BIOTICS SBO PROBIOTICS CONSORTIA PROBIOTIC & PREBIOTIC 500 MG |
| **1000951202** | SCHIFF DIGESTIVE ADVANTAGE PROBIOTIC GUMMIES POWERED BY GANEDEN BC30 PROBIOTIC |
| **1000951202** | SCHIFF DIGESTIVE ADVANTAGE PROBIOTIC GUMMIES POWERED BY GANEDEN BC30 PROBIOTIC |
| **1000999601** | NATURE'S BOUNTY ACIDOPHILUS PROBIOTIC 100 MILLION ORGANISMS |
| **1001160300** | BIOTRUST PRO-X10 EQUIVALENT TO 50 BILLION CFU PER DAY! NOW WITH PREFORPRO ADVANCED PROBIOTIC AND GI HEALTH FORMULA |
| **1001034801** | NATURE'S BOUNTY ULTRA STRENGTH PROBIOTIC 10 20 BILLION LIVE PROBIOTIC CULTURES 10 PROBIOTIC STRAINS |
| **1000999601** | NATURE'S BOUNTY ACIDOPHILUS PROBIOTIC 100 MILLION ORGANISMS |
| **1001102301** | TOTAL PROBIOTICS NUTRI-WEST |
| **1001184600** | DR. MERCOLA COMPLETE PROBIOTICS 70 BILLION CFU ACID AND BILE RESISTANT 10 STRAINS |
| **1001077501** | TRUNATURE DIGESTIVE PROBIOTIC 10 BILLION ACTIVE CULTURES |
| **1001180900** | LEE SWANSON SIGNATURE LINE ULTIMATE PROBIOTIC FORMULA OVER 66 BILLION ORGANISMS PER CAPSULE |
| **1001231200** | CVS HEALTH PROBIOTIC FORMULA ACIDOPHILUS 1 BILLION ACTIVE CULTURES |
| **1000910501** | SPRING VALLEY PROBIOTIC MULTI-ENZYME DIGESTIVE FORMULA WITH ACTIVE ACIDOPHILUS CULTURES |
| **1001280700** | GENESIS PURE PROBIOTIC |
| **1000242003** | AMERICAN HEALTH CHEWABLE ACIDOPHILUS AND BIFIDUM ONE BILLION ORGANISMS VEGETARIAN FORMULA |
| **1001218300** | OLLY KIDS HAPPY TUMMY PROBIOTIC, PREBIOTIC & PEPPERMINT |
| **20543** | RENEW LIFE ULTIMATE FLORA KIDS PROBIOTIC 1 BILLION LIVE CULTURES 4 PROBIOTIC STRAINS PROBIOTIC |
| **20304** | CULTURELLE PROBIOTICS KIDS PURELY PROBIOTICS PACKETS PROBIOTIC 1+ YEARS |
| **18288** | RENEW LIFE EVERYDAY ULTIMATE FLORA PROBIOTIC 15 BILLION LIVE CULTURES 10 PROBIOTIC STRAINS PROBIOTIC |
| **20081** | AMERICAN HEALTH PROBIOTIC ACIDOPHILUS 20 BILLION ORGANISMS BIO-ACTIVE MULTI-STRAIN CULTURES |
| **20335** | NEWVITALITY RUBY REDS POMEGRANATE, ACAI & MAQUI A DELICIOUS FRUIT AND VEGETABLE WITH POTENT VITAMINS, MINERALS, ENZYMES, HERBS, NUTRIENTS AND PROBIOTICS 100% DAILY VALUE 7 VITAMINS 8,000+ TOTAL ORAC |
| **20531** | RENEW LIFE ULTIMATE FLORA EVERYDAY PROBIOTIC 15 BILLION LIVE CULTURES 12 PROBIOTIC STRAINS PROBIOTIC |
| **20020** | VITACOST PROBIOTIC 10-20 10 STRAINS/20 BILLION MICROORGANISMS PER SERVING WITH NUTRAFLORA FOS FEATURES VIABLEND GASTRIC ACID BYPASS TECHNOLOGY |
| **16299** | SCHIFF DIGESTIVE ADVANTAGE DAILY PROBIOTIC POWERED BY GANEDEN BC30 PROBIOTIC |
| **18229** | VH ESSENTIALS PROBIOTICS WITH PREBIOTICS & CRANBERRY |
| **16608** | OLLY KIDS' MULTI + PROBIOTIC A BLEND OF VITAMINS A, C, D, E, BS & ZINC |
| **15533** | SPRING VALLEY PROBIOTIC ACIDOPHILUS 1 BILLION ACTIVE CULTURES PER CAPLET |
| **17873** | L'IL CRITTERS PROBIOTIC 1 BILLION CFUS PER SERVING WITH PREBIOTICS PROBIOTIC POWERED BY VITAFUSION |
| **18345** | HEALTH PLUS SUPER COLON CLEANSE NATURAL HERBS & PROBIOTICS 530 MG EACH KEY INGREDIENTS PSYLLIUM HUSK POWDER SENNA LEAF POWDER L. ACIDOPHILUS PAPAYA LEAF |
| **18852** | NORDIC NATURALS PROBIOTIC GUMMIES KIDS 1.5 BILLION LIVE CULTURES WITH PREBIOTIC DIETARY FIBER |
| **5597** | DEFAULT PROBIOTIC |
| **15875** | PURITAN'S PRIDE PROBIOTIC 10 FROM 10 DIFFERENT PROBIOTIC ORGANISMS 20 BILLION ACTIVE CULTURES PER SERVING |
| **19553** | TRUNATURE ADVANCED DIGESTIVE PROBIOTIC IMPROVED FORMULA NOW WITH 12 STRAINS! 10 BILLION ACTIVE CULTURES |
| **20473** | NATURE'S BOUNTY PROBIOTIC GUMMIES 4 BILLION LIVE CULTURES PER SERVING CONTAINS A CLINICALLY STUDIED PROBIOTIC STRAIN |
| **17890** | JARROW FORMULAS WOMEN'S FEM DOPHILUS ORAL PROBIOTIC |
| **18426** | NATURE'S BOUNTY ULTRA STRENGTH PROBIOTIC 10 CONTAINS CLINICALLY STUDIED STRAINS 20 BILLION LIVE PROBIOTIC CULTURES 10 PROBIOTIC STRAINS |
| **18569** | MEMBER'S MARK PROBIOTIC + PREBIOTIC GUMMIES MADE FROM REAL FRUIT 3 BILLION CFUS PER SERVING |
| **15562** | PHILLIPS' COLON HEALTH PROBIOTIC CAPS DAILY PROBIOTIC ONE DAILY BAYER |
| **18956** | TOZAL COMPLETE EYE HEALTH FORMULA FLORAGLO LUTEIN WITH ACTILEASE TECHNOLOGY |
| **18790** | HEALTH PLUS SUPER COLON CLEANSE NATURAL HERBS & PROBIOTICS KEY INGREDIENTS PSYLLIUM HUSK POWDER SENNA LEAF POWDER L. ACIDOPHILUS PAPAYA LEAF NEW LOOK AND IMPROVED FORMULA NOW ONLY ONE 5G SCOOP |
| **15273** | KYO-DOPHILUS PROBIOTIC |
| **19826** | OLLY KIDS MULTI + PROBIOTIC A BLEND OF VITAMINS A, C, D, E, BS & ZINC |
| **20700** | NATURE'S SECRET SUPER CLEANSE EXTRA STRENGTH, 14 HERBS & PROBIOTIC |
| **20141** | NATURE'S WAY FORTIFY AGE 50+ PROBIOTIC +PREBIOTICS EVERYDAY CARE 30 BILLION LIVE PROBIOTIC CULTURES PER CAPSULE 11 PROBIOTIC STRAINS PROBIOTIC |
| **15729** | FLORA UDO'S CHOICE SUPER 5 LOZENGE PROBIOTIC 2 BILLION CELLS AT TIME OF MANUFACTURE |
| **18010** | KYO-DOPHILUS9 A BLEND OF NINE PROBIOTICS PROBIOTIC |
| **18465** | HOUSTON ENZYMES BIOMUVE ENZYME + PROBIOTIC |
| **18871** | SCHIFF DIGESTIVE ADVANTAGE DAILY PROBIOTIC GUMMIES |
| **18725** | SMARTY PANTS KIDS PROBIOTIC COMPLETE PREBIOTIC PROBIOTIC 4 BILLION CFU |
| **18864** | SIGNATURE CARE PROBIOTIC 10X FORMULATED WITH 10 STRAINS OF BACTERIA INCLUDING BIFIDOBACTERIUM INFANTIS |
| **5597** | DEFAULT PROBIOTIC |
| **5597** | DEFAULT PROBIOTIC |
| **5597** | DEFAULT PROBIOTIC |
| **18870** | SCHIFF DIGESTIVE ADVANTAGE KIDS DAILY PROBIOTIC GUMMIES |
| **17839** | NATURE'S SUNSHINE NUTRIBIOME PROBIOTIC ELEVEN 18 BILLION OF 11 STRAINS OF GOOD BACTERIA PROBIOTIC |
| **19023** | RAINBOW LIGHT PRENATAL ONE PLUS SUPERFOODS & PROBIOTICS VEGETARIAN \| ONE TABLET PER DAY MULTIVITAMIN NEW & IMPROVED |
| **18616** | DNA MIRACLES CHEWABLE PROBIOTICS |
| **16734** | CVS HEALTH PROBIOTIC FORMULA ACIDOPHILUS 1 BILLION ACTIVE CULTURES |
| **17680** | SCHIFF DIGESTIVE ADVANTAGE PROBIOTIC GUMMIES POWERED BY GANEDEN BC30 PROBIOTIC |
| **20611** | PLEXUS VITALBIOME 8 PROBIOTIC STRAINS/20 BILLION CFU |
| **18730** | CVS HEALTH ADULT 50+ PROBIOTIC CONTAINS PREBIOTIC FIBER 4 BILLION LIVE BACTERIA CELLS PER CAPSULE 10 STRAINS ONE-A-DAY |
| **5597** | DEFAULT PROBIOTIC |
| **17379** | CVS HEALTH SENIOR PROBIOTIC CONTAINS PREBIOTIC FIBERS AND THE HEALTHY BACTERIA FORMULATED WITH MORE BIFIDOBACTERIUM FOR ADULTS 50+ 10 STRAINS 15 BILLION LIVE BACTERIA CELLS PER CAPSULE ONE-A-DAY |
| **16345** | TRUNATURE DIGESTIVE PROBIOTIC 10 BILLION ACTIVE CULTURES |
| **18765** | RITE AID PHARMACY NATURAL ACIDOPHILUS PROBIOTIC COMPLEX |
| **20590** | GARDEN OF LIFE DR. FORMULATED PROBIOTICS ONCE DAILY WOMEN'S 50 BILLION GUARANTEED 16 PROBIOTIC STRAINS RAW PROBIOTIC |
| **18871** | SCHIFF DIGESTIVE ADVANTAGE DAILY PROBIOTIC GUMMIES |
| **17409** | FLORADIX KINDER LOVE CHILDREN'S MULTIVITAMIN LIQUID HERBAL EXTRACT FORMULA RICH IN VITAMINS A, B, C, D & E GOOD SOURCE OF CALCIUM HERBAL EXTRACTS AND FRUIT JUICES VEGETARIAN LIQUID FORMULA |
| **19239** | FLORADIX IRON + HERBS LIQUID EXTRACT FORMULA RICH IN IRON AND B-VITAMINS VEGETARIAN FORMULA |
| **19837** | RAINBOW LIGHT PRENATAL PETITE MINI-TABLET MADE WITH FRUITS, VEGETABLES & PROBIOTICS VEGETARIAN EASY TO SWALLOW |
| **17092** | PURITAN'S PRIDE PROBIOTIC ACIDOPHILUS STOMACH FRIENDLY 100 MILLION ACTIVE CULTURES VEGETARIAN |
| **18185** | NATROL JUICEFESTIV DAILY FRUIT 23 FRUITS +ANTIOXIDANTS, PROBIOTICS & DIGESTIVE ENZYMES MADE WITH ORGANIC FRUITS |
| **20080** | AMERICAN HEALTH DUAL-ACTION ENZYME PROBIOTIC COMPLEX 9 ACTIVE, NATURALLY-BASED ENZYMES 2 BILLION BIO-ACTIVE PROBIOTIC MICROORGANISMS BROAD SPECTRUM ENZYME ACTIVITY |
| **19790** | JARROW FORMULAS YUM-YUM DOPHILUS SUGAR FREE! (WITH XYLITOL) CLINICALLY DOCUMENTED STRAINS FOR KIDS AND ADULTS 1 BILLION PER SERVING 4 STRAINS PROBIOTIC |
| **18426** | NATURE'S BOUNTY ULTRA STRENGTH PROBIOTIC 10 CONTAINS CLINICALLY STUDIED STRAINS 20 BILLION LIVE PROBIOTIC CULTURES 10 PROBIOTIC STRAINS |
| **18790** | HEALTH PLUS SUPER COLON CLEANSE NATURAL HERBS & PROBIOTICS KEY INGREDIENTS PSYLLIUM HUSK POWDER SENNA LEAF POWDER L. ACIDOPHILUS PAPAYA LEAF NEW LOOK AND IMPROVED FORMULA NOW ONLY ONE 5G SCOOP |
| **18426** | NATURE'S BOUNTY ULTRA STRENGTH PROBIOTIC 10 CONTAINS CLINICALLY STUDIED STRAINS 20 BILLION LIVE PROBIOTIC CULTURES 10 PROBIOTIC STRAINS |
| **18185** | NATROL JUICEFESTIV DAILY FRUIT 23 FRUITS +ANTIOXIDANTS, PROBIOTICS & DIGESTIVE ENZYMES MADE WITH ORGANIC FRUITS |
| **3407** | SHAKLEE BIFIDUS & ACIDOPHILUS OPTIFLORA PROBIOTIC COMPLEX |
| **19807** | MICROBIOME LABS MEGA SPOREBIOTIC PROBIOTIC AND ANTIOXIDANT COMBINATION |
| **19535** | CHILDREN'S ENT-PRO SPECIALIZED PROBIOTIC BIOTICS RESEARCH |
| **20473** | NATURE'S BOUNTY PROBIOTIC GUMMIES 4 BILLION LIVE CULTURES PER SERVING CONTAINS A CLINICALLY STUDIED PROBIOTIC STRAIN |
| **19926** | HUM SKIN HEROES PRE+PROBIOTIC 40 BN ORGANISMS (AT TIME OF MANUFACTURE) 9 STRAINS + KONJAC ROOT |
| **19790** | JARROW FORMULAS YUM-YUM DOPHILUS SUGAR FREE! (WITH XYLITOL) CLINICALLY DOCUMENTED STRAINS FOR KIDS AND ADULTS 1 BILLION PER SERVING 4 STRAINS PROBIOTIC |
| **17385** | NOW PROBIOTIC-10 25 BILLION 10 PROBIOTIC STRAINS CLINICALLY VALIDATED STRAINS VEGETARIAN/VEGAN |
| **17164** | CULTURELLE PROBIOTICS DIGESTIVE HEALTH DAILY PROBIOTIC WITH 100% LACTOBACILLUS GG 10 BILLION ACTIVE CULTURES CHEWABLES ONCE DAILY TABLETS |
| **18745** | CVS HEALTH PROBIOTIC + CALCIUM ACIDOPHILUS OVER 100 MILLION ACTIVE LACTOBACILLUS ACIDOPHILUS |
| **18184** | NATURE'S WAY PRIMADOPHILUS FORTIFY WOMEN'S PROBIOTIC 30 BILLION LIVE PROBIOTIC CULTURES PER CAPSULE DELAYED RELEASE PROBIOTIC |
| **17092** | PURITAN'S PRIDE PROBIOTIC ACIDOPHILUS STOMACH FRIENDLY 100 MILLION ACTIVE CULTURES VEGETARIAN |
| **16638** | CULTURELLE DIGESTIVE HEALTH DAILY PROBIOTIC FORMULA NOW 100% VEGETARIAN ONCE DAILY VEGETARIAN CAPSULES |
| **5597** | DEFAULT PROBIOTIC |
| **14625** | HEALTHY ORIGINS NATURAL PROBIOTIC 30 BILLION CFU'S 8 STRAINS AND 30 BILLION COLONY FORMING UNITS FLORAFIT |
| **5597** | DEFAULT PROBIOTIC |
| **15908** | NATURE'S BOUNTY ACIDOPHILUS PROBIOTIC 100 MILLION ORGANISMS |
| **20611** | PLEXUS VITALBIOME 8 PROBIOTIC STRAINS/20 BILLION CFU |
| **18871** | SCHIFF DIGESTIVE ADVANTAGE DAILY PROBIOTIC GUMMIES |
| **18589** | GNC PROBIOTIC COMPLEX 1 BILLION CFUS 8 UNIQUE STRAINS, INCLUDING CLINICALLY STUDIED PROBIOTICS 1 BILLION ACTIVE CULTURES |
| **19479** | DEFAULT GUMMY BEAR PROBIOTIC |
| **19534** | BIO SCHWARTZ ADVANCED STRENGTH PROBIOTIC 40 BILLION CFU SHELF LIFE POTENCY GUARANTEED NON-GMO INGREDIENTS 100% ALLERGEN FREE SHELF STABLE |
| **16608** | OLLY KIDS' MULTI + PROBIOTIC A BLEND OF VITAMINS A, C, D, E, BS & ZINC |
| **15534** | SPRING VALLEY PROBIOTIC MULTI-ENZYME DIGESTIVE FORMULA WITH ACTIVE ACIDOPHILUS CULTURES |
| **18229** | VH ESSENTIALS PROBIOTICS WITH PREBIOTICS & CRANBERRY |
| **17954** | NUTRATECH PROBIO-15 15 BILLION CFU PROBIOTIC FORMULA PROBIOTIC AND PREBIOTIC |
| **18895** | GERBER SOOTHE PROBIOTIC COLIC DROPS PROBIOTIC |
| **18106** | NATURE'S BOUNTY CHEWABLE PROBIOTIC ACIDOPHILUS 1 BILLION ORGANISMS |
| **19131** | GENERIC3 PROBIOTIC QUATTRO 2 BILLION ACTIVE CELLS AT BEST BY DATE |
| **17385** | NOW PROBIOTIC-10 25 BILLION 10 PROBIOTIC STRAINS CLINICALLY VALIDATED STRAINS VEGETARIAN/VEGAN |
| **16172** | MEMBER'S MARK ULTIMATE PROBIOTIC FORMULA ACIDOPHILUS MADE WITH BIOFLORA 2 BILLION ACTIVE CULTURES 1 CAPLET PER DAY |
| **18881** | THE HONEST CO. BABY + TODDLER MULTI POWDER PREMIUM MULTI-VITAMIN VITAMINS MINERALS SUPERFOODS PROBIOTICS PRE-MEASURED INDIVIDUAL PACKETS |
| **5597** | DEFAULT PROBIOTIC |
| **20150** | GENERIC42 SUPREMA DOPHILUS 5 BILLION CFU PER CAPSULE |
| **15908** | NATURE'S BOUNTY ACIDOPHILUS PROBIOTIC 100 MILLION ORGANISMS |
| **16638** | CULTURELLE DIGESTIVE HEALTH DAILY PROBIOTIC FORMULA NOW 100% VEGETARIAN ONCE DAILY VEGETARIAN CAPSULES |
| **20531** | RENEW LIFE ULTIMATE FLORA EVERYDAY PROBIOTIC 15 BILLION LIVE CULTURES 12 PROBIOTIC STRAINS PROBIOTIC |
| **18287** | RENEW LIFE EXTRA CARE ULTIMATE FLORA PROBIOTIC 50 BILLION LIVE CULTURES 12 PROBIOTIC STRAINS PROBIOTIC |
| **18229** | VH ESSENTIALS PROBIOTICS WITH PREBIOTICS & CRANBERRY |
| **19585** | MEMBER'S MARK 10 STRAIN PROBIOTIC FORMULATED WITH 10 STRAINS OF HEALTHY BACTERIA INCLUDING B. INFANTIS AND L. RHAMNOSUS GG |
| **19247** | GENERIC49 PROBIOTIC COMPLEX WITH ACIDOPHILUS |
| **18426** | NATURE'S BOUNTY ULTRA STRENGTH PROBIOTIC 10 CONTAINS CLINICALLY STUDIED STRAINS 20 BILLION LIVE PROBIOTIC CULTURES 10 PROBIOTIC STRAINS |
| **18096** | GNC MULTI-STRAIN PROBIOTIC COMPLEX 1 BILLION CFUS BLEND OF 8 BENEFICIAL PROBIOTIC STRAINS |
| **18185** | NATROL JUICEFESTIV DAILY FRUIT 23 FRUITS +ANTIOXIDANTS, PROBIOTICS & DIGESTIVE ENZYMES MADE WITH ORGANIC FRUITS |
| **20833** | BIOTRUST PRO-X10 ADVANCED PROBIOTIC & GUT-HEALTH FORMULA EQUIVALENT TO 50 BILLION CFU PER DAY! |
| **20473** | NATURE'S BOUNTY PROBIOTIC GUMMIES 4 BILLION LIVE CULTURES PER SERVING CONTAINS A CLINICALLY STUDIED PROBIOTIC STRAIN |
| **18323** | GERBER GENTLE EVERYDAY PROBIOTIC DROPS PROBIOTIC |
| **19953** | ESSENTIAL BEING CHEWABLE PROBIOTIC (ACIDOPHILUS) |
| **19747** | 365 PROBIOTIC + FIBER GUMMIES 2 BILLION CFU 3G FIBER PER SERVING |
| **17714** | DEFAULT LACTOBACILLUS ACIDOPHILUS |
| **18426** | NATURE'S BOUNTY ULTRA STRENGTH PROBIOTIC 10 CONTAINS CLINICALLY STUDIED STRAINS 20 BILLION LIVE PROBIOTIC CULTURES 10 PROBIOTIC STRAINS |
| **18205** | MOMMY'S BLISS PROBIOTIC DROPS EVERYDAY 1 BILLION CELLS PER SERVING LIQUID PROBIOTIC AGE NEWBORN+ |
| **18881** | THE HONEST CO. BABY + TODDLER MULTI POWDER PREMIUM MULTI-VITAMIN VITAMINS MINERALS SUPERFOODS PROBIOTICS PRE-MEASURED INDIVIDUAL PACKETS |
| **19059** | MOMMY'S BLISS PROBIOTIC POWDER PACKS 5 BILLION CELLS |
| **18288** | RENEW LIFE EVERYDAY ULTIMATE FLORA PROBIOTIC 15 BILLION LIVE CULTURES 10 PROBIOTIC STRAINS PROBIOTIC |
| **18871** | SCHIFF DIGESTIVE ADVANTAGE DAILY PROBIOTIC GUMMIES |
| **18871** | SCHIFF DIGESTIVE ADVANTAGE DAILY PROBIOTIC GUMMIES |
| **18631** | MEGAFOOD MEGAFLORA FOR BABY & ME 30 BILLION ACTIVE CULTURES WITH GINGER & PREBIOTIC FOODS PROBIOTIC |
| **20894** | SPRING VALLEY DAILY PROBIOTIC 4 BILLION CFUS PER CAPSULE 10 STRAINS FORMULATED FOR DAILY USE CONTAINS PREBIOTIC FIBERS AND 10 STRAINS OF BENEFICIAL BACTERIA |
| **17162** | CUSTOM PROBIOTICS HIGH COUNT, MULTI-STRAIN ACIDOPHILUS AND BIFIDUS 60 BILLION CFU'S/CAPSULE ADULT FORMULA CP-1 DAIRY FREE |
| **15338** | ALIGN PROBIOTIC CLINICALLY STUDIED B. INFANTIS 35624 |
| **19558** | TRUNATURE DIGESTIVE PROBIOTIC GUMMIES 3 BILLION ACTIVE CULTURES 2 PROBIOTIC STRAINS DAILY PROBIOTIC FOR ADULTS AND CHILDREN AGES 3+ MADE FROM REAL FRUIT |
| **17714** | DEFAULT LACTOBACILLUS ACIDOPHILUS |
| **18650** | NATURE'S SUNSHINE NUTRIBIOME ELEVEN ELEVATED PROVIDES 30 BILLION CFU OF 11 STRAINS OF GOOD BACTERIA PER SERVING PROBIOTIC |
| **18949** | GREAT HEALTHWORKS PROBIOTICXL A UNIQUE TRIPLE ACTION PROBIOTIC BLEND PHYSICIAN FORMULATED |
| **17911** | WALGREENS PROBIOTIC COLON SUPPORT DAILY PROBIOTIC 3 STRAINS OF GOOD BACTERIA ONCE-DAILY CAPSULES |
| **18010** | KYO-DOPHILUS9 A BLEND OF NINE PROBIOTICS PROBIOTIC |
| **17679** | REPHRESH PRO-B PROBIOTIC FEMININE JUST 1 CAPSULE A DAY! FROM THE MAKERS OF 1 FIRST RESPONSE |
| **20141** | NATURE'S WAY FORTIFY AGE 50+ PROBIOTIC +PREBIOTICS EVERYDAY CARE 30 BILLION LIVE PROBIOTIC CULTURES PER CAPSULE 11 PROBIOTIC STRAINS PROBIOTIC |
| **18889** | COUNTRY LIFE CORE DAILY-1 MULTIVITAMINS 1 DAILY MEN ONE TABLET INCLUDES: 200+ MG OF MEN'S HEALTH BLEND OVER 30 RAW WHOLE FOODS 'COENZYMATED' B VITAMINS AND PROBIOTICS, DIGESTIVE ENZYMES, AND ALOE |
| **18789** | GARDEN OF LIFE MYKIND ORGANICS EXTRA STRENGTH TURMERIC WITH ORGANIC FERMENTED WHOLE TURMERIC & GINGER ORGANIC BLACK PEPPER & PROBIOTICS |
| **19162** | NATURE'S TRUTH VITAMINS PROBIOTIC ACIDOPHILUS 500 MILLION ACTIVE CULTURES 3 MG PER SERVING |
| **18375** | PHILLIPS' COLON HEALTH DAILY PROBIOTIC 3 TYPES OF GOOD BACTERIA ONE DAILY BAYER |
| **18426** | NATURE'S BOUNTY ULTRA STRENGTH PROBIOTIC 10 CONTAINS CLINICALLY STUDIED STRAINS 20 BILLION LIVE PROBIOTIC CULTURES 10 PROBIOTIC STRAINS |
| **16978** | NUCIFIC BIO-X4 4-IN-1 WEIGHT MANAGEMENT PROBIOTIC |
| **19851** | NEWRHYTHM CLINICALLY STUDIED PROBIOTICS 20 STRAINS 50 BILLION CFU ACTIVE CULTURES FORMULATED WITH PREBIOTICS |
| **19543** | PB ASSIST JR PROBIOTIC POWDER DOTERRA |
| **20286** | SOLARAY PASSION FLOWER 350 MG PER CAPSULE WHOLE AERIAL PASSIFLORA INCARNATA |
| **16222** | ISOTONIX DIGESTIVE ENZYMES WITH PROBIOTICS |
| **17901** | NUTRICLEAN PROBIOTICS |
| **19039** | CHILDREN'S PROBIOTIC PACKETS 5 BILLION ACTIVE CULTURES PER SERVING UP&UP |
| **18290** | RENEW LIFE ULTIMATE FLORA BABY PROBIOTIC INFANTS & TODDLERS 4 BILLION LIVE CULTURES 5 SCIENTIFICALLY STUDIED STRAINS PROBIOTIC |
| **18426** | NATURE'S BOUNTY ULTRA STRENGTH PROBIOTIC 10 CONTAINS CLINICALLY STUDIED STRAINS 20 BILLION LIVE PROBIOTIC CULTURES 10 PROBIOTIC STRAINS |
| **17092** | PURITAN'S PRIDE PROBIOTIC ACIDOPHILUS STOMACH FRIENDLY 100 MILLION ACTIVE CULTURES VEGETARIAN |
| **18870** | SCHIFF DIGESTIVE ADVANTAGE KIDS DAILY PROBIOTIC GUMMIES |
| **19965** | PHYSICIAN'S CHOICE 60 BILLION PROBIOTIC WITH PREBIOTIC FIBER BLEND 10 STRAINS |
| **17385** | NOW PROBIOTIC-10 25 BILLION 10 PROBIOTIC STRAINS CLINICALLY VALIDATED STRAINS VEGETARIAN/VEGAN |
| **15913** | NATURE'S BOUNTY ULTRA STRENGTH PROBIOTIC 10 20 BILLION LIVE PROBIOTIC CULTURES 10 PROBIOTIC STRAINS |
| **20302** | CULTURELLE PROBIOTICS KIDS DAILY PROBIOTIC CHEWABLES |
| **5597** | DEFAULT PROBIOTIC |
| **18500** | SPRING VALLEY CHILDREN'S PROBIOTIC GUMMIES |
| **19730** | 1MD COMPLETE PROBIOTICS PLATINUM 51 BILLION POWERFUL COLONY-FORMING UNITS 11 ROBUST PROBIOTIC STRAINS DAIRY-FREE FORMULA DIGESTIVE |
| **16261** | NATURE'S WAY ONCE DAILY PRIMADOPHILUS KIDS TRUE POTENCY 3 BILLION CFU TRUE IDENTITY LACTOBACILLI & BIFIDOBACTERIA AGES 2-12 |
| **15562** | PHILLIPS' COLON HEALTH PROBIOTIC CAPS DAILY PROBIOTIC ONE DAILY BAYER |
| **18790** | HEALTH PLUS SUPER COLON CLEANSE NATURAL HERBS & PROBIOTICS KEY INGREDIENTS PSYLLIUM HUSK POWDER SENNA LEAF POWDER L. ACIDOPHILUS PAPAYA LEAF NEW LOOK AND IMPROVED FORMULA NOW ONLY ONE 5G SCOOP |
| **17870** | DR. DAVID WILLIAMS PROBIOTIC ADVANTAGE COLON HEALTH EXTRA STRENGTH 10 BILLION LIVE PROBIOTICS |
| **17017** | VIBRANT HEALTH GREEN VIBRANCE PLANT-BASED ADVANCED DAILY SUPERFOOD +25 BILLION PROBIOTICS CLINICALLY FORMULATED \| VERSION 15.0 |
| **19646** | YOUNG LIVING ESSENTIAL OILS LIFE 9 PROBIOTIC 17 BILLION ACTIVE CULTURES |
| **15875** | PURITAN'S PRIDE PROBIOTIC 10 FROM 10 DIFFERENT PROBIOTIC ORGANISMS 20 BILLION ACTIVE CULTURES PER SERVING |
| **18426** | NATURE'S BOUNTY ULTRA STRENGTH PROBIOTIC 10 CONTAINS CLINICALLY STUDIED STRAINS 20 BILLION LIVE PROBIOTIC CULTURES 10 PROBIOTIC STRAINS |
| **18426** | NATURE'S BOUNTY ULTRA STRENGTH PROBIOTIC 10 CONTAINS CLINICALLY STUDIED STRAINS 20 BILLION LIVE PROBIOTIC CULTURES 10 PROBIOTIC STRAINS |
| **19474** | RAINBOW LIGHT WOMEN'S ONE PLUS SUPERFOODS & PROBIOTICS VEGETARIAN MULTIVITAMIN |
| **16734** | CVS HEALTH PROBIOTIC FORMULA ACIDOPHILUS 1 BILLION ACTIVE CULTURES |
| **19479** | DEFAULT GUMMY BEAR PROBIOTIC |
| **19454** | CVS HEALTH DIGESTIVE PROBIOTIC CONTAINS PREBIOTIC FIBERS FORMULATED WITH 4 STRAINS OF HEALTHY BACTERIA 2 BILLION LIVE BACTERIA CELLS PER CAPSULE |
| **19725** | NORDIC NATURALS PRODHA EYE 360 EPA/845 DHA HIGH LEVELS OF FLORAGLO LUTEIN AND ZEAXANTHIN 1000 MG |
| **18426** | NATURE'S BOUNTY ULTRA STRENGTH PROBIOTIC 10 CONTAINS CLINICALLY STUDIED STRAINS 20 BILLION LIVE PROBIOTIC CULTURES 10 PROBIOTIC STRAINS |
| **19804** | METAGENICS ULTRAFLORA RESTORE PROBIOTIC |
| **18920** | AIM FLORAFOOD PROBIOTIC FORMULA WITH THREE BILLION LIVE CELLS AIM THE AIM COMPANIES |
| **17912** | WALGREENS PROBIOTIC WITH LACTOBACILLUS RHAMNOSUS GG |
| **20589** | GARDEN OF LIFE DR. FORMULATED PROBIOTICS ONCE DAILY MEN'S 50 BILLION GUARANTEED 15 PROBIOTIC STRAINS RAW PROBIOTIC |
| **20304** | CULTURELLE PROBIOTICS KIDS PURELY PROBIOTICS PACKETS PROBIOTIC 1+ YEARS |
| **15963** | THE VITAMIN SHOPPE ULTIMATE 10 PROBIOTIC TARGETED RELEASE 13 BILLION |
| **19067** | RAINBOW LIGHT MEN'S ONE PLUS SUPERFOODS & PROBIOTICS VEGETARIAN ONE TABLET PER DAY MULTIVITAMIN |
| **19848** | GARDEN OF LIFE DR. FORMULATED PROBIOTICS ORGANIC KIDS DAILY CARE 5 BILLION CFU GUARANTEED 14 PROBIOTIC STRAINS +VITAMINS C & D NO SUGAR ADDED PROBIOTIC |
| **18871** | SCHIFF DIGESTIVE ADVANTAGE DAILY PROBIOTIC GUMMIES |
| **16462** | RAINBOW LIGHT WOMEN'S ONE FOOD-BASED MULTIVITAMIN EASY TO DIGEST WITH PROBIOTICS & ENZYMES |
| **18532** | SPRING VALLEY PREBIOTIC PLUS PROBIOTIC 2 BILLION ACTIVE CULTURES PER CAPSULE 10 STRAINS |
| **17164** | CULTURELLE PROBIOTICS DIGESTIVE HEALTH DAILY PROBIOTIC WITH 100% LACTOBACILLUS GG 10 BILLION ACTIVE CULTURES CHEWABLES ONCE DAILY TABLETS |
| **20559** | RENEW LIFE MOOD & STRESS PROBIOTIC 3 BILLION LIVE CULTURES 2 PROBIOTIC STRAINS PROBIOTIC |
| **20560** | RENEW LIFE ULTIMATE FLORA PROBIOTIC WOMEN'S CARE 25 BILLION LIVE CULTURES 10 PROBIOTIC STRAINS PROBIOTIC |
| **18278** | SOLARAY MULTIDOPHILUS POWDER 3 TRIPLE STRAIN FORMULA 5 BILLION CFU UNFLAVORED, DAIRY FREE FREEZE DRIED |
| **19291** | JARROW FORMULAS JARRO-DOPHILUS ORIGINAL MULTI-STRAIN PROBIOTIC 6 CLINICALLY DOCUMENTED PROBIOTIC STRAINS 3.4 BILLION LIVE BACTERIA PROBIOTIC ORIGINAL JARRO-DOPHILUS FORMULA |
| **20226** | ZENWISE HEALTH DAILY DIGESTIVE ENZYMES WITH PREBIOTICS + PROBIOTICS + PLANT-SOURCED BLEND |
| **19562** | NATURE MADE DIGESTIVE PROBIOTICS 15 BILLION LIVE CULTURES PER SERVING ADVANCED DUAL ACTION |
| **20335** | NEWVITALITY RUBY REDS POMEGRANATE, ACAI & MAQUI A DELICIOUS FRUIT AND VEGETABLE WITH POTENT VITAMINS, MINERALS, ENZYMES, HERBS, NUTRIENTS AND PROBIOTICS 100% DAILY VALUE 7 VITAMINS 8,000+ TOTAL ORAC |
| **18870** | SCHIFF DIGESTIVE ADVANTAGE KIDS DAILY PROBIOTIC GUMMIES |
| **5597** | DEFAULT PROBIOTIC |
| **18614** | CULTURELLE PROBIOTICS BABY GROW + THRIVE PROBIOTICS + VITAMIN D DROPS 0-12 MONTHS PROBIOTIC WITH VITAMIN D |
| **16638** | CULTURELLE DIGESTIVE HEALTH DAILY PROBIOTIC FORMULA NOW 100% VEGETARIAN ONCE DAILY VEGETARIAN CAPSULES |
| **16919** | GARDEN OF LIFE DR. FORMULATED PROBIOTICS ORGANIC KIDS+ PROBIOTICS + VITAMINS C & D 5 BILLION GUARANTEED 14 PROBIOTIC STRAINS USDA ORGANIC NO SUGAR ADDED PROBIOTIC |
| **18975** | NUTRITION ESSENTIALS PROBIOTIC DIGESTIVE HEALTH SUPPORT FORMULA |
| **19982** | ANDREW LESSMAN PROCAPS ULTIMATE FRIENDLY FLORA 25 BILLION CFU COMPREHENSIVE PROBIOTIC COMPLEX FIVE BENEFICIAL PROBIOTIC STRAINS |
| **20304** | CULTURELLE PROBIOTICS KIDS PURELY PROBIOTICS PACKETS PROBIOTIC 1+ YEARS |
| **20303** | CULTURELLE PROBIOTICS KIDS PROBIOTIC GUMMIES |
| **16638** | CULTURELLE DIGESTIVE HEALTH DAILY PROBIOTIC FORMULA NOW 100% VEGETARIAN ONCE DAILY VEGETARIAN CAPSULES |
| **20901** | VIBRANT HEALTH MAXIMUM VIBRANCE CONTAINS ALL KNOWN NUTRIENTS PLANT-BASED MULTI- ADVANCED DAILY FUTUREFOOD CLINICALLY FORMULATED \| VERSION 6.1 20 G PLANT PROTEIN 25 BILLION PROBIOTICS MULTIVITAMIN |
| **17911** | WALGREENS PROBIOTIC COLON SUPPORT DAILY PROBIOTIC 3 STRAINS OF GOOD BACTERIA ONCE-DAILY CAPSULES |
| **18851** | NORDIC NATURALS OMEGA VISION 1460 MG OMEGA-3 WITH ZEAXANTHIN & FLORAGLO LUTEIN SUPERIOR TRIGLYCERIDE FORM 1000 MG |
| **19531** | NANOFLORA PROBIOTIC BIOPHARMA SCIENTIFIC |
| **20280** | NATURE'S WAY ONCE DAILY PRIMADOPHILUS KIDS TRUE POTENCY 3 BILLION CFU TRUE IDENTITY LACTOBACILLI & BIFIDOBACTERIA AGES 2-12 |
| **18205** | MOMMY'S BLISS PROBIOTIC DROPS EVERYDAY 1 BILLION CELLS PER SERVING LIQUID PROBIOTIC AGE NEWBORN+ |
| **19874** | VSL#3 THE LIVING SHIELD PROBIOTIC 450 BILLION ALL-NATURAL, LIVE, FREEZE-DRIED LACTIC ACID BACTERIA PER PACKET |
| **17868** | MULTI-PROBIOTIC 40 BILLION DOUGLAS LABORATORIES |
| **19101** | BIOTE MEDICAL BIOTE PROBIOTIC |
| **18589** | GNC PROBIOTIC COMPLEX 1 BILLION CFUS 8 UNIQUE STRAINS, INCLUDING CLINICALLY STUDIED PROBIOTICS 1 BILLION ACTIVE CULTURES |
| **15534** | SPRING VALLEY PROBIOTIC MULTI-ENZYME DIGESTIVE FORMULA WITH ACTIVE ACIDOPHILUS CULTURES |
| **19067** | RAINBOW LIGHT MEN'S ONE PLUS SUPERFOODS & PROBIOTICS VEGETARIAN ONE TABLET PER DAY MULTIVITAMIN |
| **18426** | NATURE'S BOUNTY ULTRA STRENGTH PROBIOTIC 10 CONTAINS CLINICALLY STUDIED STRAINS 20 BILLION LIVE PROBIOTIC CULTURES 10 PROBIOTIC STRAINS |
| **15533** | SPRING VALLEY PROBIOTIC ACIDOPHILUS 1 BILLION ACTIVE CULTURES PER CAPLET |
| **20561** | RENEW LIFE WOMEN'S DAILY 2-IN-1 PREBIOTICS + PROBIOTICS 20 BILLION LIVE CULTURES 10 PROBIOTIC STRAINS PROBIOTIC |
| **18565** | MYOVITE MULTIVITAMIN MULTIMINERAL MYOGENIX PACKETS VITAMINS CHEALTED MINERALS DIGESTIVE ENZYMES PROBIOTICS GREEN FOOD NUTRIENTS PERFORMANCE OPTIMIZERS |
| **19076** | VITAMIN WORLD PROBIOTIC10 10 PROBIOTIC ORGANISMS CONTAINS 20 BILLION LIVE PROBIOTIC CULTURES |
| **17379** | CVS HEALTH SENIOR PROBIOTIC CONTAINS PREBIOTIC FIBERS AND THE HEALTHY BACTERIA FORMULATED WITH MORE BIFIDOBACTERIUM FOR ADULTS 50+ 10 STRAINS 15 BILLION LIVE BACTERIA CELLS PER CAPSULE ONE-A-DAY |
| **18752** | RITE AID PHARMACY SACCHAROMYCES BOULARDII PROBIOTIC 250 MG PER SERVING |
| **15671** | PB8 ORIGINAL FORMULA WITH LACTOBACILLUS & BIFIDOBACTERIUM SPECIES |
| **16978** | NUCIFIC BIO-X4 4-IN-1 WEIGHT MANAGEMENT PROBIOTIC |
| **18871** | SCHIFF DIGESTIVE ADVANTAGE DAILY PROBIOTIC GUMMIES |
| **16462** | RAINBOW LIGHT WOMEN'S ONE FOOD-BASED MULTIVITAMIN EASY TO DIGEST WITH PROBIOTICS & ENZYMES |
| **15908** | NATURE'S BOUNTY ACIDOPHILUS PROBIOTIC 100 MILLION ORGANISMS |
| **19646** | YOUNG LIVING ESSENTIAL OILS LIFE 9 PROBIOTIC 17 BILLION ACTIVE CULTURES |
| **16636** | CULTURELLE KIDS CHEWABLES DAILY PROBIOTIC FORMULA 5 BILLION ACTIVE CULTURES! PROBIOTIC WITH NATURALLY SOURCED LACTOBACILLUS GG |
| **5597** | DEFAULT PROBIOTIC |
| **15562** | PHILLIPS' COLON HEALTH PROBIOTIC CAPS DAILY PROBIOTIC ONE DAILY BAYER |
| **16608** | OLLY KIDS' MULTI + PROBIOTIC A BLEND OF VITAMINS A, C, D, E, BS & ZINC |
| **18871** | SCHIFF DIGESTIVE ADVANTAGE DAILY PROBIOTIC GUMMIES |
| **17680** | SCHIFF DIGESTIVE ADVANTAGE PROBIOTIC GUMMIES POWERED BY GANEDEN BC30 PROBIOTIC |
| **20067** | RESTORA LACTOBACILLUS CASEI KE-99 ENHANCED WITH OMEGA-3 1 A DAY PROBIOTIC |
| **17952** | FLORA TODDLER'S PROBIOTIC 6 INFANT & TODDLER-SPECIFIC STRAINS 3 BILLION CELLS AT TIME OF MANUFACTURE RAW PROBIOTICS |
| **18479** | CVS HEALTH ADVANCED PROBIOTIC GUMMIES BACILLUS COAGULANS DAILY PROBIOTIC EASY-TO-TAKE GUMMIES |
| **16608** | OLLY KIDS' MULTI + PROBIOTIC A BLEND OF VITAMINS A, C, D, E, BS & ZINC |
| **16637** | CULTURELLE KIDS PACKETS DAILY PROBIOTIC FORMULA 5 BILLION ACTIVE CULTURES! PROBIOTIC WITH NATURALLY SOURCED LACTOBACILLUS GG |
| **19463** | NATURE MADE DIGESTIVE PROBIOTICS 10 BILLION LIVE CULTURES PER SERVING |
| **5597** | DEFAULT PROBIOTIC |
| **20301** | CULTURELLE PROBIOTICS BABY GROW + THRIVE PROBIOTIC WITH VITAMIN D |
| **17876** | JARROW FORMULAS JARRO-DOPHILUS + FOS POWDER WITH CLINICALLY DOCUMENTED STRAINS 6 BENEFICIAL PROBIOTIC STRAINS 12 BILLION PER GRAM PROBIOTIC |
| **19239** | FLORADIX IRON + HERBS LIQUID EXTRACT FORMULA RICH IN IRON AND B-VITAMINS VEGETARIAN FORMULA |
| **19474** | RAINBOW LIGHT WOMEN'S ONE PLUS SUPERFOODS & PROBIOTICS VEGETARIAN MULTIVITAMIN |
| **20618** | GENERIC3 MULTI-FLORA PROBIOTIC 16 BILLION ACTIVE CELLS |
| **20666** | CULTURELLE PROBIOTICS PRO-WELL HEALTH & WELLNESS 15 BILLION ACTIVE CULTURES ONCE DAILY VEGETARIAN CAPSULES |
| **18789** | GARDEN OF LIFE MYKIND ORGANICS EXTRA STRENGTH TURMERIC WITH ORGANIC FERMENTED WHOLE TURMERIC & GINGER ORGANIC BLACK PEPPER & PROBIOTICS |
| **18291** | RENEW LIFE WOMEN'S COMPLETE ULTIMATE FLORA PROBIOTIC 90 BILLION LIVE CULTURES 12 PROBIOTIC STRAINS PROBIOTIC |
| **15534** | SPRING VALLEY PROBIOTIC MULTI-ENZYME DIGESTIVE FORMULA WITH ACTIVE ACIDOPHILUS CULTURES |
| **15963** | THE VITAMIN SHOPPE ULTIMATE 10 PROBIOTIC TARGETED RELEASE 13 BILLION |
| **19534** | BIO SCHWARTZ ADVANCED STRENGTH PROBIOTIC 40 BILLION CFU SHELF LIFE POTENCY GUARANTEED NON-GMO INGREDIENTS 100% ALLERGEN FREE SHELF STABLE |
| **19553** | TRUNATURE ADVANCED DIGESTIVE PROBIOTIC IMPROVED FORMULA NOW WITH 12 STRAINS! 10 BILLION ACTIVE CULTURES |
| **18465** | HOUSTON ENZYMES BIOMUVE ENZYME + PROBIOTIC |
| **20473** | NATURE'S BOUNTY PROBIOTIC GUMMIES 4 BILLION LIVE CULTURES PER SERVING CONTAINS A CLINICALLY STUDIED PROBIOTIC STRAIN |
| **18375** | PHILLIPS' COLON HEALTH DAILY PROBIOTIC 3 TYPES OF GOOD BACTERIA ONE DAILY BAYER |
| **19240** | FLORADIX IRON TABLETS IRON AND VITAMINS FORMULA IN A BASE OF HERBS |
| **16608** | OLLY KIDS' MULTI + PROBIOTIC A BLEND OF VITAMINS A, C, D, E, BS & ZINC |
| **19236** | GENERIC11 PROBIOTIC ACIDOPHILUS |
| **17099** | REXALL PROBIOTIC ACIDOPHILUS 100 MILLION ACTIVE CULTURES |
| **20398** | OPTIMAL FLORA PLUS FULL INTESTINAL TRACT COLONIZATION OHS OPTIMAL HEALTH SYSTEMS |
| **18208** | SWANSON PROBIOTICS PRUNE REGULARITY COMPLEX WITH PROBIOTICS & FOS 5 BILLION CFU |
| **17182** | GENERIC48 ALL NATURAL ACIDOPHILUS HIGH POTENCY |
